# The Chinese herbal medicine Dai-Zong-Fang promotes browning of white adipocytes *in vivo* and *in vitro* by activating PKA pathway to ameliorate obesity

**DOI:** 10.3389/fphar.2023.1176443

**Published:** 2023-05-10

**Authors:** Jing Xu, Li-Wei Zhang, Hui Feng, Yang Tang, Shou-Qiang Fu, Xi-Ming Liu, Xiao-Yun Zhu

**Affiliations:** ^1^ Department of Laboratory of Diabetes, Guang’anmen Hospital, China Academy of Chinese Medical Sciences, Beijing, China; ^2^ School of Chinese Medicine, School of Integrated Chinese and Western Medicine, Nanjing University of Chinese Medicine, Nanjing, Jiangsu, China; ^3^ School of Chinese Medicine, Beijing University of Chinese Medicine, Beijing, China

**Keywords:** traditional Chinese medicine, obesity, browning, obese mice, 3T3-L1 adipocytes, Dai-Zong-Fang formula

## Abstract

**Introduction:** The global prevalence of obesity is rising rapidly. Conversion of white adipose tissue (WAT) into beige adipose tissue with heat-consuming characteristics, i.e., WAT browning, effectively inhibits obesity. Dai-Zong-Fang (DZF), a traditional Chinese medicine formula, has long been used to treat metabolic syndrome and obesity. This study aimed to explore the pharmacological mechanism of DZF against obesity.

**Methods:** *In vivo*, C57BL/6J mice were fed high-fat diets to establish the diet-induced obese (DIO) model. DZF (0.40 g/kg and 0.20 g/kg) and metformin (0.15 g/kg, positive control drug) were used as intervention drugs for six weeks, respectively. The effects of DZF on body size, blood glucose and lipid level, structure and morphology of adipocytes and browning of inguinal WAT (iWAT) in DIO mice were observed. *In vitro*, mature 3T3-L1 adipocytes were used as the model. Concentrations of DZF (0.8 mg/mL and 0.4 mg/mL) were selected according to the Cell Counting Kit-8 (CCK8). After 2d intervention, lipid droplet morphology was observed by BODIPY493/503 staining, and mitochondria number was observed by mito-tracker Green staining. H-89 dihydrochloride, a PKA inhibitor, was used to observe the change in browning markers′ expression. The expression levels of browning markers UCP1 and PGC-1α and key molecules of PKA pathway were detected *in vivo* and *in vitro*.

**Results:** *In vivo*, compared with vehicle control group, 0.40 g/kg DZF significantly reduced obesity in DIO mice from body weight, abdomen circumference, Lee′s index, and WAT/body weight (*p* < 0.01 or *p* < 0.001). 0.40 g/kg DZF also significantly reduced fasting blood glucose (FBG), serum triglycerides (TG), total cholesterol (TC), and low-density lipoprotein cholesterol (LDL-C) (*p* < 0.01 or *p* < 0.001). The iWAT′s morphology and mitochondria were browning after DZF intervention. In HE-staining, the lipid droplets became smaller, and the number of mitochondria increased. The mitochondrial structure was remodeled under the electron microscope. The expression of UCP1, PGC-1α and PKA was elevated in iWAT detected by RT-qPCR (*p* < 0.05 or *p* < 0.001). In vitro, compared with the control group, 0.8 mg/mL DZF intervention significantly increased the number of mitochondria and expression of UCP1, PGC-1α, PKA, and pCREB (*p* < 0.05 or *p* < 0.01). In contrast, UCP1 and PGC-1α expression were significantly reversed after adding PKA inhibitor H-89 dihydrochloride.

**Conclusion:** DZF can promote UCP1 expression by activating the PKA pathway, thereby promoting browning of WAT, attenuating obesity, and reducing obesity-related glucose and lipid metabolism abnormalities, indicating that DZF has the potential to be selected as an anti-obesity drug to benefit obese patients.

## 1 Introduction

Obesity is one of the significant health hazards in the world today (Blüher, 2019; Zeng et al., 2021). The global prevalence of obesity was reported to be 12% in 2015 and is expected to reach 18% by 2025 (NCD Risk Factor Collaboration, 2016; GBD 2015 Obesity Collaborators et al., 2017). Notably, obesity is a high-risk factor for type 2 diabetes mellitus, cardiovascular disease and malignancy (Bhupathiraju and Hu, 2016; The Lancet Diabetes Endocrinology, 2020; Klein et al., 2022). The main characteristic of obesity is excessive accumulation of white adipose tissue (WAT), meaning an imbalance between energy storage and expenditure. Under conditions of increased energy expenditure, such as cold and exercise, WAT can be transformed into beige adipocytes with brown adipose tissue (BAT) features, known as “browning” of WAT. Beige adipocytes improve energy metabolism by enhancing non-shivering thermogenesis. Promoting the browning of WAT is a research hotspot of obesity intervention (Yin et al., 2022).

Uncoupling protein 1 (UCP1) is a mitochondrial membrane protein mainly distributed in BAT, also the primary browning marker. UCP1 is increased in activated beige adipocytes and decouples the respiratory chain to promote thermogenesis (Ikeda and Yamada, 2020). Peroxisome proliferators-activated receptor *γ* coactivator-1 alpha (PGC-1α) is a transcriptional coactivator that induces UCP1 expression and is the major thermogenesis factor in adipocytes (Cheng et al., 2018). Protein kinase A (PKA) signaling is a typical energy metabolic pathway that regulates heat generation (Zhang et al., 2021). Several studies have suggested that the PKA signaling pathway directly or indirectly affects the expression of UCP1 and PGC-1α. It plays an important role in browning and is the focus of anti-obesity research.

Traditional Chinese medicine (TCM) is a hot spot in anti-obesity research and plays an essential role in WAT browning (Martel et al., 2017; Ma et al., 2022). Dai-Zong-Fang (DZF) is an herbal formula based on TCM theory and a drug in China’s “Twelfth Five-Year” major New Drug Creation Project. DZF evolved from Xiao-xian-xiong decoction, a famous TCM prescription recorded in the ancient book of TCM “Treatise on Cold Damage Diseases”. It has the traditional effect of clearing phlegm and heat. It was originally used to treat “thoracic accumulation,” and then has been applied to the treatment of metabolic syndrome (MS), obesity, type 2 diabetes mellitus (T2DM), and other metabolic diseases in TCM clinics (Wang et al., 2017; Yang et al., 2021; Wang et al., 2022). DZF consists of six Chinese medicine, Coptis chinensis Franch. (Huanglian), Citrus×aurantium L. (Zhishi), Pinellia ternata (Thunb.) Makino (Banxia), Trichosanthes kirilowii Maxim (Gualou), Neolitsea cassia (L.) Kosterm. (Rougui) and red yeast rice (Hongqu).

In the clinical epidemiological investigation of “Comprehensive Behavioral Intervention Technique for Metabolic Syndrome—Intervention Technique for Traditional Chinese Medicine” funded by the National “Eleventh Five-Year Plan” Science and Technology Support Plan, 3,398 cases of MS patients were included in our research group, among which 1145 cases (33.7%) were the syndrome of combined phlegm and heat, accounting for the highest proportion (Zhao et al., 2016). The common symptoms of MS patients with “the syndrome of combined phlegm and heat” are as follows: large belly, tight abdominal wall, muscular, oily face, red lips, oily hair, sticky stool, and yellow greasy tongue coating. Based on the theory of simultaneous treatment of different diseases in TCM, DZF has obvious curative effect on MS which is dominated by phlegm-heat syndrome. In recent years, our team has researched the effect of DZF on improving metabolism. A multi-center clinical study was carried out to observe the effect of DZF on 38 MS patients characterized by abdominal obesity. After 8 weeks, body weight, waist, and hip circumference were significantly lower than before treatment. Blood glucose, serum triglycerides (TG), and total cholesterol (TC) were significantly decreased (Zhu et al., 2017). In addition, we further explored the pharmacological mechanism of DZF. In C57BL/Ksj-Lepr db^−/−^ (db/db) mice, DZF improved insulin sensitivity by activating the AMPK pathway in the liver and skeletal muscle (Zhu et al., 2018). DZF promotes glucose consumption and regulates lipid metabolism in 3T3-L1 adipocytes (Zhu et al., 2015b). Another research team has also demonstrated the effect of DZF on improving insulin resistance and dyslipidemia, reducing inflammation and liver damage in diet-induced MS mice (Huang et al., 2022).

To further explore the mechanism of DZF in reducing obesity and related disorders of glycolipid metabolism, diet-induced obese (DIO) C57BL/6J mice and mature 3T3-L1 adipocytes were selected as the model in this study. Since previous studies mainly observed the improvement effect of MS, metformin was selected as the positive control drug in this study to compare and observe the effect of DZF on glucose and lipid metabolism in DIO mice.

## 2 Materials and methods

### 2.1 Preparation of DZF extract

**FIGURE 1:**
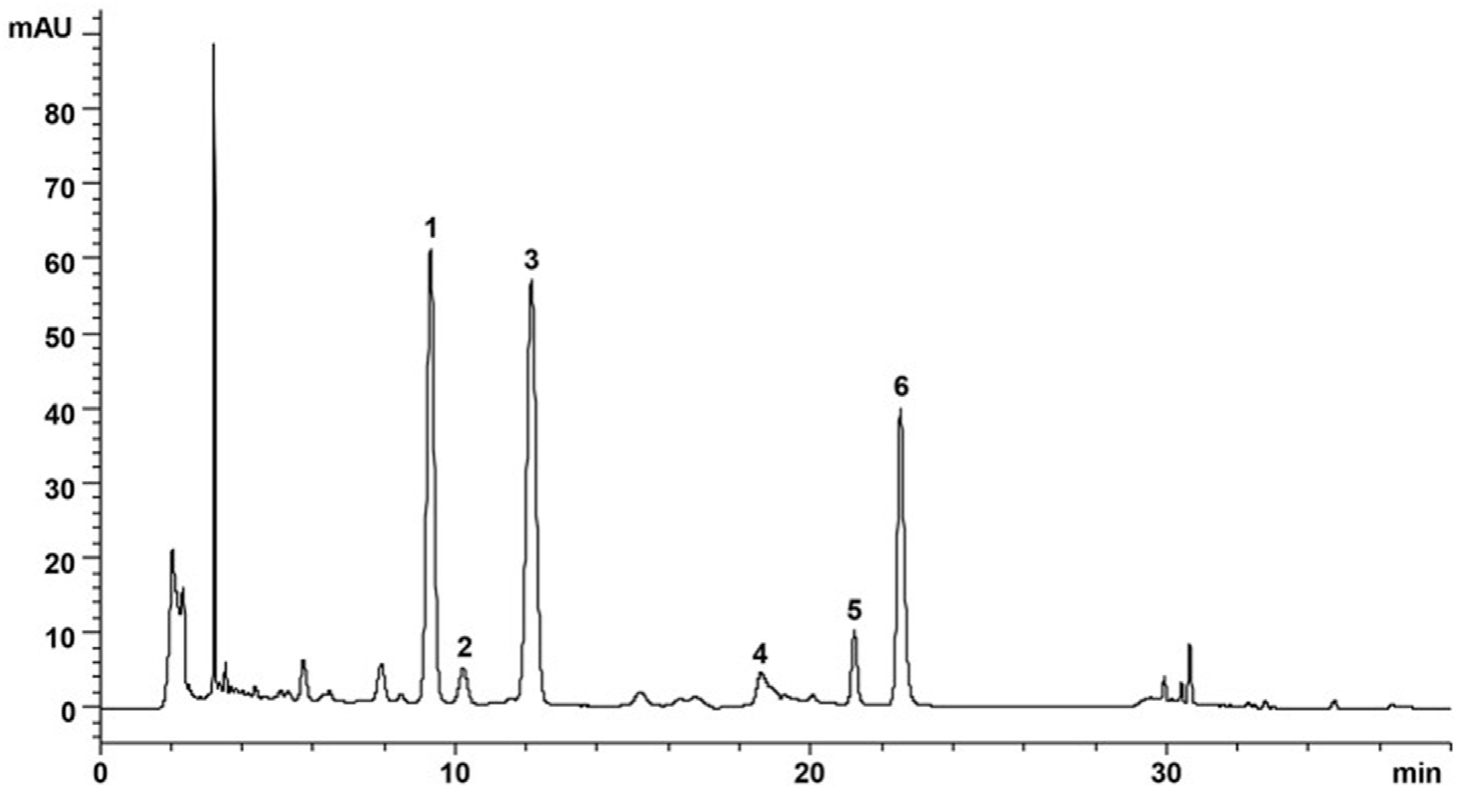
Representative chromatogram of major compounds in DZF. 1. Naringin; 2. Hesperidin; 3. Neohesperidin; 4. Jatrorrhizine; 5. Palmatine; 6. Berberine. [The Figure is quoted from (Zhu et al., 2018)].

DZF extract (freeze-dried powder) was produced and supplied by Zhejiang Jiu Xu Pharmaceutical Co., LTD (Batch Number: YC20200501). Specific preparation method of freeze-dried powder: The names, proportions, and extraction methods of the six Chinese herbs used in DZF are shown in Table 1. *Coptis chinensis* Franch was extracted with acid water. The other five herbs were extracted using ethanol, and ethanol was subsequently recovered by decompression. Under the ultra-low temperature environment of liquid nitrogen, the above extracts were dried, pulverized, and mixed evenly to obtain the freeze-dried DZF extract powder. Compared with traditional methods, the freeze-dried powder can help preserve the original properties of drugs, reduce the damage of ingredients, dissolve quickly, and preserve easily. The extraction method and quality control details are in the Chinese patent document entitled “a Traditional Chinese medicine Composition and Preparation for the treatment of metabolic Syndrome” (Chinese Patent No. ZL201811080557.5). The plant names in this article have been checked with “The World Flora Online” (http://www.worldfloraonline.org, website access date: 2023-02-09). The active components of DZF were determined by high-performance liquid chromatography (HPLC), such as berberine, naringin, hesperidin, and lovastatin. Figure 1 (Zhu et al., 2018) shows the chromatograms of the primarily identified and quantified components of DZF. The quality of the DZF extract is stable. DZF solution with corresponding concentration was prepared by dissolving DZF freeze-dried powder in ultra-pure water and accelerating dissolution by ultrasonic, which was used for *in vivo* experiment intervention. The DZF solution in the *in vitro* experiment was transferred to a super clean table after dissolution and disinfected with a 0.22 μm microporous filtration membrane for bacteria removal.

**TABLE 1:** Ratio of DZF’s ingredients and extraction methods.

Name	Weight ratio (%)	Method
*Coptis chinensis* Franch. (Huanglian)	19.6	Acid water extraction
*Citrus×aurantium* L. (Zhishi)	19.6	Ethanol extraction
*Pinellia ternata* (Thunb.) Makino (Banxia)	19.6	Ethanol extraction
*Trichosanthes kirilowii* Maxim (Gualou)	26.1	Ethanol extraction
*Neolitsea cassia* (L.) Kosterm. (Rougui)	2.1	Ethanol extraction
red yeast rice (Hongqu)	13.0	Ethanol extraction

### 2.2 Animals

All experiment procedures were approved by the Animal Experimentation Ethics Committee of Guang’anmen Hospital, China Academy of Chinese Medical Sciences (Beijing, China, Ethics statement approval number: IAUC-GAMH-2019-009). A total of 117 six-week-old C57BL/6J mice (Weight: 18-22g) were purchased from Beijing HFK Bioscience Co., LTD, Institute of Laboratory Animal Sciences, Chinese Academy of Medical Sciences (Beijing, China, Certificate of Conformity No. 11401300085453). Multiple studies have shown that male C57BL/6J mice are susceptible to DIO than females (Hwang et al., 2010; Benz et al., 2012; Yang et al., 2014). Animals (6 mice/cage) were housed at 25°C ± 1°C, 55%-65% relative humidity, and a light/dark cycle of 12 h/12 h. All mice were given free access to food and drinking water. Best efforts were made to minimize mice suffering. 12 mice were randomly selected and given normal control diets (NCD) (SPF Biotechnology Co., Ltd., Beijing, SPF-F02-001), and all the remaining 105 mice were fed high-fat diets (HFD) (Open Source Diets, United States, D12492, 60 kcal% Fat; including fat, protein, carbohydrate, fiber, mineral, and Vitamin, Details of ingredients: https://researchdiets.com/en/formulas/d12492) for DIO model. During the 10-week modeling period, the body weight of the mice was measured and recorded at a fixed time every week. The mice met the criteria of the DIO model: the body weight of mice≥the average body weight of NCD group mice×120%. After 10 weeks, a total of 80 mice met the modeling criteria. DIO mice were randomly divided into four groups (*n* = 12). The four groups of obese mice continued to receive HFD, including vehicle control group (ultra-pure water, 5 mL/kg, HFD + Veh), positive control group (Metformin, BMS, Shanghai, 0.15 g/kg, HFD + Met), DZF group (low-dose: 0.20 g/kg, high-dose: 0.40 g/kg, HFD + DZF-L/HFD + DZF-H). The mice fed NCD were normal control group (*n* = 12) (ultra-pure water, 5 mL/kg, NCD). The mice were also weighed at a set time each week. After completing a 6-week medication intervention, mice were fasted for 12 h and sacrificed under anesthesia. Blood samples and adipose tissue were collected. The adipose tissue was weighted and frozen at −80°C or fixed in paraformaldehyde and glutaraldehyde.

### 2.3 Cells

#### 2.3.1 Cell culture

The 3T3-L1 cell line was purchased from the Institute of Basic Medical Sciences, Chinese Academy of Medical Sciences. Cells were cultured with 10% NBCS-DMEM until complete fusion, continued contact inhibition for 2 d, and then replaced with induction solution A (0.5 mM IBMX, 1.0 μM DEX, 10 μg/mL insulin) to start induction of differentiation. After 3 d of incubation, it was replaced with induction solution B (10 μg/mL insulin). After 2 d culture, it was replaced with 10% FBS-DMEM for further culture, and the liquid was kept changing every 2-3 days. By the 10th day of induced differentiation, about 90% of the cells were differentiated and mature. According to Cell Counting Kit-8 (CCK8) to detect the effect of DZF on cell growth, the concentration of DZF with no significant inhibition on cell growth was selected. Several concentrations were detected and finally 0.40 mg/mL and 0.80 mg/mL were selected to ensure both drug concentration and normal cell growth. Cells were divided into three groups: vehicle control group (10% FBS-DMEM, Veh), low-dose DZF group (0.4 mg/mL, 10% FBS-DMEM, DZF-L), and high-dose DZF group (0.8 mg/mL, 10% FBS-DMEM, DZF-H), with 2 d intervention.

Chemicals and materials used in cell culture: High glucose Dulbecco’s modified Eagle’s medium (DMEM), pancreatin, penicillin streptomycin (Gibco, United States); Newborn calf serum (NBCS), Premium grade fetal bovine serum (FBS) (Every Green, Zhejiang Tianhang Biotechnology Co., LTD, China); Insulin (Solarbio, China); 3-Isobutyl-1-methylxanthine (IBXM), Dexamethasone (DEX) (Sigma, United States); CCK8 kit (Dojindo Laboratories, Japan); TG assay kit (Nanjing Jiancheng Bioengineering Institute, China); BCA kit (Abcam, United States).

#### 2.3.2 Inhibitor intervention

H-89 dihydrochloride (Cat. No: HY-15979A, MCE, United States) was selected as a PKA inhibitor. Formulation methods: 1 mg H-89 dihydrochloride powder was dissolved in ultra-pure water, and an ultrasonic shock machine was used to promote dissolution at 80°C until the powder was completely dissolved. H-89 dihydrochloride mother liquor was eventually formulated with a concentration of 1 mM. The mother liquor was packed in EP tubes and frozen in a −80°C refrigerator away from light. When used, it was melted and 10% FBS-DMEM was added to prepare the corresponding concentration. The cell culture process is the same as before. By day 10 of cell differentiation, about 90% of 3T3-L1 cells differentiated into mature adipocytes. The cells on each culture plate were divided into four groups, namely, the vehicle control group (10% FBS-DMEM, Veh), DZF group (0.8 mg/mL DZF, 10% FBS-DMEM, DZF), PKA inhibitor group (30 μM H-89 dihydrochloride, 10% FBS-DMEM, H-89), DZF + PKA inhibitor group (0.8 mg/mL DZF+30 μM H-89 dihydrochloride, 10% FBS-DMEM, DZF + H-89).

### 2.4 Body length, abdominal circumference and Lee’s index

Mice were placed supine after anesthesia, and a tape measure was used as a tool, accurate to 0.01 cm. Body length: the distance from the tip of the nose to the anus; Abdominal circumference: the circumference of the abdomen at the midpoint of the vertical distance between the xiphoid process and the hind limbs. Lee’s index calculation formula: Lee’s index = [body weight(g)*10^3^/body length (cm)]^1/3^

### 2.5 Fasting blood glucose (FBG), serum lipids, and intracellular triglycerides (TG) measurement

The FBG of mice’s tail venous was measured by an automatic glucose meter (ACCU-CHEK Performa, Roche, Germany) After fasting for 6 h at the same time every week. The serum TG, total cholesterol (TC), high-density lipoprotein cholesterol (HDL-C), and low-density lipoprotein cholesterol (LDL-C) levels were assessed by an automatic biochemical analyzer in Guang’anmen Hospital. The 3T3-L1 cytosol was collected and operated according to the instructions of the TG assay kit. The TG content was calculated by combining the cellular protein concentrations of each group. The corresponding protein concentration was detected using the BCA kit.

### 2.6 Tissue and cell morphological analysis

Fresh adipose tissues were taken from the subscapularis, inguinal, perirenal, and epididymis of mice, cut into small pieces of about 1 cm × 0.5 cm × 1 cm, and made into paraffin sections. The morphological changes of adipocytes were observed by H&E staining. After inguinal white adipose tissue (iWAT) was fixed, dehydrated, dried, and treated for electrical conductivity, the mitochondria inside the adipocytes were observed under transmission electron microscopy (Hitachi, Japan) and photographed. 3T3-L1 cells were fixed with 10% formaldehyde fixative at room temperature for 1 h. After washing with PBS three times, lipid droplets were stained with BODIPY493/503 (Solarbio, China) working solution at a concentration of 2 μg/mL for 20 min at room temperature. The nuclei were stained by adding DAPI (Solarbio, China) for 30 min. The lipid droplets and nuclei staining were observed under a fluorescence microscope (Nikon, Japan) and photographed separately. Prepare 20 nM Mito-Tracker Green (Beyotime, China) working solution and preheat it in a 37°C water bath. Incubate the working solution with the cells for 45 min to stain the mitochondria. Remove the staining solution and add 37°C pre-warmed PBS. Observe the mitochondrial staining under a fluorescence microscope and take pictures.

### 2.7 Reverse transcription-quantitative PCR (RT-qPCR)

Mice iWAT total RNA was extracted using the RNeasy Lipid Tissue Mini Kit (QIAGEN, United States), and 3T3-L1 cell total RNA was extracted using Trizol (Invitrogen, United States). RNA concentrations were measured by a spectrophotometer (Nanodrop 2000c; Thermo Fisher, United States). cDNA synthesis was performed using SuperScript IV VILO Premix (Invitrogen, United States). qPCR was completed using SYBR Green Master Mix (ABI, United States) in 7500 Fast real-time fluorescence quantitative PCR system (ABI, United States). The relative expressed levels of the target genes were calculated according to a comparative method (2^-△△CT^) using β-Actin as the internal control. The corresponding primers of target genes were as follows (Table 2).

**TABLE 2:** Sequence of primers used for RT-qPCR.

Name	Sequence (5′-3′)	
UCP1	Forward primer	TCA​GCC​GGC​TTA​ATG​ACT​GG
	Reverse primer	TGA​TCC​CAT​GCA​GAT​GGC​TC
PGC-1α	Forward primer	GCT​GTG​TGT​CAG​AGT​GGA​TTG
	Reverse primer	CGC​AGG​CTC​ATT​GTT​GTA​CT
PKA	Forward primer	TCT​CTT​CCT​GTT​CCC​ACC​CT
	Reverse primer	CAG​GGC​ACT​AGC​ATT​ACG​GT
β-actin	Forward primer	CACTGTCGAGTCGCGTCC
	Reverse primer	TCA​TCC​ATG​GCG​AAC​TGG​TG

### 2.8 Western blotting

3T3-L1 cells were lysed on ice for 30 min using a high-potency RIPA tissue/cell lysis solution (Solarbio, China). The cell lysate was centrifuged at 4°C at 14000 g for 15 min, and the protein supernatant was obtained. Protein concentrations were determined using a BCA kit (Abcam, United States). The proteins were electrophoretically separated and transferred to PVDF membranes by sodium dodecyl sulfate (SDS)-polyacrylamide gel. Then, the PVDF membrane was sealed with 5% skim milk for 1 h and incubated with the corresponding primary antibody (Table 3) overnight at 4°C. β-Actin was used as the internal reference protein. The membrane was then incubated at room temperature with a secondary antibody (Goat Anti-Rabbit IgG, HRP Conjugated, Cwbio, 1:5000) conjugated to Horseradish peroxidase (HRP) for 1 h. ECL hypersensitive luminescence solution (Applygen, China) was used to detect protein signals, and the membrane was put into the Gel imaging System (Molecular Imager Gel Doc XR + system, Bio-Rad, United States). The images were obtained using Image Lab software. ImageJ software was used to analyze the image and get the corresponding gray value.

**TABLE 3:** Primary antibodies used for Western blotting.

Antibodies	Catalog no.	Company	Dilution
Anti-PKA	NT 06-903	Sigma (United States)	1:1000
Anti-CREB1	12208-1-AP	Proteintech (United States)	1:1000
Anti-Phospho-CREB1	28792-1-AP	Proteintech (United States)	1:1000
Anti-SREBP-1	14088-1-AP	Proteintech (United States)	1:1000
Anti-UCP1	U6382	Sigma (United States)	1:1000
Anti-PGC-1α	66369-lg	Proteintech (United States)	1:1000
Anti-β-Actin	#4970	Cell Signaling Technology (United States)	1:1000

### 2.9 Statistical analysis

Data were expressed as mean ± standard deviation (SD). Statistical analyses were performed using GraphPad Prism Version 9. Unpaired *t*-test and One-way ANOVA were used for continuous variables. Differences were considered statistically significant at *p* < 0.05.

## 3 Results

### 3.1 *In vivo* experiments

#### 3.1.1 DZF improves HDF-induced obesity and abnormal glucose and lipid metabolism in C57BL/6J mice

**FIGURE 2:**
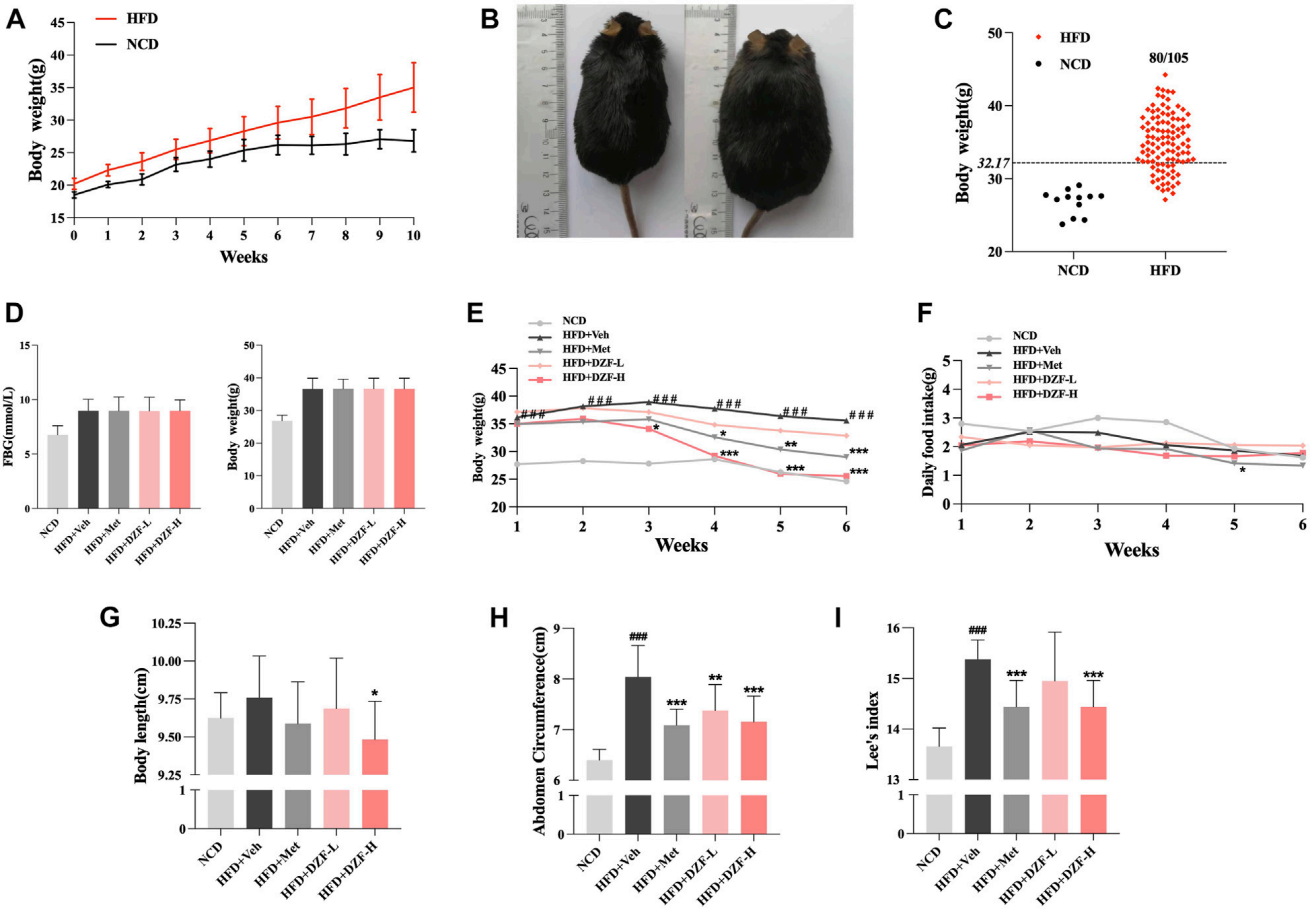
DZF improves HDF-induced obesity in C57BL/6J mice. **(A)** Body weight changes in diet-induced obese (DIO) C57BL/6J mice. Note: NCD is normal control diet group, *n* = 12; HFD is high-fat diets group, *n* = 105. Data are presented as the mean ± standard deviation (SD). ###*p* < 0.001 vs NCD. **(B)** NCD C57BL/6J mice (left) and HFD C57BL/6J mice at the 10th week of modeling. **(C)** Rate of model success in the DIO C57BL/6J mouse model. Note: The criterion for modeling was body weight >32.17 g. The number of mice in the HFD group that met the modeling criteria was 80/105. **(D)** FBG and body weight at 0 week. Changes in body weight **(E)** and daily food intake **(F)** of mice in each group within 6 weeks of the intervention. Body length**(G)**, abdominal circumference **(H)** and Lee’s index **(I)** after 6-week intervention. Note: NCD is normal control diet group, *n* = 12; HFD + Veh is vehicle control group, *n* = 12; HFD + Met is metformin control group, *n* = 12; HFD + DZF-L is DZF low dose group, *n* = 12; HFD + DZF-H is DZF high dose group, *n* = 12; Data are presented as the mean ± SD. ###*p* < 0.001 vs NCD; *p < 0.05, **p < 0.01, ***p < 0.001 vs HFD + Veh.

From the 2nd week of modeling, HFD group gained significantly more body weight than NCD group (*p* < 0.001, Figure 1A). In the 10th week of modeling, the HFD group was significantly fatter than the NCD group (Figure 2B), with 80 mice meeting the modeling standard, and the modeling rate was 76.19% (Figure 2C). HFD group was evenly divided into four groups according to FBG and body weight. At the 0 week after grouping, the FBG and body weight of obese mice in the four groups had no difference between groups (Figure 1D). During the intervention, body weight in HFD + DZF-H and HFD + Met groups decreased constantly compared with HFD + Veh groups. After the 3rd week, the body weight of HFD + DZF-H group was significantly lower than that of HFD + Veh group (*p* < 0.001), and the body weight of HFD + DZF-L group showed a decreasing trend (Figure 1E). From the 4th week, the daily food intakes of the two HFD + DZF groups and HFD + Veh group were gradually close. The daily feed intake in the HFD + Met group was consistently lower than that in the HFD + Veh group, with a significant difference at the 5th week (*p* < 0.05) and tended to be consistent at the end of intervention (Figure Figure1F). After 6 weeks of intervention, HFD + Veh group showed no significant increase in body length compared with NCD group. Compared with HFD + Veh, body length in HFD + DZF-H group was significantly decreased (*p* < 0.05), while that in DZF-L and Met groups was not significantly decreased (Figure 2G). Compared with NCD group, abdomen circumference and Lee’s index of HFD + Veh group were significantly increased (*p* < 0.001), while abdomen circumference and Lee’s index of HFD + DZF-H and HFD + Met group were significantly decreased (*p* < 0.001) (Figures 1H,I).

**FIGURE 3:**
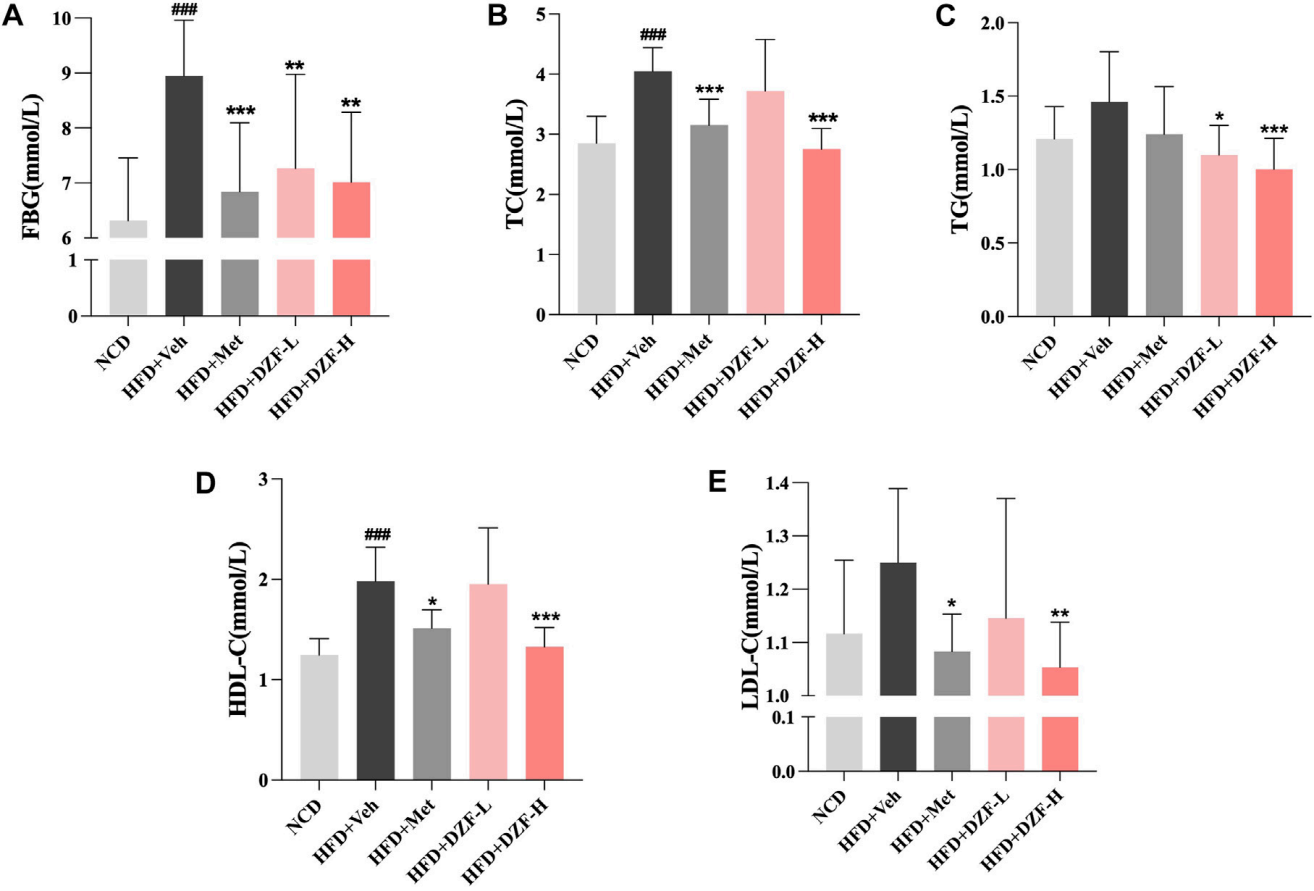
DZF improves abnormal glucose and lipid metabolism in DIO mice. **(A)** Fasting blood glucose (FBG), **(B)** Serum total cholesterol (TC), **(C)** Serum triglycerides (TG), **(D)** Serum high-density lipoprotein cholesterol (HDL-C), **(E)** Serum low-density lipoprotein cholesterol (LDL-C) at the 6 week of intervention. Note: **(A–E)** *n* = 12. Data are presented as the mean ± SD. ###*p* < 0.001 vs NCD; *p < 0.05, **p < 0.01, ***p < 0.001 vs HFD + Veh.

Compared with the HFD + Veh group, FBG was significantly reduced in HFD + DZF-L and HFD + Met group (*p* < 0.01 or *p* < 0.001, Figure 3A). Compared with HFD + Veh group, TC, TG, HDL-C and LDL-C in HFD + DZF-H group were significantly decreased (*p* < 0.01 or *p* < 0.001), and TG in HFD + DZF-L group was significantly decreased (*p* < 0.05). TC, HDL-C, and LDL-C were significantly reduced in the HFD + Met group (*p* < 0.05 or *p* < 0.001) (Figures 3B–E).

#### 3.1.2 DZF promotes iWAT browning and activates PKA pathway in DIO C57BL/6J mice

**FIGURE 4:**
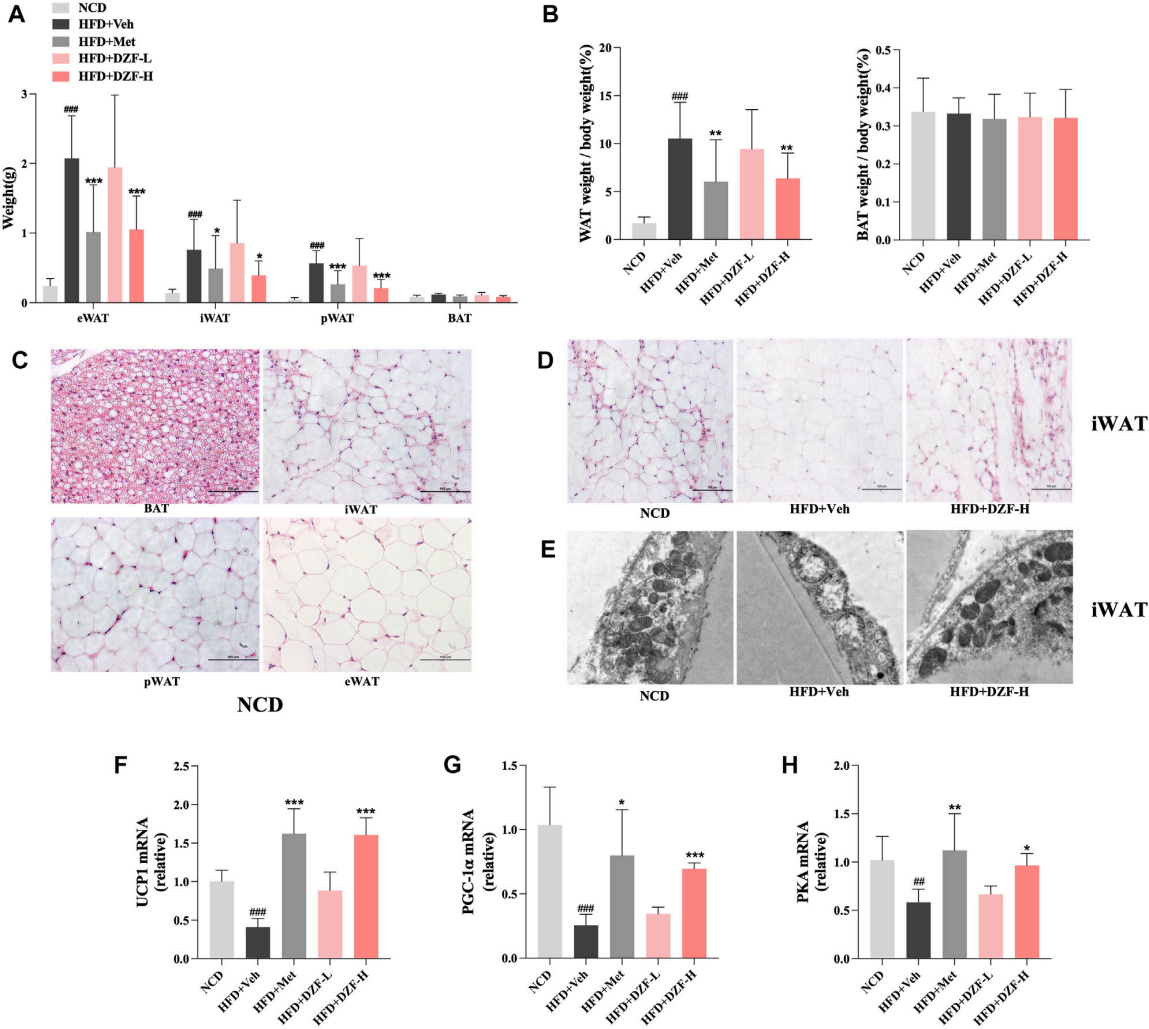
DZF promotes browning and activates PKA pathway in iWAT of DIO mice. **(A)** Weight of epididymal white adipose tissue (eWAT), inguinal white adipose tissue (iWAT), perirenal white adipose tissue (pWAT) and brown adipose tissue (BAT). **(B)** WAT weight/body weight and BAT weight/body weight of each group. **(C)** HE-staining of adipose tissue in NCD group (×200). BAT at top left, iWAT at top right, pWAT at bottom left, eWAT at bottom right. **(D)** HE-staining of iWAT; NCD (left), HFD + Veh (middle), HFD + DZF-H (right) (×200). **(E)** Mitochondrial transmission electron microscopy results of iWAT; NCD (left), HFD + Veh (middle), HFD + DZF-H (right) (×7000). The gene expression of UCP1 **(F)**, PGC-1α **(G)**, and PKA **(H)** of iWAT. Note: **(A and B)** *n* = 12, **(F–H)** *n* = 6. Data are presented as the mean ± SD. ###*p* < 0.001 vs NCD; *p < 0.05, **p < 0.01, ***p < 0.001 vs HFD + Veh.

Adipose tissue at different locations was weighed, and its ratio to body weight was calculated. Compared with HFD + Veh group, epididymal white adipose tissue (eWAT), inguinal white adipose tissue (iWAT), and perirenal white adipose tissue (pWAT) weight in HFD + DZF-H and HFD + Met groups were significantly reduced (*p* < 0.05 or *p* < 0.001, Figure 4A). Compared with HFD + Veh group, WAT weight/body weight in HFD + DZF-H group and HFD + Met group were significantly decreased (*p* < 0.01, Figure 4B). There were no significant differences in BAT weight and BAT weight/body weight among all groups (*p* > 0.05, Figures 4A,B). HE-staining of four different adipose tissue slices in NCD group was observed. Some cells with BAT characteristics were observed in iWAT cells with irregular morphology and smaller volume than eWAT and pWAT cells (Figure 4C), suggesting that iWAT has more potential for Browning than eWAT and pWAT cells. To further clarify the effect of DZF on the Browning of iWAT cells, the morphology of iWAT cells stained by HE in each group was observed. Compared with HFD + Veh group, fat cells in HFD + DZF-H group were significantly reduced in volume, irregular in shape, and more obvious in Browning (Figure 4D). Transmission electron microscopy was further used to observe mitochondrial morphology in iWAT. Mitochondria in adipocytes of HFD + Veh group showed vacuole-like changes. HFD + DZF-H increased the number of mitochondria and made their structure clearer (Figure 4E). The mRNA expressions of brown-labeled UCP1 and PGC-1α, as well as the mRNA expressions of PKA were detected in iWAT of each group. Compared with HFD + Veh group, the three mRNA expressions in iWAT of HFD + Met group and HFD + DZF-H group were increased to varying degrees (*p* < 0.05 or *p* < 0.01 or *p* < 0.001, Figures 4F–H).

### 3.2 *In vitro* experiments

#### 3.2.1 DZF inhibits lipid accumulation and adipogenesis in 3T3-L1 cells

**FIGURE 5:**
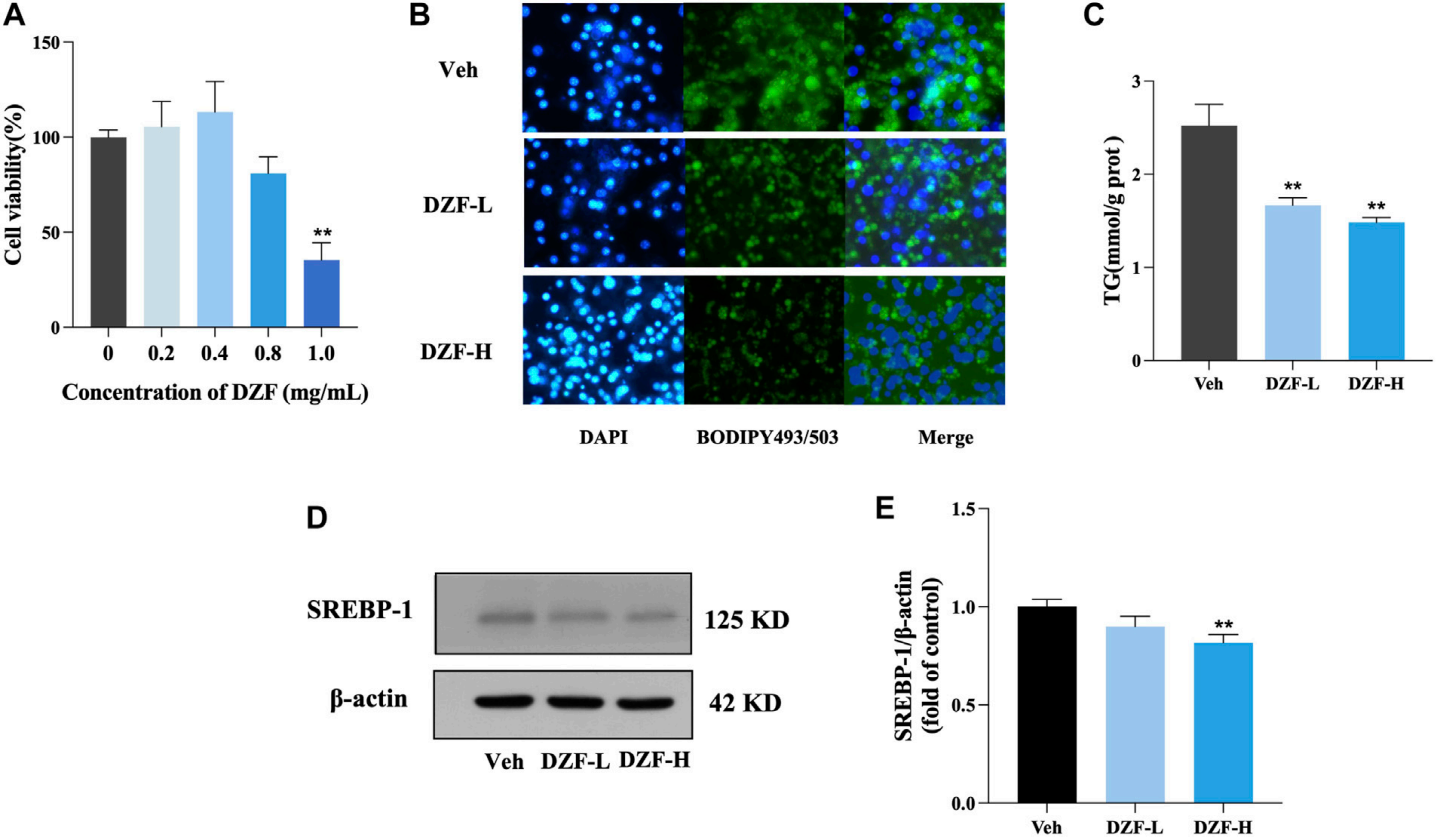
DZF inhibits lipid accumulation and adipogenesis in 3T3-L1 cells. **(A)** Cell Counting Kit-8 (CCK8) was used to detect the effect of DZF in different concentrations on the activity of 3T3-L1 adipocytes. **(B)** BODIPY493/503 staining images of lipid droplet in 3T3-L1 adipocytes (green for lipid droplet, blue for nucleus, ×100). **(C)** Intracellular TG content. **(D and E)** Western blotting of SREBP-1 protein expression in 3T3-L1 cells. Note: Veh is vehicle control group, DZF-L is low-dose DZF group, DZF-H is high-dose DZF group, **(A,C and E)** *n* = 3. Data are presented as the mean ± SD. ###*p* < 0.001 vs NCD; *p < 0.05, **p < 0.01 vs Veh.

The results of CCK8 showed no significant change in cell survival rate when DZF concentration was 0.4 mg/mL and 0.8 mg/mL, indicating that DZF had no significant enhancement or inhibition effect on cell proliferation at this concentration. Therefore, 0.4 mg/mL and 0.8 mg/mL were used as DZF concentrations for subsequent *in vitro* experiments (Figure 5A). The morphology and number of lipid droplets were observed, and the effect of DZF on intracellular lipids was preliminarily evaluated. Compared with Veh group, the volume of lipid droplets in DZF-L and DZF-H groups decreased (Figure 5B). The intracellular TG content was further detected. Compared with Veh group, the intracellular TG content in DZF-L and DZF-H groups was significantly decreased (*p* < 0.01, Figure 5C). The expression of sterol regulatory element binding protein-1 (SREBP-1), which regulates lipid production, was detected. Compared with Veh group, the expression of SREBP-1 in DZF-H group was significantly decreased (*p* < 0.01, Figures 5D,E). These results indicated that DZF inhibited the accumulation and production of lipids in adipocytes.

#### 3.2.2 DZF promotes browning of 3T3-L1 adipocytes through activation of the PKA pathway

**FIGURE 6:**
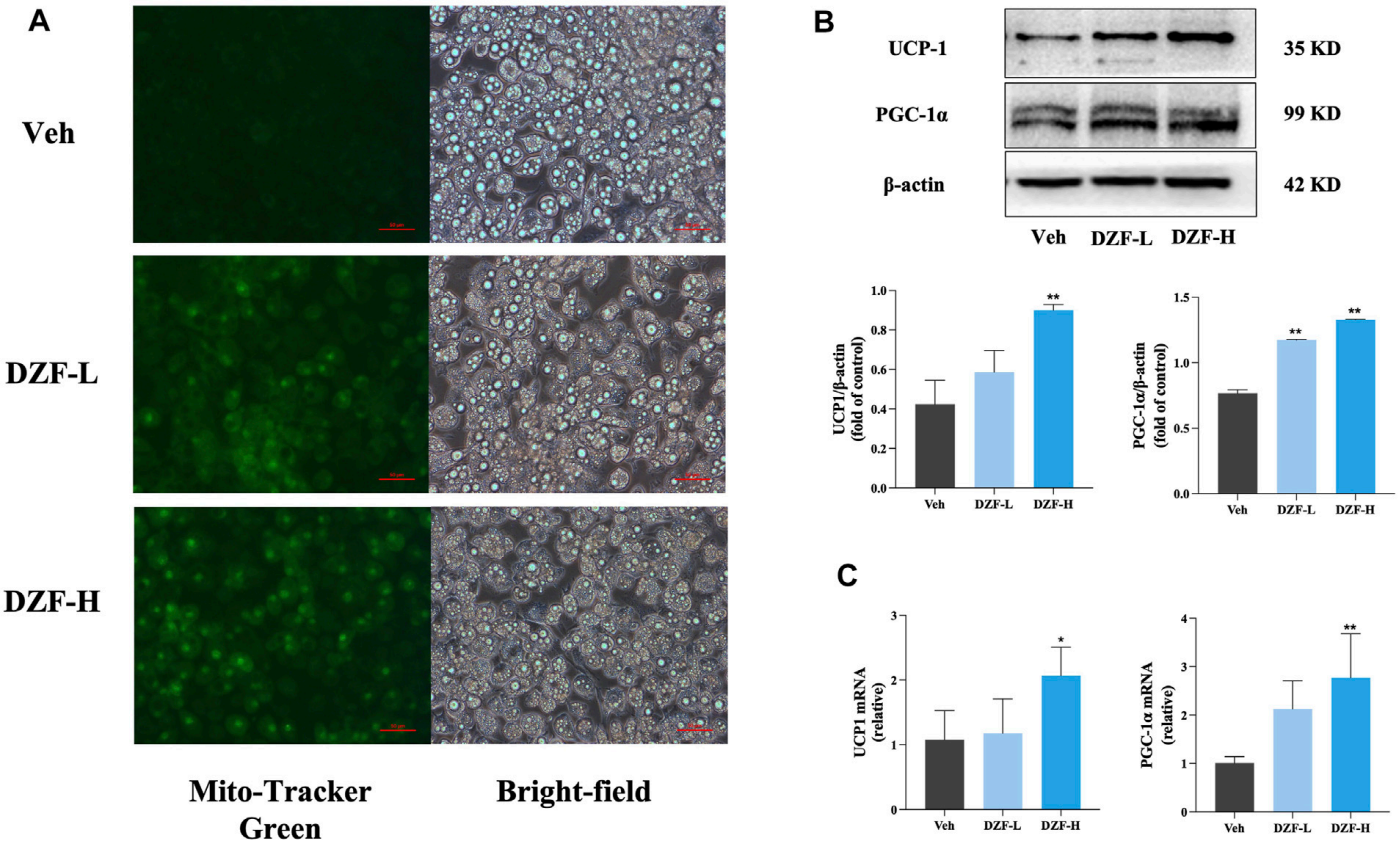
DZF promotes browning of 3T3-L1 adipocytes. **(A)** Dark-field (left) and bright-field (right) images of 3T3-L1 adipocytes were stained with mito-tracker Green to observe the number of mitochondria. Expression of UCP1, PGC-1α protein **(B)** and mRNA **(C)**. Note: **(B)** *n* = 3, **(C)** *n* = 4, data are presented as the mean ± SD.*p < 0.05, **p < 0.01 vs Veh.

Mito-Tracker Green was used to label mitochondria specifically. The results showed that compared with Veh group, the number of mitochondria in adipocytes in DZF-L and DZF-H groups was significantly increased (Figure 6A). Western blot was used to detect the expression of UCP1 and PGC-1α, the key markers of Browning, in adipocytes of each group. The results showed that compared with Veh group, the protein expressions of UCP1 and PGC-1α in DZF-L and DZF-H groups were significantly increased (*p* < 0.01), indicating that DZF could promote the Browning of WAT cells (Figure 6B). Similarly, both UCP1 and PGC-1α mRNA expressions were upregulated by DZF intervention (*p* < 0.05 or *p* < 0.01, Figure 6C).

**FIGURE 7:**
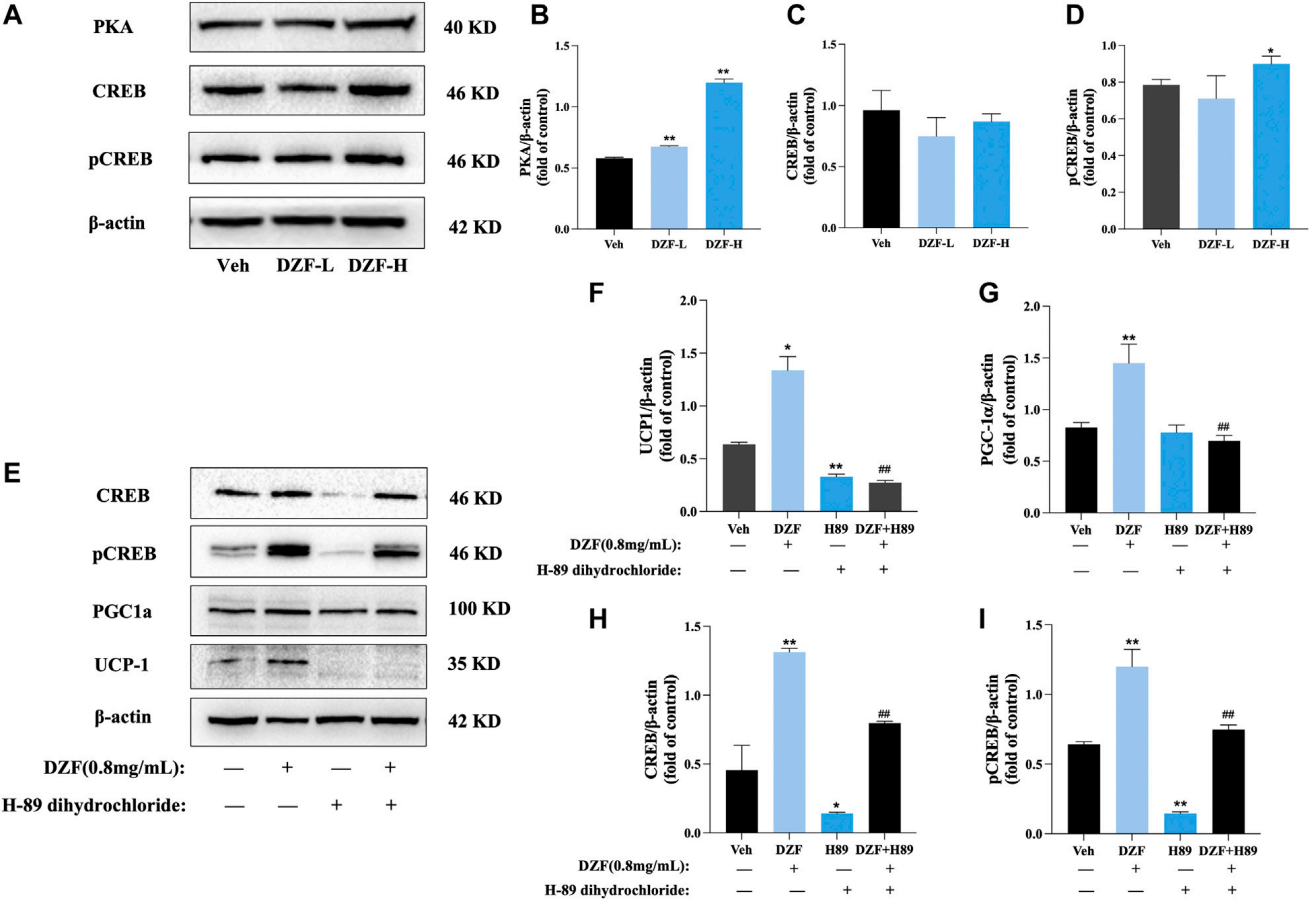
DZF promotes browning of 3T3-L1 adipocytes through activation of the PKA pathway. **(A–D)** Expression of PKA, CREB and pCREB proteins. Note: *n* = 3, data are presented as the mean ± SD, *p < 0.05 and**p < 0.01, vs Veh group. **(E–I)** Expression of PGC-1α, UCP1, CREB and pCREB protein after PKA inhibition. Note: Veh is vehicle control group (10% FBS-DMEM, Veh), DZF is DZF group (0.8 mg/mL), H-89 is PKA inhibitor group (30 μM H-89 dihydrochloride), DZF + H-89 is DZF + PKA inhibitor group (0.8 mg/mL DZF+30 μM H-89 dihydrochloride); *n* = 3, data are presented as the mean ± SD, *p < 0.05, **p < 0.01 vs Veh group; #*p* < 0.05, ##*p* < 0.01 vs DZF group.

To explore whether the mechanism of WAT Browning promoted by DZF is related to the PKA pathway, the protein expressions of PKA, cyclic adenosine monophosphate (cAMP) response-element binding protein (CREB) and phosphorylated CREB (pCREB) in each group were preliminarily detected. The results showed that compared with Veh group, the protein expressions of PKA and pCREB were significantly increased after DZF intervention (*p* < 0.01 or *p* < 0.05), while the protein expression of CREB was not significantly changed (*p* > 0.05), indicating that DZF could activate the PKA-Creb pathway (Figures 7A–D). The PKA inhibitor H-89 dihydrochloride was further used to inhibit the effect of PKA in cells. The group was re-configured to include DZF group, H-89 group, DZF + H-89 group, and solvent control group. The results showed that compared with DZF group, the protein expressions of PGC-1α and UCP1 in DZF + H-89 group were significantly decreased (*p* < 0.01), and the expressions of CREB and pCREB were also significantly decreased. These results indicated that inhibition of PKA weakened the Browning effect of DZF on adipocytes (Figures 7E–I).

## 4 Discussion

Adipose tissue mainly consists of WAT and BAT. WAT content is the most abundant, distributed primarily on subcutaneous tissue and around viscera. WAT usually contains a single large lipid droplet and a few mitochondria. The primary function is to store excess energy in the form of TG in lipid droplets. Hyperplasia and hypertrophy of WAT are typical pathological manifestations of obesity (Koenen et al., 2021; Sakers et al., 2022). BAT mainly exists in the neck and subscapular region, and its morphology is manifested as multiple tiny lipid droplets with minimal lipid storage capacity, but it contains abundant mitochondria. The primary function of BAT is heat generation through uncoupled respiration. Excess lipids are oxidized and decomposed to regulate the body’s energy metabolism balance (Richard et al., 2020).

iWAT belongs to subcutaneous fat, while eWAT and pWAT belong to visceral fat. In this study, eWAT increased significantly in DIO mice, indicating significant visceral obesity in mice, similar to abdominal obesity in humans. eWAT, iWAT, and pWAT were reduced considerably by DZF intervention, suggesting that DZF could improve visceral obesity in mice. Visceral obesity is more likely to cause abnormalities of glucose and lipid metabolism (Martel et al., 2017). FBG was significantly increased in DIO mice, indicating that obesity caused impaired glucose metabolism. It is well known that obesity and diabetes are closely related. In the obese population, the incidence of type 2 diabetes mellitus is higher (GBD 2019 Risk Factors Collaborators, 2020). In this study, DZF reduced FBG in DIO mice. Previous studies have shown that berberine, one of the signature components of DZF, can prevent the apoptosis of pancreatic β cells (Li et al., 2019) and promote insulin secretion by targeting the KCNH6 channel (Zhao et al., 2021). Therefore, DZF may have a certain clinical effect on obese patients with prediabetes (impaired fasting glucose). DZF also regulated lipid metabolism disorder by reducing serum TC, TG, and LDL-C levels in DIO mice. Several studies have shown that berberine, hesperidin, and lovastatin, the signature components of DZF, can regulate lipid metabolism and improve dyslipidemia (Shen et al., 2019; Yu et al., 2021; Banach et al., 2022). Dysregulation of lipid metabolism can lead the development of atherosclerosis and endothelial injury (Libby et al., 2019). Therefore, DZF may prevent cardiovascular and cerebrovascular diseases complicated by obesity in the clinic, which needs further study.

Beige adipocytes are a special kind of adipose tissue, whose morphology and function are similar to BAT, with multilocular lipid droplets and abundant mitochondria (Rui, 2017). Beige adipocytes are transformed from WAT stimulated by cold and chemicals, which can increase energy consumption and reduce WAT accumulation through heat generation, namely, WAT browning (Montanari et al., 2017). For adults, beige adipocytes have a stronger metabolic benefit than BAT because they are activated by multiple pathways and have higher utilization of fatty acids and glucose (Ikeda et al., 2018; Park et al., 2021). Browning is a meaningful way to regulate energy balance and improve metabolism, which helps to improve obesity and related metabolic abnormalities (Bartelt and Heeren, 2014; Kusminski et al., 2016). Therefore, we focused on whether DZF ameliorates obesity by inducing browning. Some of DZF’s active ingredients have been shown to promote browning. Rhizoma Coptidis, its active component berberine, enhanced the beige adipogenesis of 3T3-L1 cells through transcription-coupled post-transcriptional regulation (Lin et al., 2019). Nobiletin, the active component of *Citrus×aurantium* L., induced brown adipocyte-like phenotype and ameliorated stress in 3T3-L1 adipocytes (Lone et al., 2018).

The morphological characteristics of adipocytes correspond to their functions. White adipocytes contain a single-large lipid droplet with regular morphology. On the other hand, beige and brown adipocytes are small in size, contain multilocular lipid droplets, and have irregular shapes (Zwick et al., 2018). HE-staining showed that the volume of iWAT cells of DIO mice increased significantly, and single vacuoles of regular shape could be seen. High-dose DZF significantly reduced the volume of iWAT cells, and cells resembling BAT appeared, indicating that DZF could induce morphological browning of WAT. Previous studies have observed that subcutaneous fat in mice is more likely to induce browning than visceral fat (Zuriaga et al., 2017), which is consistent with the phenomenon observed in this study. Therefore, the degree of iWAT browning and its possible mechanism was focused on in subsequent *in vivo* experiments. *In vitro* experiments, DZF reduced the lipid storage of mature 3T3-L1 adipocytes, resulting in a decrease in the volume and a relative increase in the number of lipid droplets, showing the overall characteristics of beige adipocyte morphology, consistent with the results of HE-staining. In our previous study, the mRNA expressions of acetyl-CoA carboxylase (ACC) and fatty acid synthetase (FAS) were significantly downregulated after the intervention of 3T3-L1 adipocytes with DZF (Zhu et al., 2015a). These results indicated that DZF could reduce lipid deposition in adipocytes.

Browning activates beige adipocytes and increases energy consumption through mitochondria. Compared with WAT, activated beige adipocytes’ mitochondria are significantly larger and contain dense cristae (Lu, 2019). Mitochondria play a crucial role in maintaining energy homeostasis in adipose tissue. Mitochondria in adipocytes regulate adaptive thermogenesis, adipocyte differentiation, lipid homeostasis, oxidation capacity, insulin sensitivity, and other functions (Sustarsic et al., 2018; Lee et al., 2019; Mooli et al., 2020; Joffin et al., 2021). Obesity will decrease mitochondrial density and dysfunction, thus affecting the body’s metabolic balance and increasing the risk of metabolic diseases (Heinonen et al., 2020; Brestoff et al., 2021). *In vivo* experiment, the number of mitochondria in adipose cells of DIO mice decreased and showed vacuole-like changes. DZF increased the number and volume of mitochondria. The cristae in mitochondria were denser, and the structure was clear, similar to the morphology of activated beige adipocytes mitochondria. These results suggest that DZF promotes mitochondrial remodeling in hypertrophic WAT and may benefit mitochondrial function recovery. *In vitro* experiments also showed that DZF treatment increased the number of mitochondria in 3T3-L1 adipocytes. These results indicated that DZF promoted the browning of WAT mitochondria.

UCP1 and PGC-1α are often used to evaluate browning. UCP1 is a mitochondrial transmembrane protein. Activated UCP1 decoupling electron transport in the respiratory chain, thus preventing the production of ATP and dissipating energy in the form of heat (Zafrir, 2013), and is an essential marker for mediating non-shivering thermogenesis of BAT (Liu et al., 2019). Studies have shown that there are UCP1-independent thermogenesis pathways in adipose tissue, while subcutaneous fat is strongly UCP1-dependent (Bertholet et al., 2017; Cohen and Kajimura, 2021). Increased UCP1 expression in WAT can also improve glucose uptake and inflammation (Tews et al., 2019; Mills et al., 2022). PGC-1α is one of the specific markers of beige adipocytes and an essential regulator of mitochondrial biogenesis (Cheng et al., 2018; Kobayashi et al., 2021). As a transcriptional coactivator, PGC-1α induces the expression of the mitochondrial and thermogenic genes (Zhou et al., 2019). Studies have shown that high-calorie diet reduces the expression of UCP1 and PGC-1α in WAT (Fromme and Klingenspor, 2011). In this study, DZF upregulated the mRNA expression of UCP1 and PGC-1α in iWAT of DIO mice. Notably, DZF enhanced the protein and mRNA expression of UCP1 and PGC-1α in 3T3-L1 adipocytes, which was consistent with the results of animal experiments. These findings suggest that DZF can promote WAT browning through the UCP1-dependent pathway. As for whether the brown-inducing effect of DZF is achieved through the UCP1-independent pathway remains to be further explored.

**FIGURE 8:**
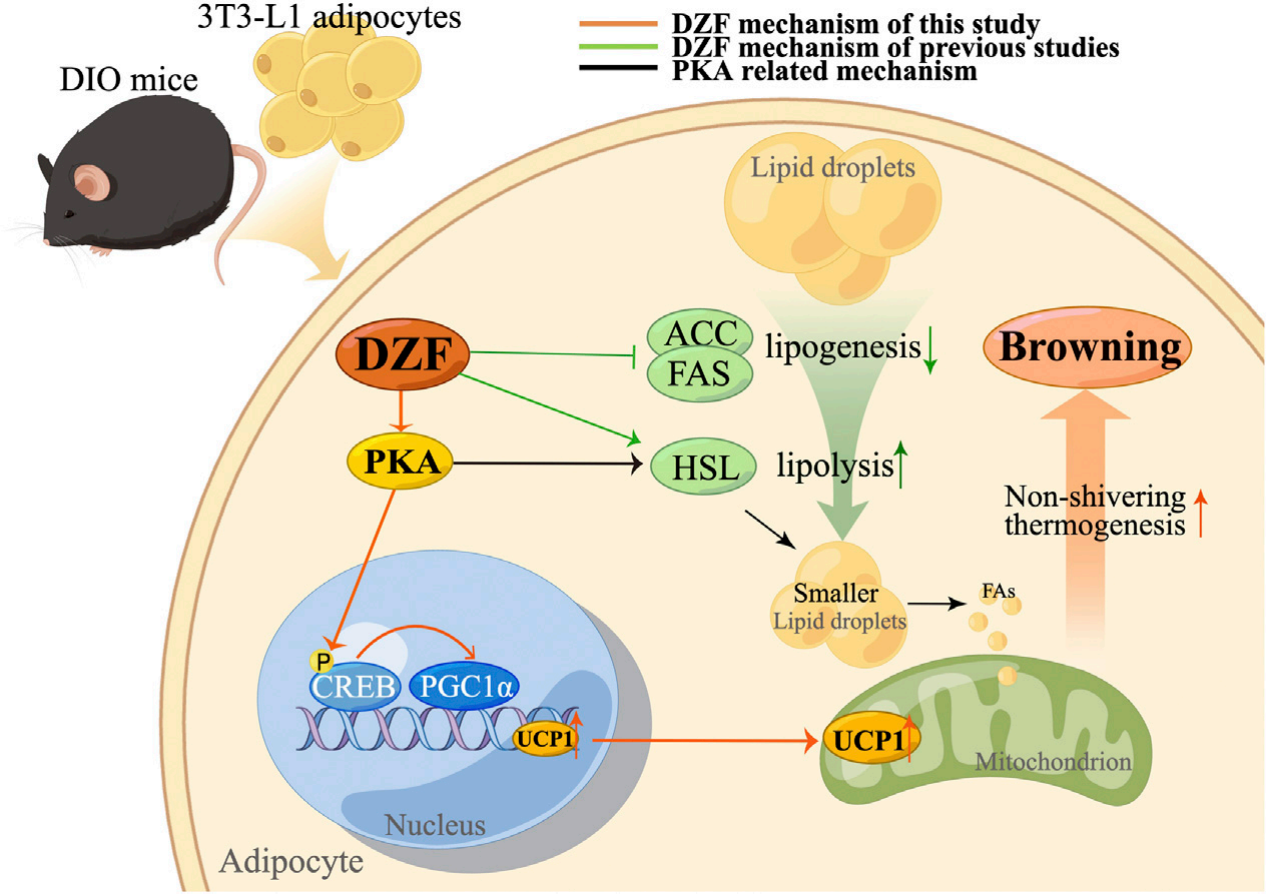
Mechanism of action (MOA) of DZF. The orange line represents the MOA of DZF confirmed in this study, the green line represents the MOA of DZF verified by our team in previous studies, and the black line represents the possible MOA of DZF related to the PKA pathway. In this study, DZF was used to intervene DIO mice and 3T3-L3 adipose cells, and it was found that DZF could improve the excessive lipid deposition in adipose cells, promote the expression of PKA, phosphorylate CREB, activate PGC-1α and thus promote the expression of UCP1, causing WAT browning. Previous studies have confirmed that DZF inhibits the expression of adipogenesis-related enzymes ACC, and FAS and promotes the expression of key lipolysis enzyme HSL, which may also be related to the activation of PKA pathway. Note: DIO, diet-induced obese; DZF, Dai-Zong-Fang; ACC, acetyl-CoA carboxylase; FAS, fatty acid synthetase; HSL, hormone-sensitive lipase. (Pictrue by Figdraw).

The PKA signaling pathway is an essential regulator of adipocyte energy metabolism (London et al., 2014) and regulates thermogenesis by different pathways. CREB is a transcriptional enhancer that mainly responds to the PKA signaling pathway. Transforming stimulus signals of cellular molecules, such as pheromones and neurotransmitters, into various physiological activities in cells requires the mediation of cAMP, the second messenger in cells. PKA is a crucial target of cAMP. PKA activates p38 MAPK, which then enables phosphorylation and activation of transcription factor-2 (ATF-2) via cAMP in response to CREB to promote PGC-1α and UCP1 transcription. PKA also promotes lipolysis by activating hormone-sensitive lipase (HSL) (Petersen et al., 2008) and releases free fatty acid (FFA) to promote mitochondrial β-oxidation, thus generating acetyl-CoA and stimulating UCP1 expression (Dickson et al., 2016). Previous studies of our team have confirmed that DZF can increase HSL mRNA expression in 3T3-L1 adipocytes, which may be related to the activation of PKA pathway (Zhu et al., 2015b). In this study, DZF stimulated the upregulation of PKA levels in iWAT and increased the levels of PKA and pCREB proteins in 3T3-L1 cells, demonstrating the activation effect of DZF on PKA signaling pathway. To further determine whether the PKA pathway mediates DZF to promote WAT browning, H-89 dihydrochloride was used to treat mature 3T3-L1 adipose cells in this study. H-89 dihydrochloride is a PKA inhibitor to effectively inhibit the phosphorylation of PKA substrate CREB. The expression of brown-labeled proteins PGC-1α and UCP1 in DZF-treated cells was significantly reduced by H-89 dihydrochloride, suggesting that inhibiting PKA signaling pathway could reduce the DZF-induced WAT browning. Therefore, the PKA signaling pathway is one of the pathways that DZF regulates the browning of WAT. The possible mechanism of action of DZF is summarized as follows (Figure 8).

## 5 Conclusion

In summary, from different perspectives of morphological characteristics and molecular expression, the study demonstrated that DZF could induce the browning of white adipocytes *in vitro* and *in vivo* models, including DIO C57BL/6J mice and 3T3-L1 adipocytes. Browning was specifically manifested in the changes of morphological structure and molecular expression after DZF intervention, such as reduced lipid deposition, increased mitochondrial number and increased expression of Browning marker molecule (UCP1 and PGC-1α). The possible molecular mechanism of DZF’ browning is related to activating PKA pathway to promote UCP1 expression. By exploring the mechanism of DZF promoting browning, the pharmacologic connotation of DZF in treating obesity was further clarified. DZF has a certain curative effect on obesity and related abnormal glucose and lipid metabolism, which brings gospel to obesity patients.

## Data Availability

The original contributions presented in the study are included in the article/Supplementary Material, further inquiries can be directed to the corresponding authors.

## Abbreviations

BAT, brown adipose tissue; CAMP, cyclic adenosine monophosphate; CCK8, Cell Counting Kit-8; CREB, cAMP response-element binding protein; DEX, Dexamethasone; DIO, diet-induced obese; DMEM, Dulbecco’s modified Eagle’s medium; DZF, Dai-Zong-Fang; eWAT, epididymal white adipose tissue; FBS, fetal bovine serum; HDL-C, high-density lipoprotein cholesterol; HFD, high fat diet; HPLC, high-performance liquid chromatography; IBXM, Isobutyl-1-methyxanthine; iWAT, inguinal white adipose tissue; LDL-C, low-density lipoprotein cholesterol; MS, metabolic syndrome; T2DM, type 2 diabetes mellitus; NCD, normal control diet; NBCS, Newborn calf serum; pCREB, phosphorylated CREB; PGC-1*α*, Peroxisome proliferators-activated receptor *γ* coactivator-1 alpha; PKA, Protein kinase A; pWAT, perirenal white adipose tissue; TC, total cholesterol; TCM, Traditional Chinese medicine; TG, triglycerides; UCP1, Uncoupling protein 1; WAT, white adipose tissue.
