# Supplementary figures and images for: The Chinese herbal medicine Dai-Zong-Fang promotes browning of white adipocytes in vivo and in vitro by activating PKA pathway to ameliorate obesity
Source: Front Pharmacol. 2023 May 10;14:1176443. doi: 10.3389/fphar.2023.1176443 (PMC10211343; doi:10.3389/fphar.2023.1176443)

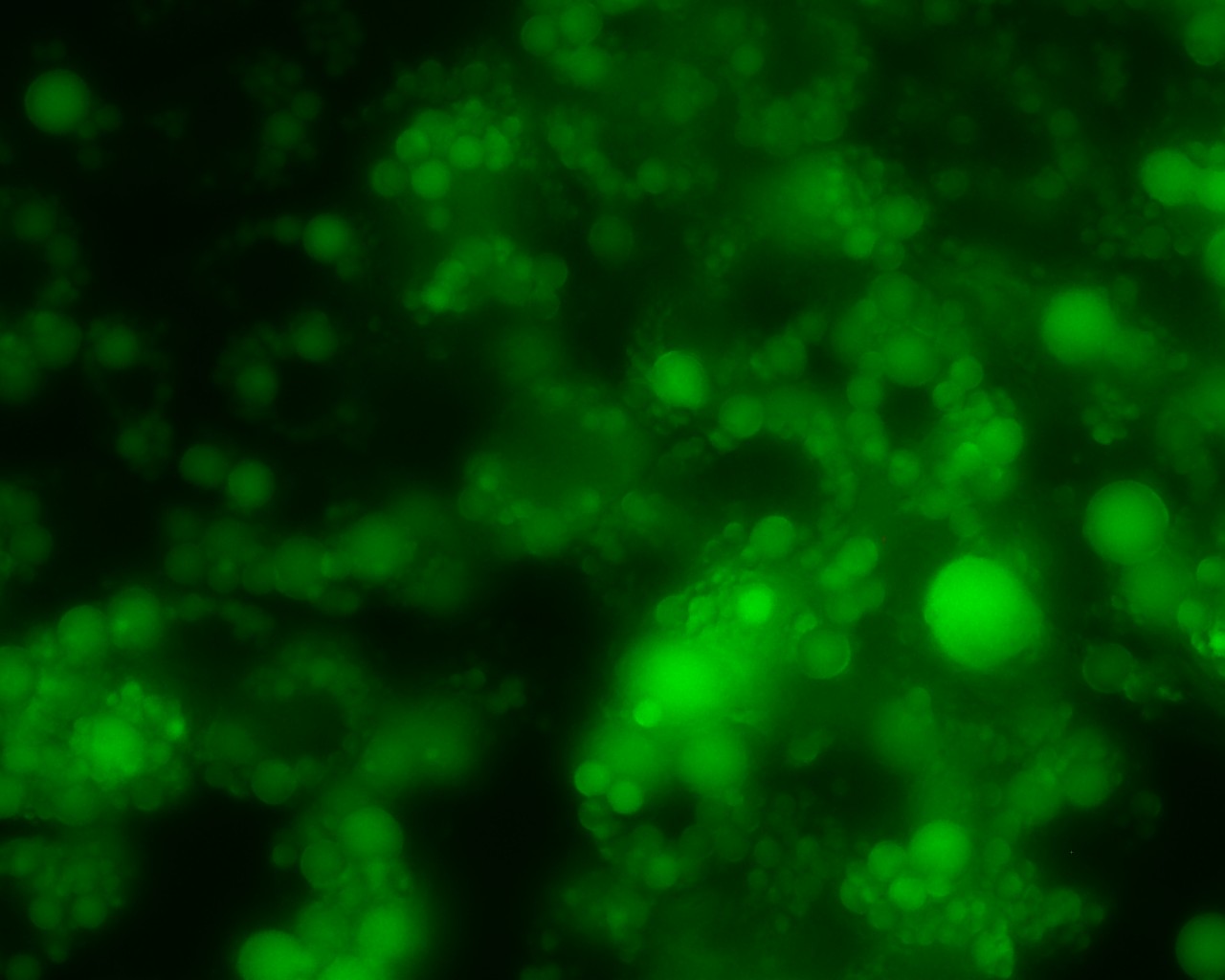

Supplement: Supplementary file 1 [file DataSheet3.ZIP › Original images-3T3-L1+BODIPY493:503/Veh-BODIPY.jpg]

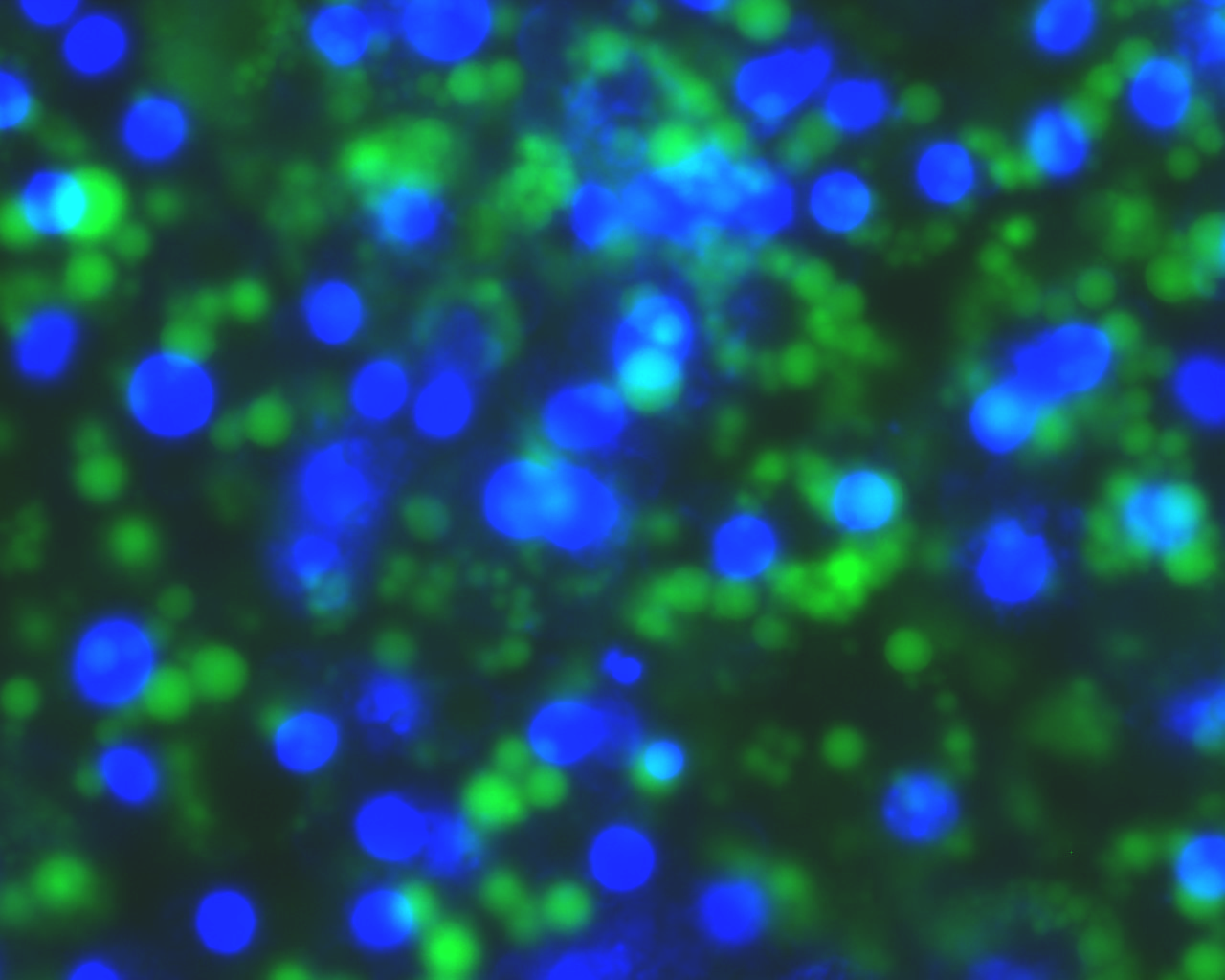

Supplement: Supplementary file 1 [file DataSheet3.ZIP › Original images-3T3-L1+BODIPY493:503/DZF-L-Merge.tif]

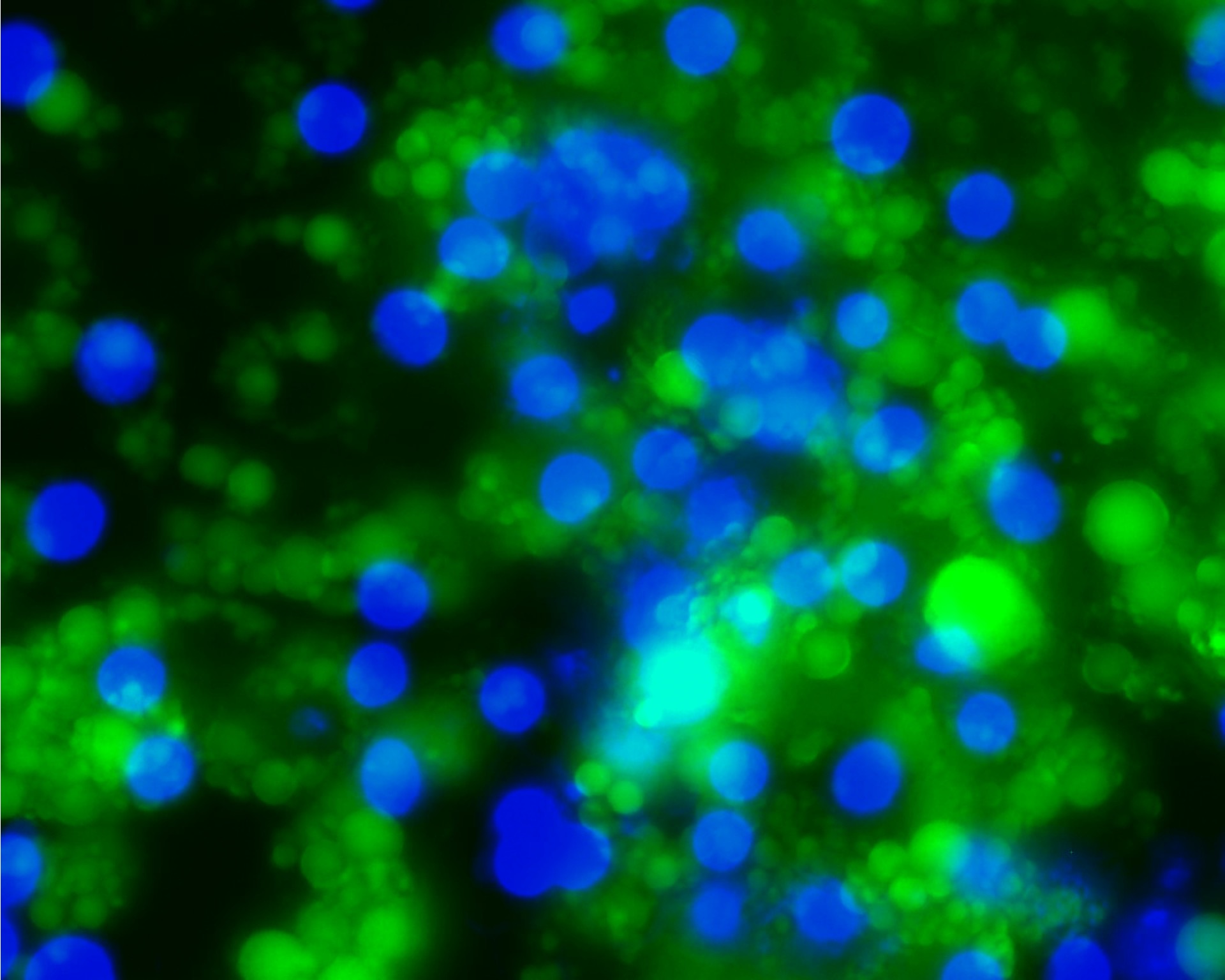

Supplement: Supplementary file 1 [file DataSheet3.ZIP › Original images-3T3-L1+BODIPY493:503/Veh-Merge.jpg]

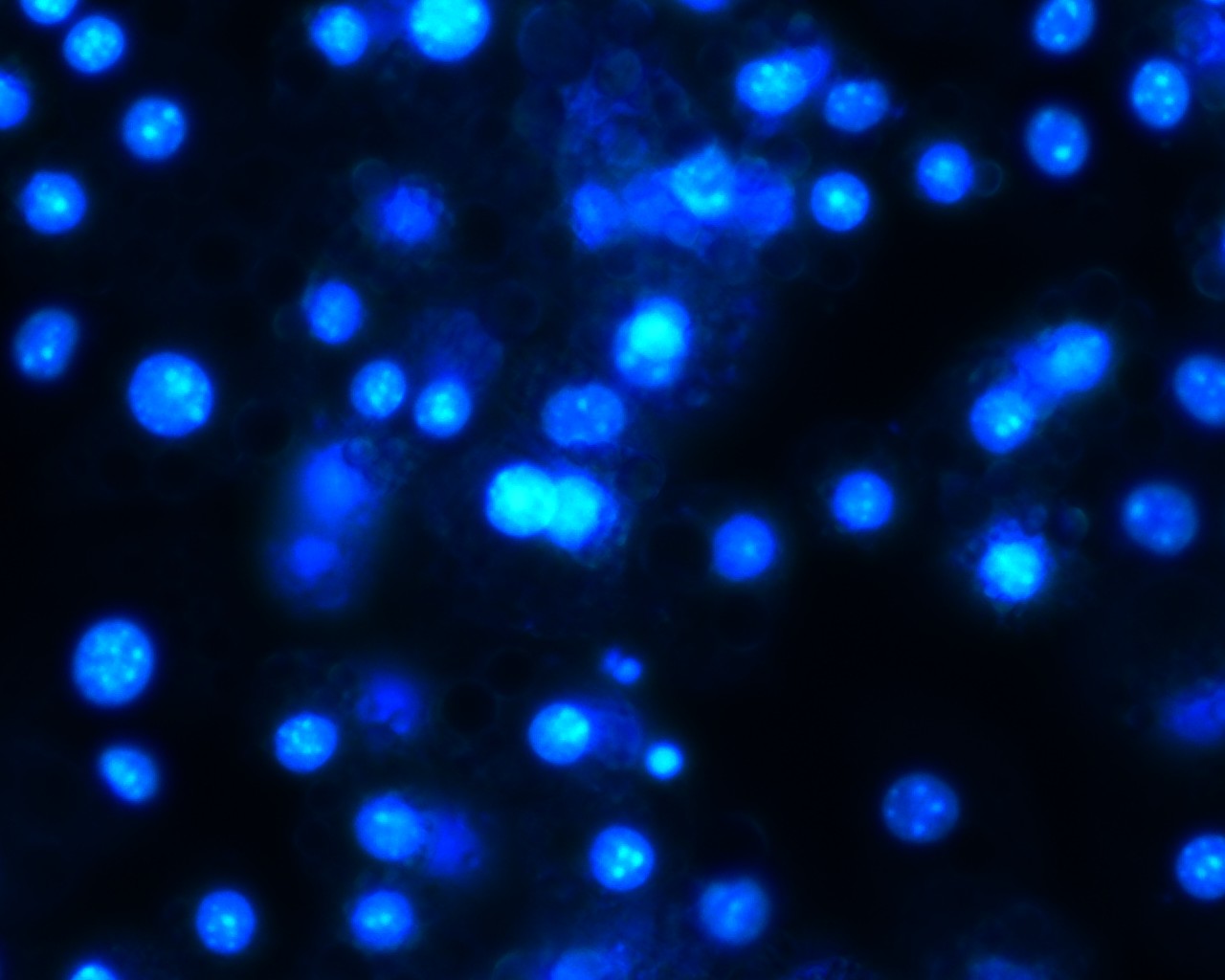

Supplement: Supplementary file 1 [file DataSheet3.ZIP › Original images-3T3-L1+BODIPY493:503/DZF-L-DAPI.jpg]

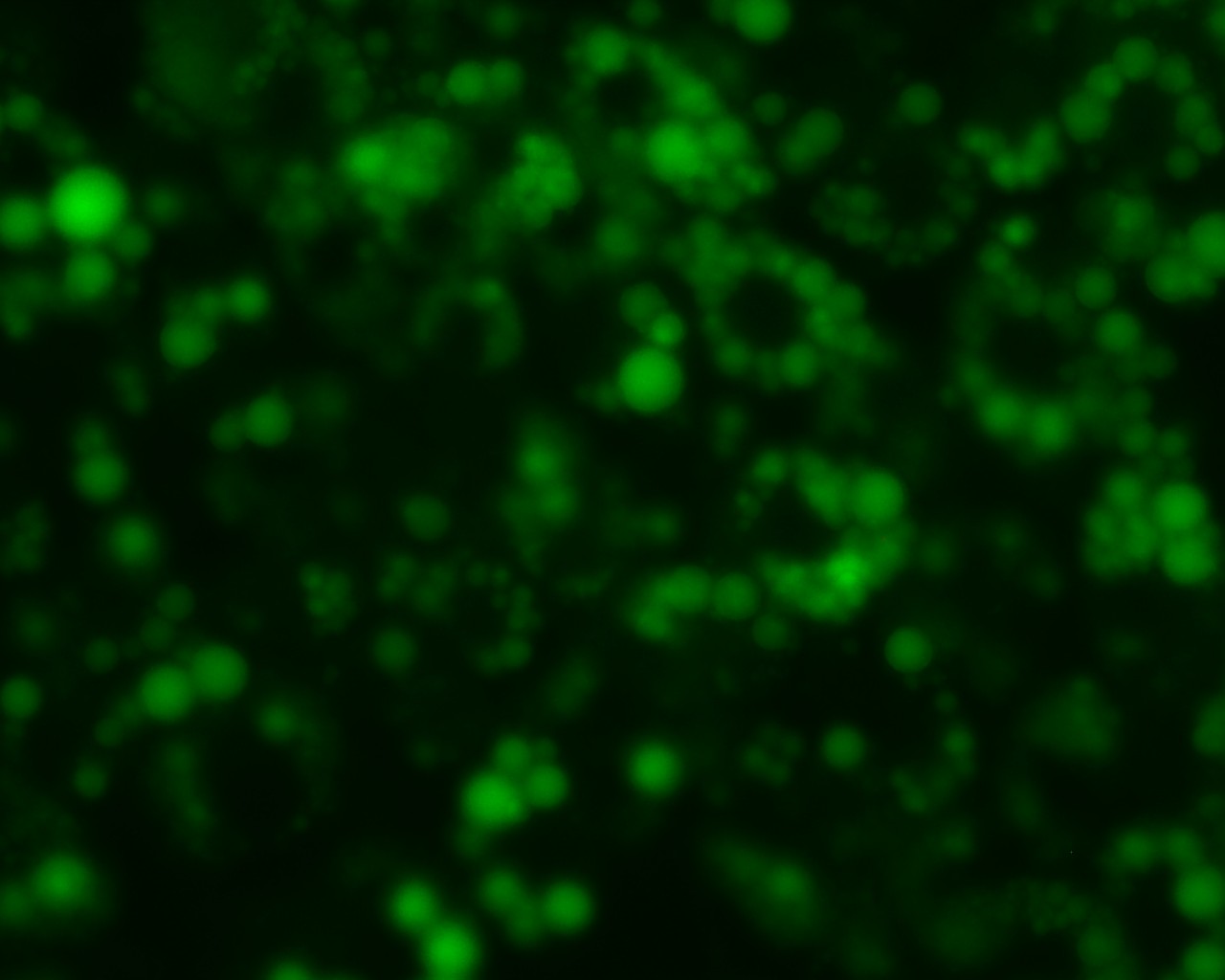

Supplement: Supplementary file 1 [file DataSheet3.ZIP › Original images-3T3-L1+BODIPY493:503/DZF-L-BODIPY.jpg]

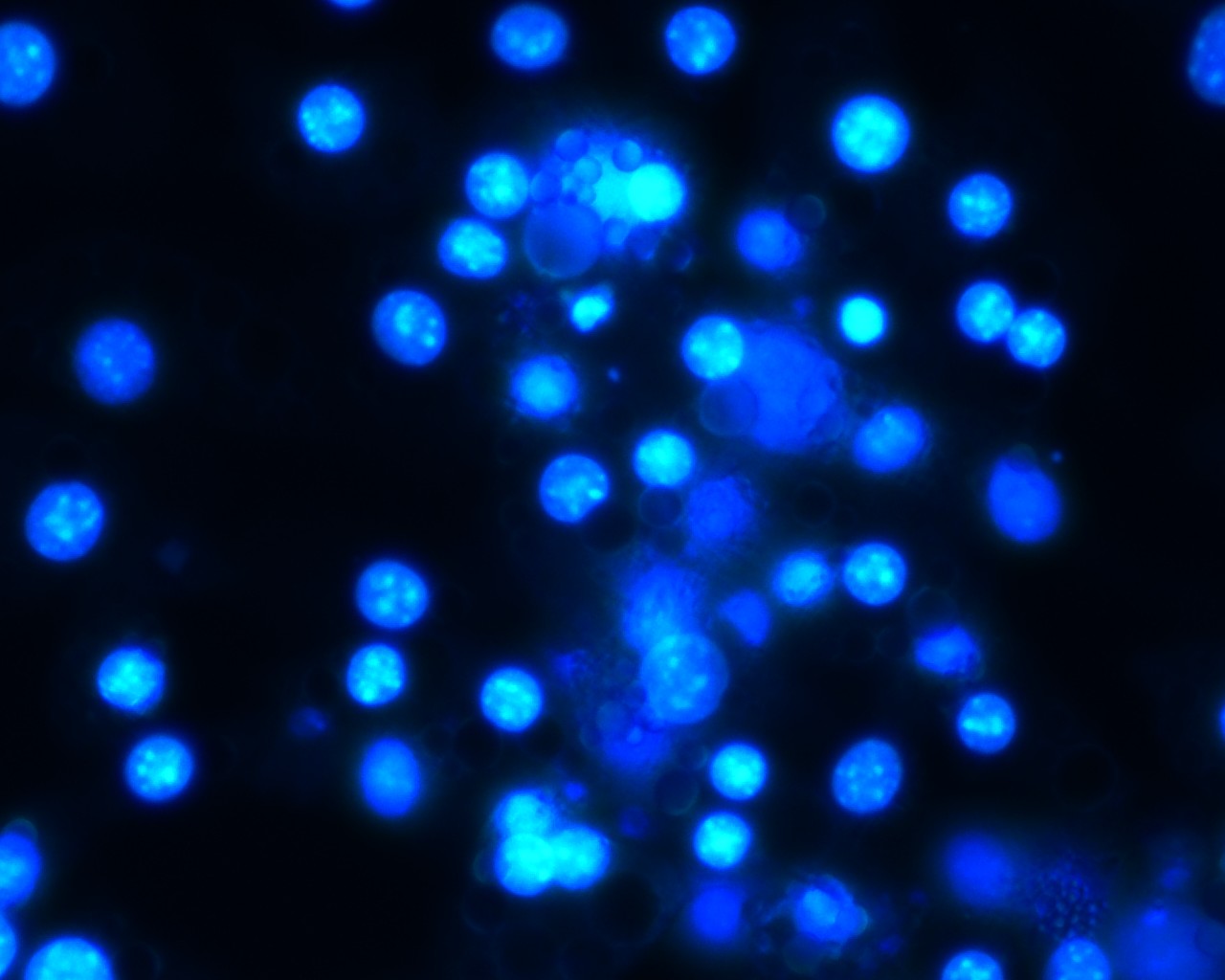

Supplement: Supplementary file 1 [file DataSheet3.ZIP › Original images-3T3-L1+BODIPY493:503/Veh-DAPI.jpg]

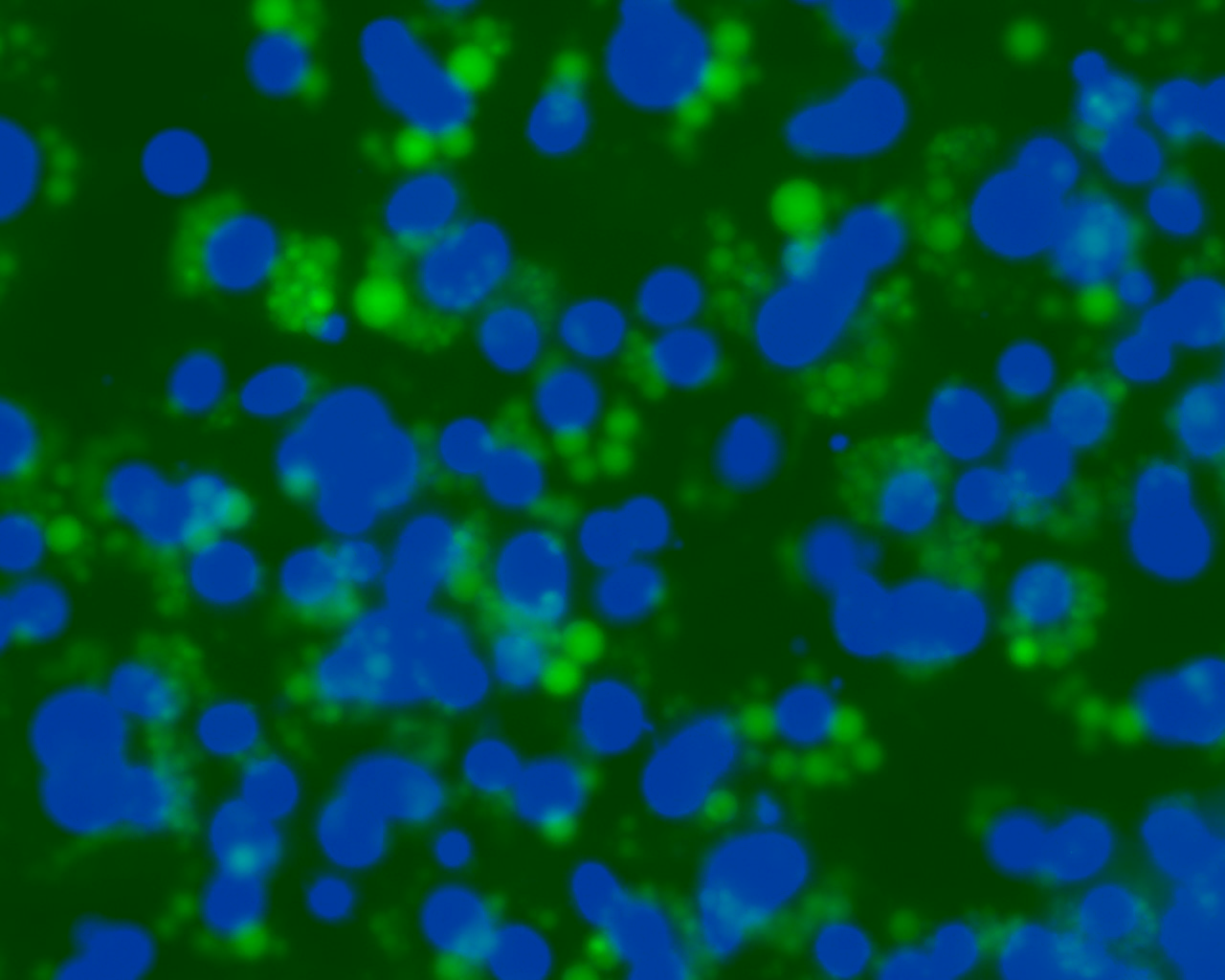

Supplement: Supplementary file 1 [file DataSheet3.ZIP › Original images-3T3-L1+BODIPY493:503/DZF-H-Merge.tif]

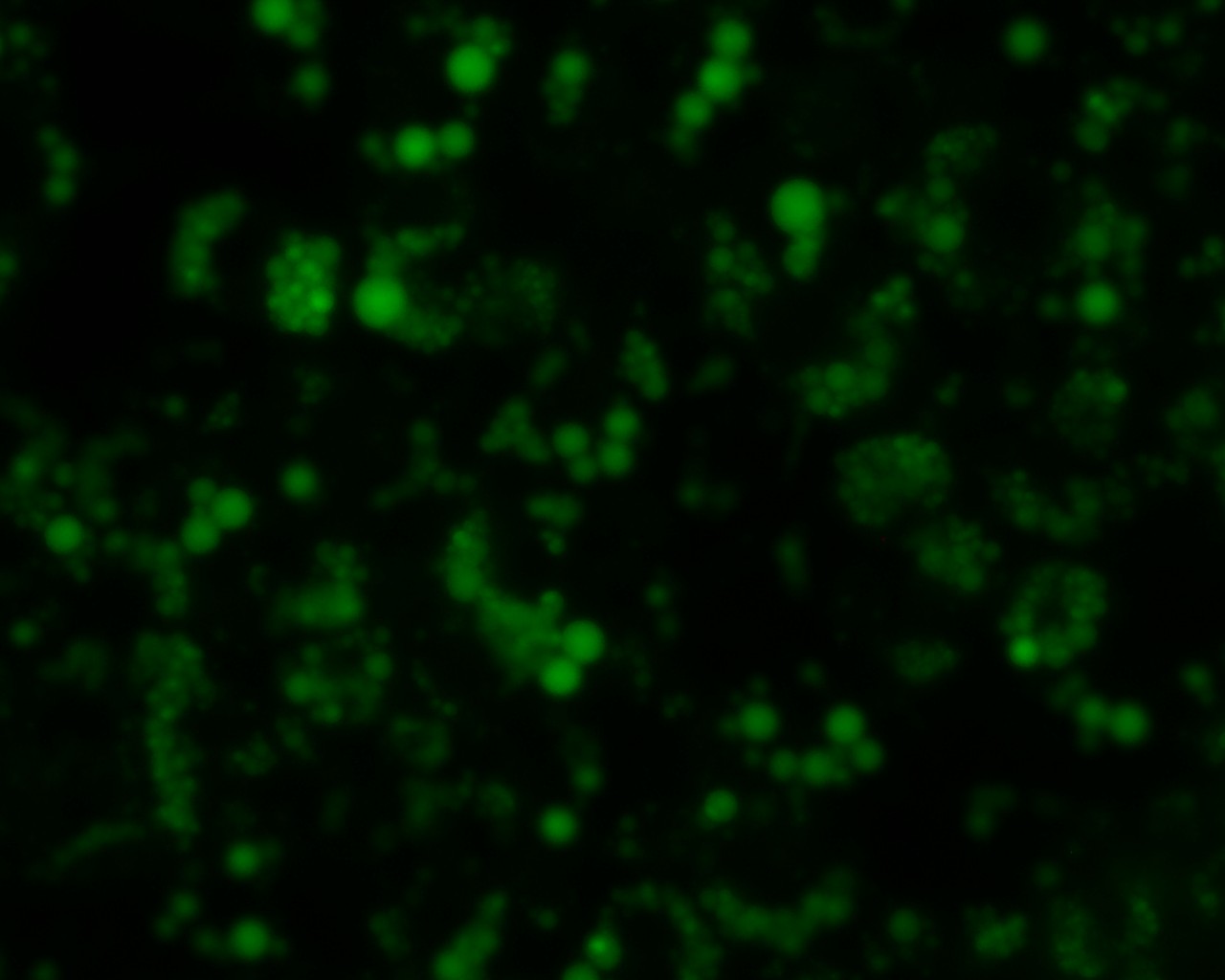

Supplement: Supplementary file 1 [file DataSheet3.ZIP › Original images-3T3-L1+BODIPY493:503/DZF-H-BODIPY.jpg]

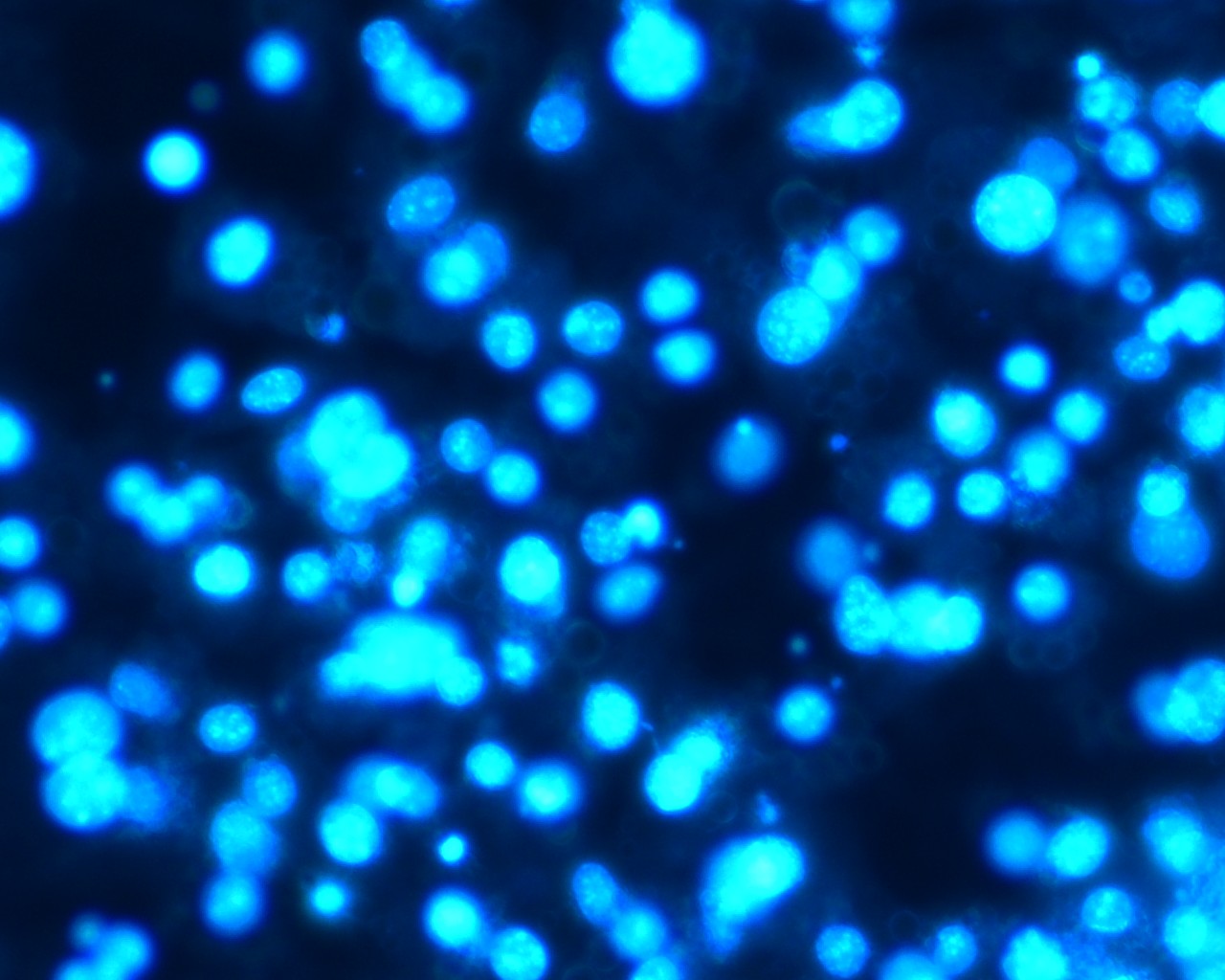

Supplement: Supplementary file 1 [file DataSheet3.ZIP › Original images-3T3-L1+BODIPY493:503/DZF-H-DAPI.jpg]

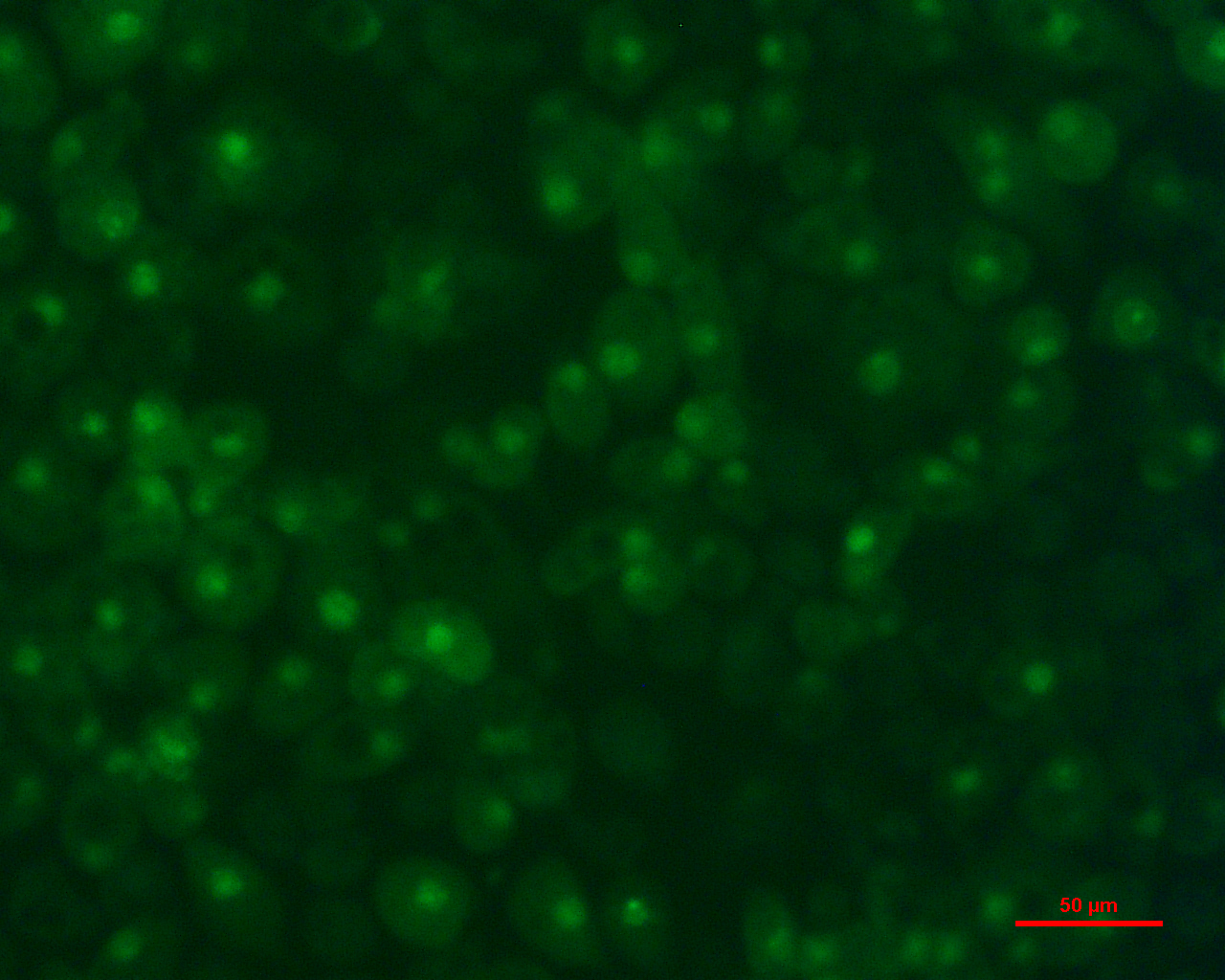

Supplement: Supplementary file 2 [file DataSheet4.ZIP › Original images-3T3-L1+mito-tracker Green/DZF-H-Dark.jpg]

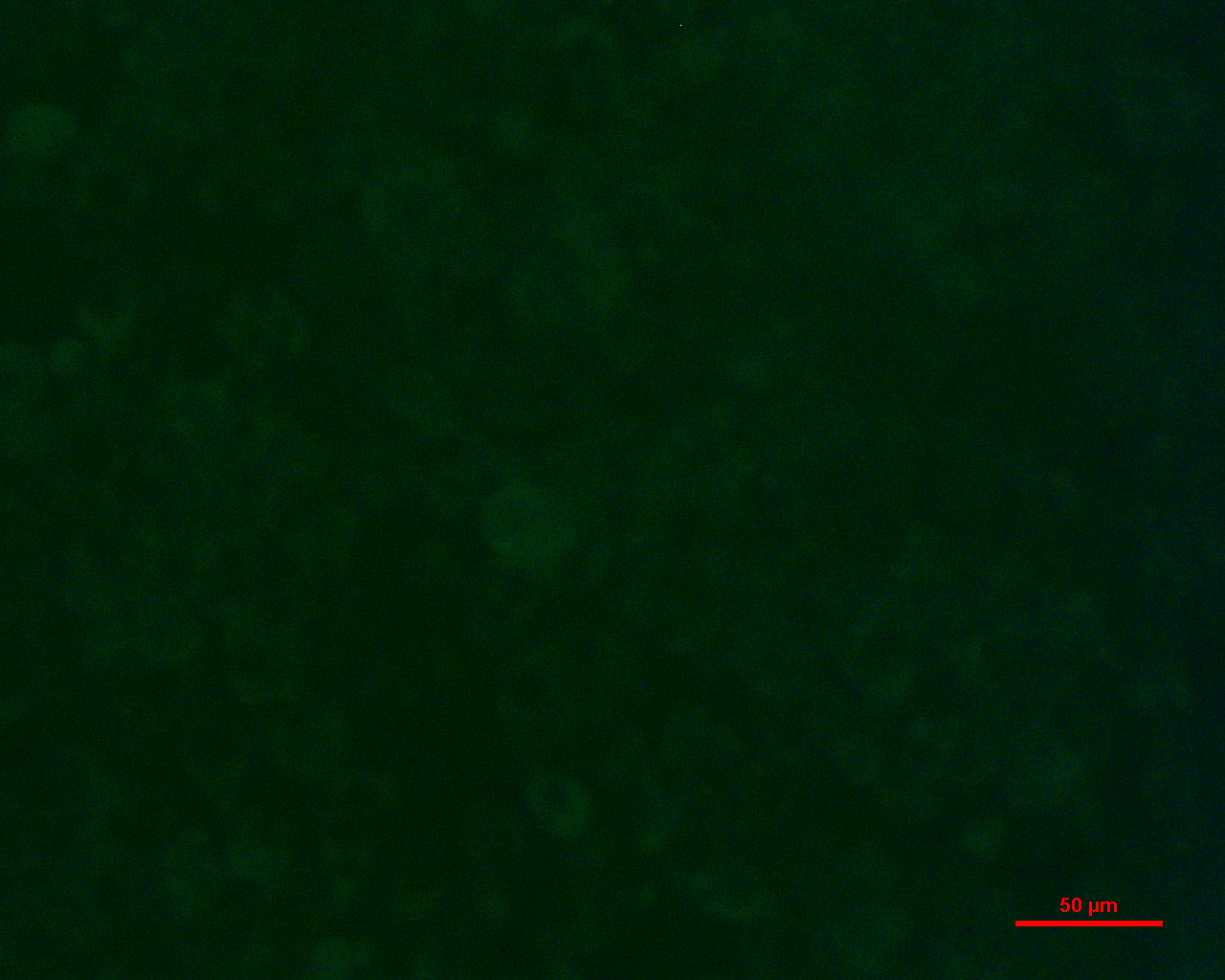

Supplement: Supplementary file 2 [file DataSheet4.ZIP › Original images-3T3-L1+mito-tracker Green/Veh-Dark.jpg]

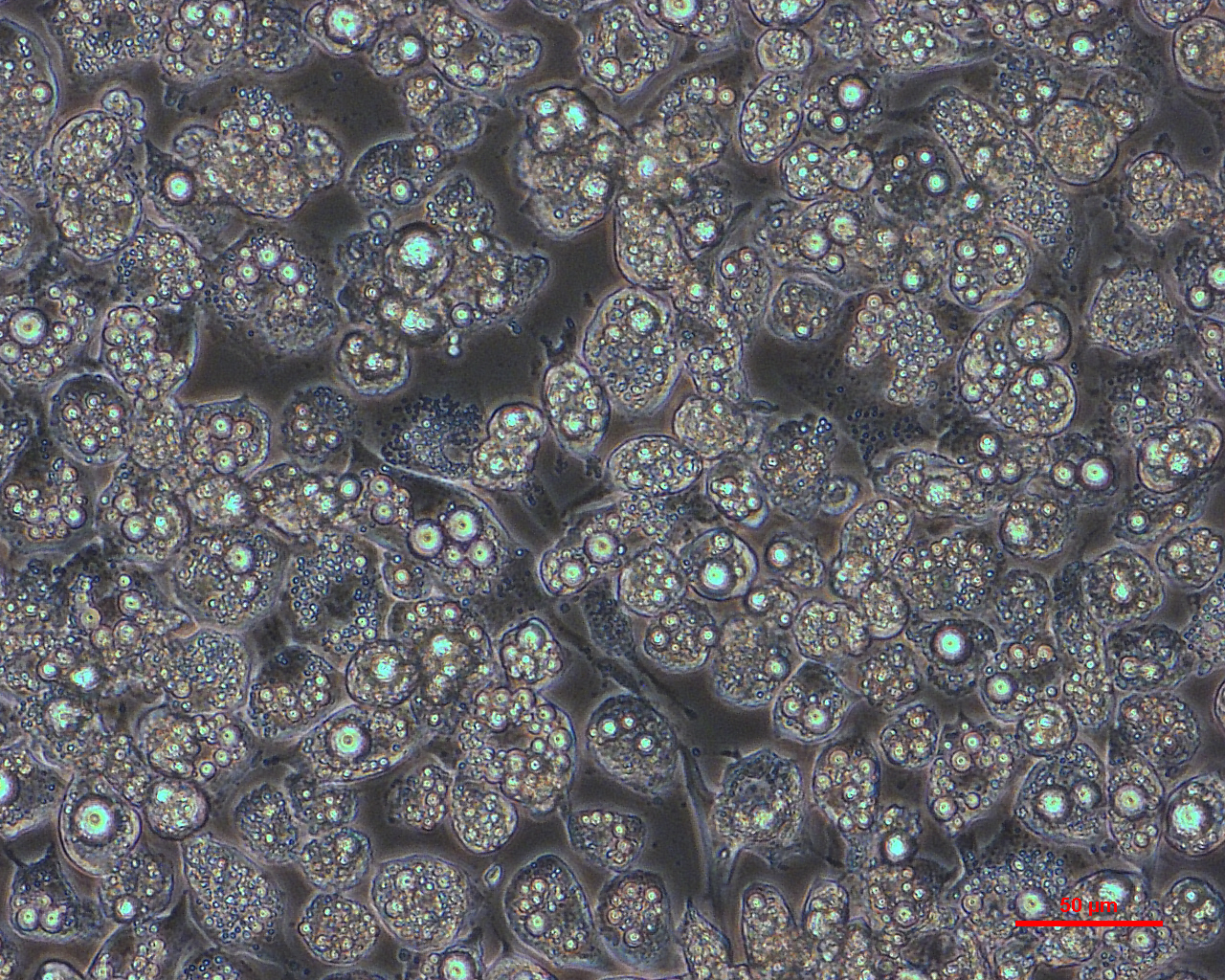

Supplement: Supplementary file 2 [file DataSheet4.ZIP › Original images-3T3-L1+mito-tracker Green/DZF-H-Bright.jpg]

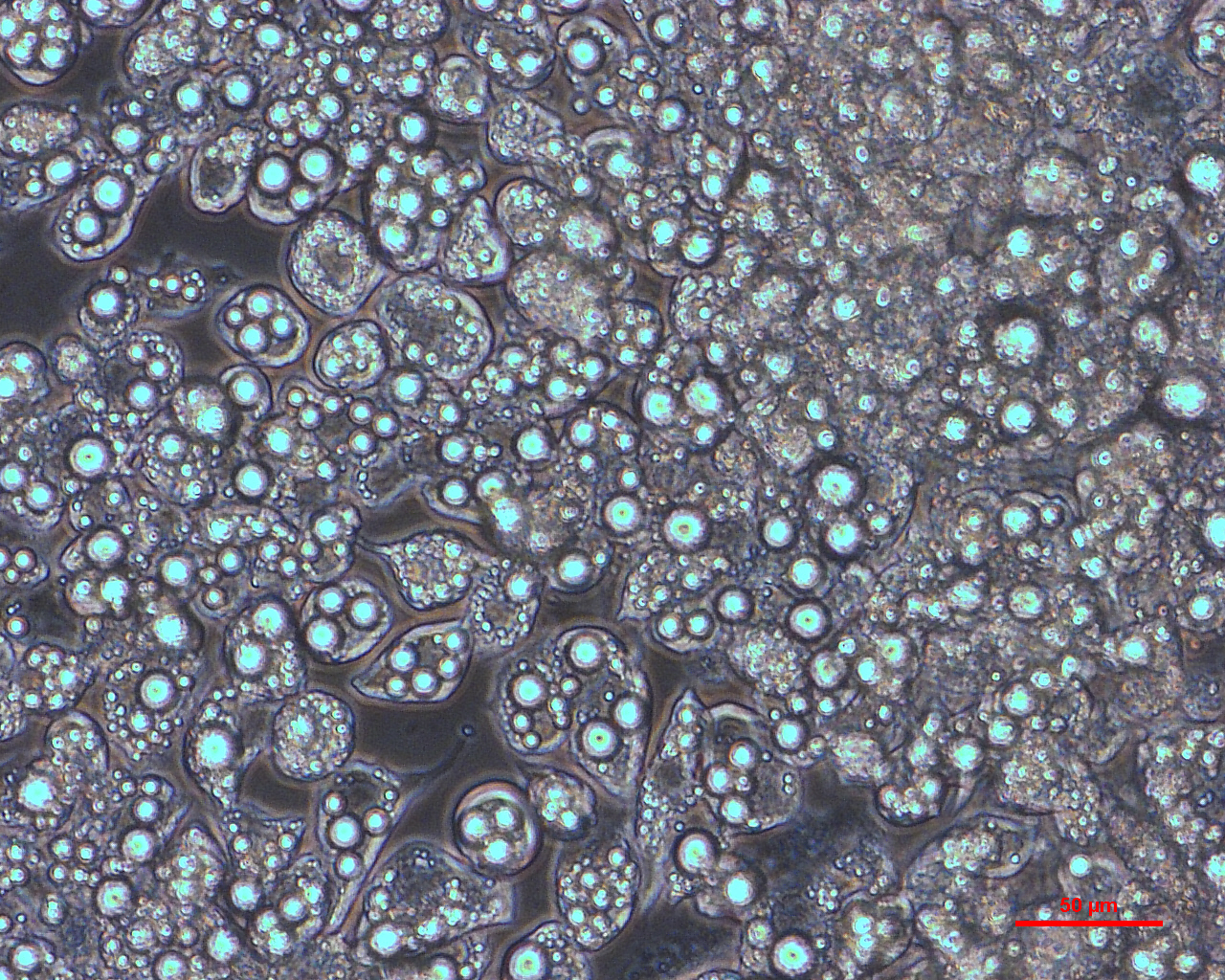

Supplement: Supplementary file 2 [file DataSheet4.ZIP › Original images-3T3-L1+mito-tracker Green/Veh-Bright.jpg]

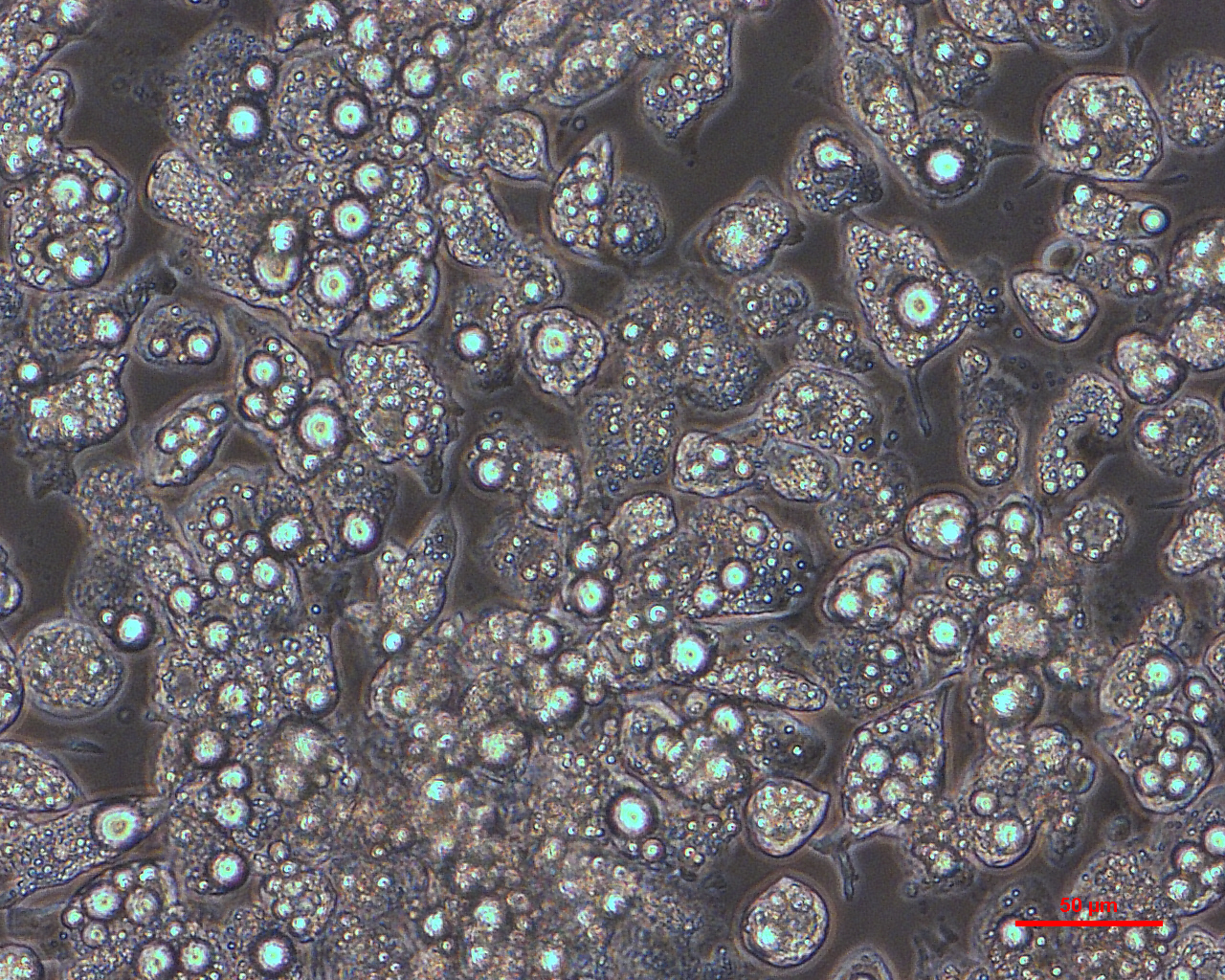

Supplement: Supplementary file 2 [file DataSheet4.ZIP › Original images-3T3-L1+mito-tracker Green/DZF-L-Bright.jpg]

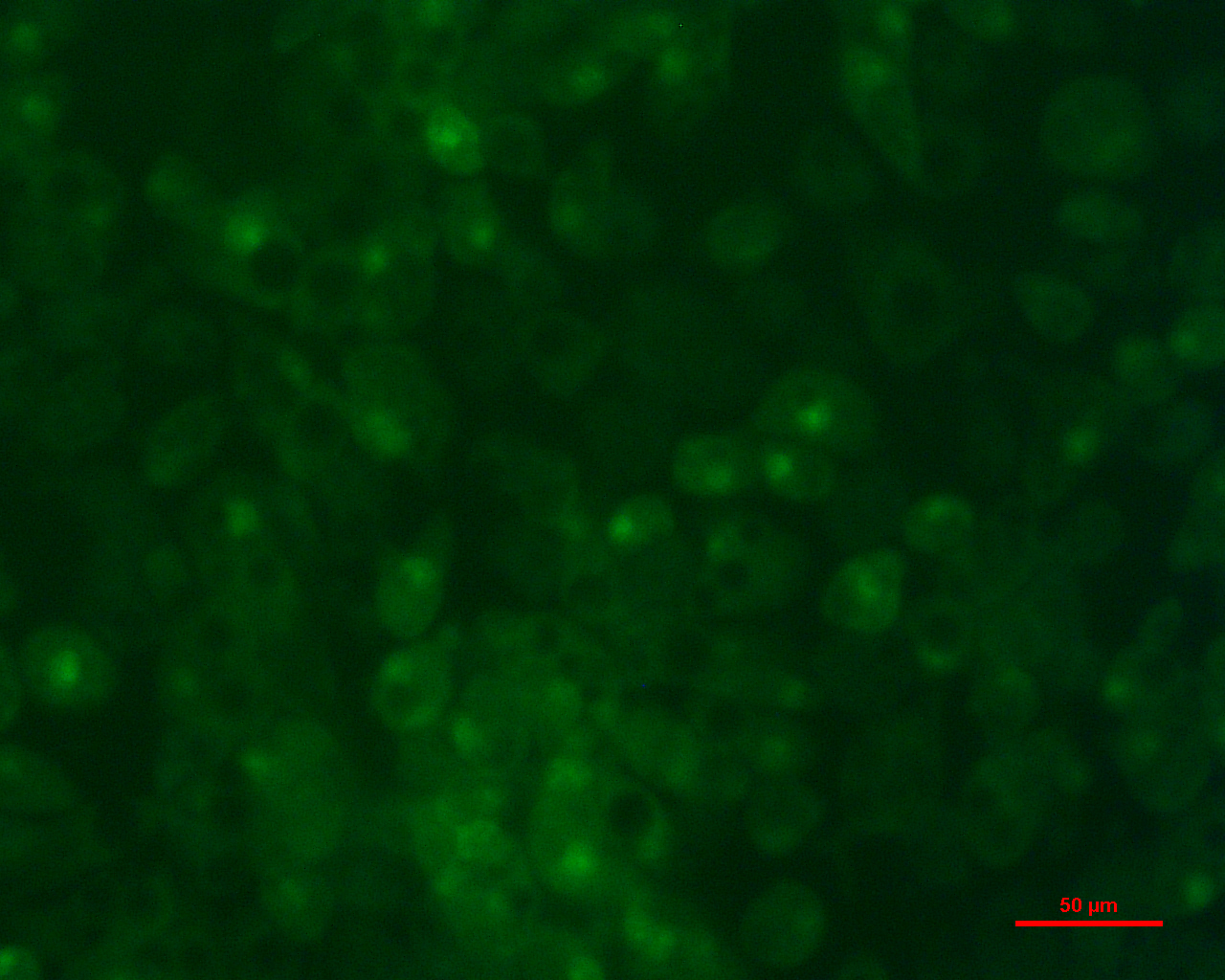

Supplement: Supplementary file 2 [file DataSheet4.ZIP › Original images-3T3-L1+mito-tracker Green/DZF-L-Dark.jpg]

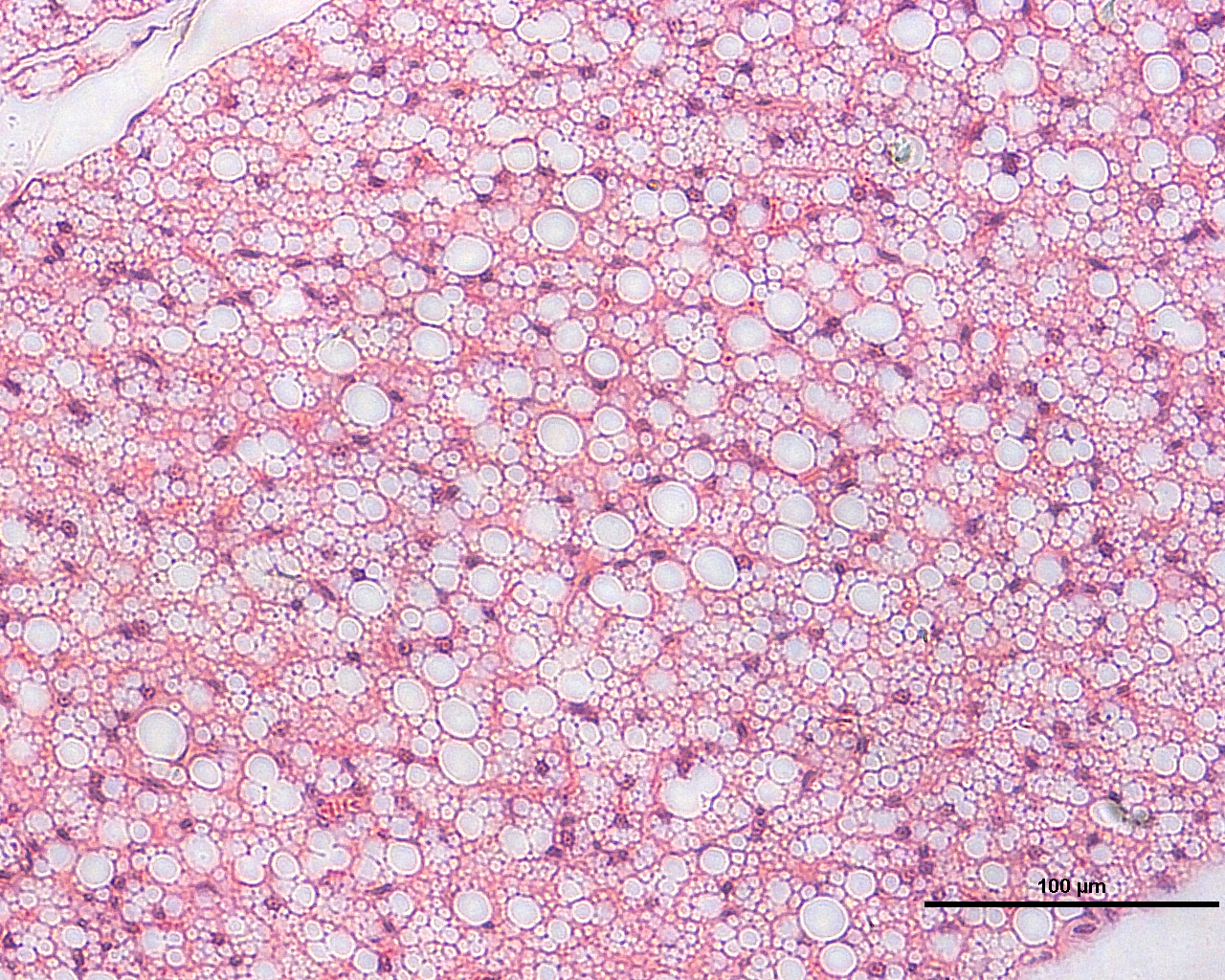

Supplement: Supplementary file 4 [file DataSheet6.ZIP › Original images-H&E/NCD-BAT.jpg]

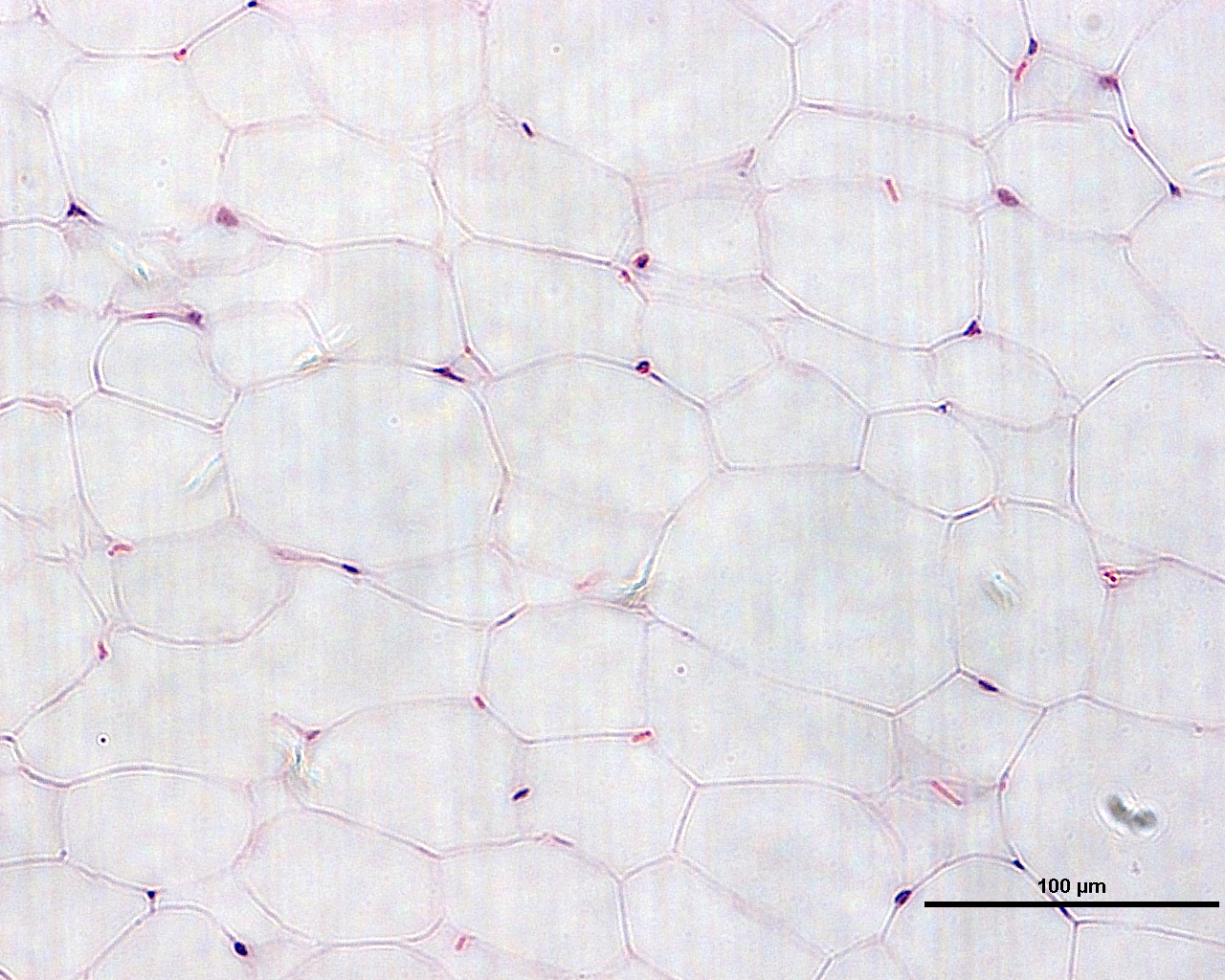

Supplement: Supplementary file 4 [file DataSheet6.ZIP › Original images-H&E/HFD+Veh-iWAT.jpg]

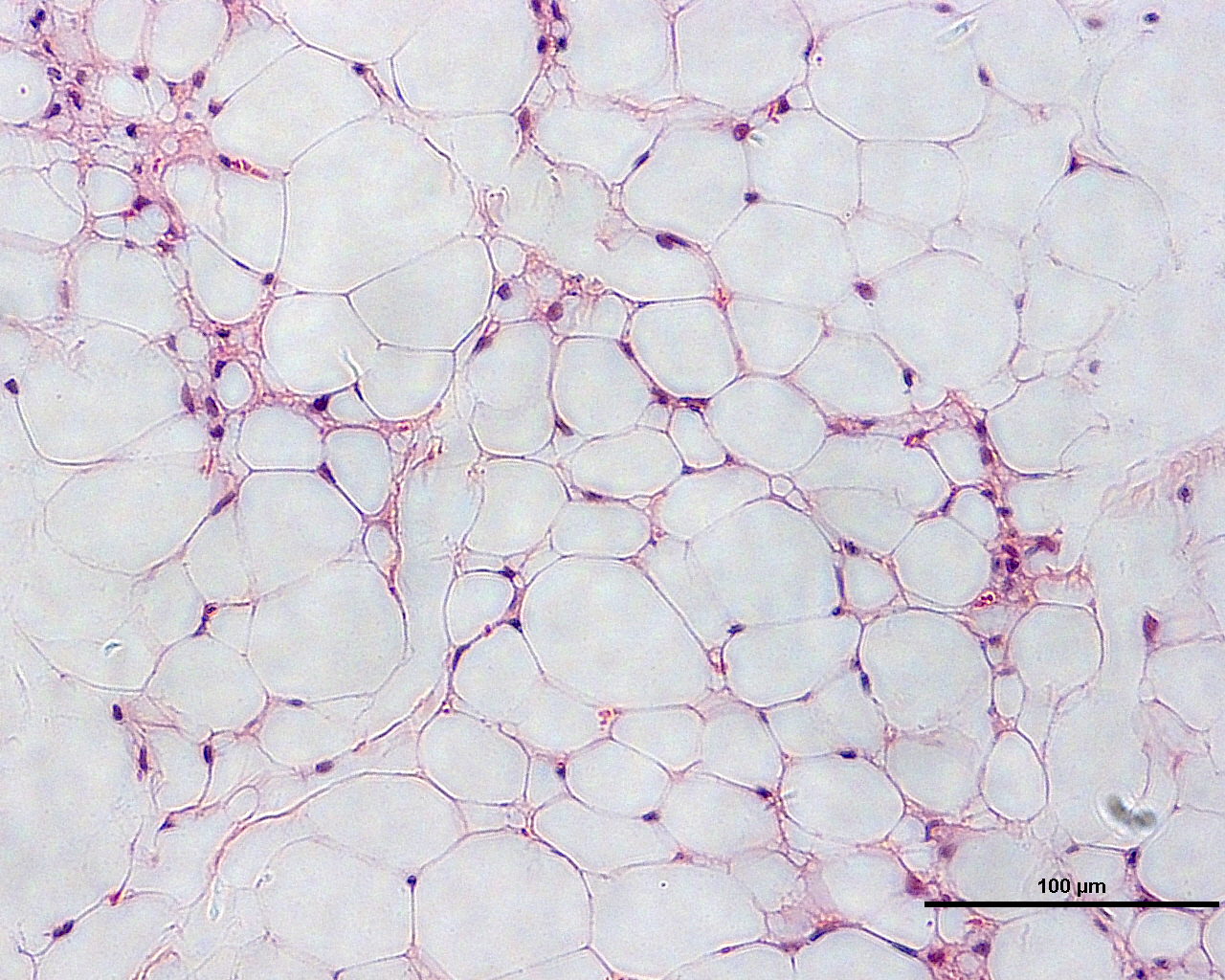

Supplement: Supplementary file 4 [file DataSheet6.ZIP › Original images-H&E/NCD-iWAT.jpg]

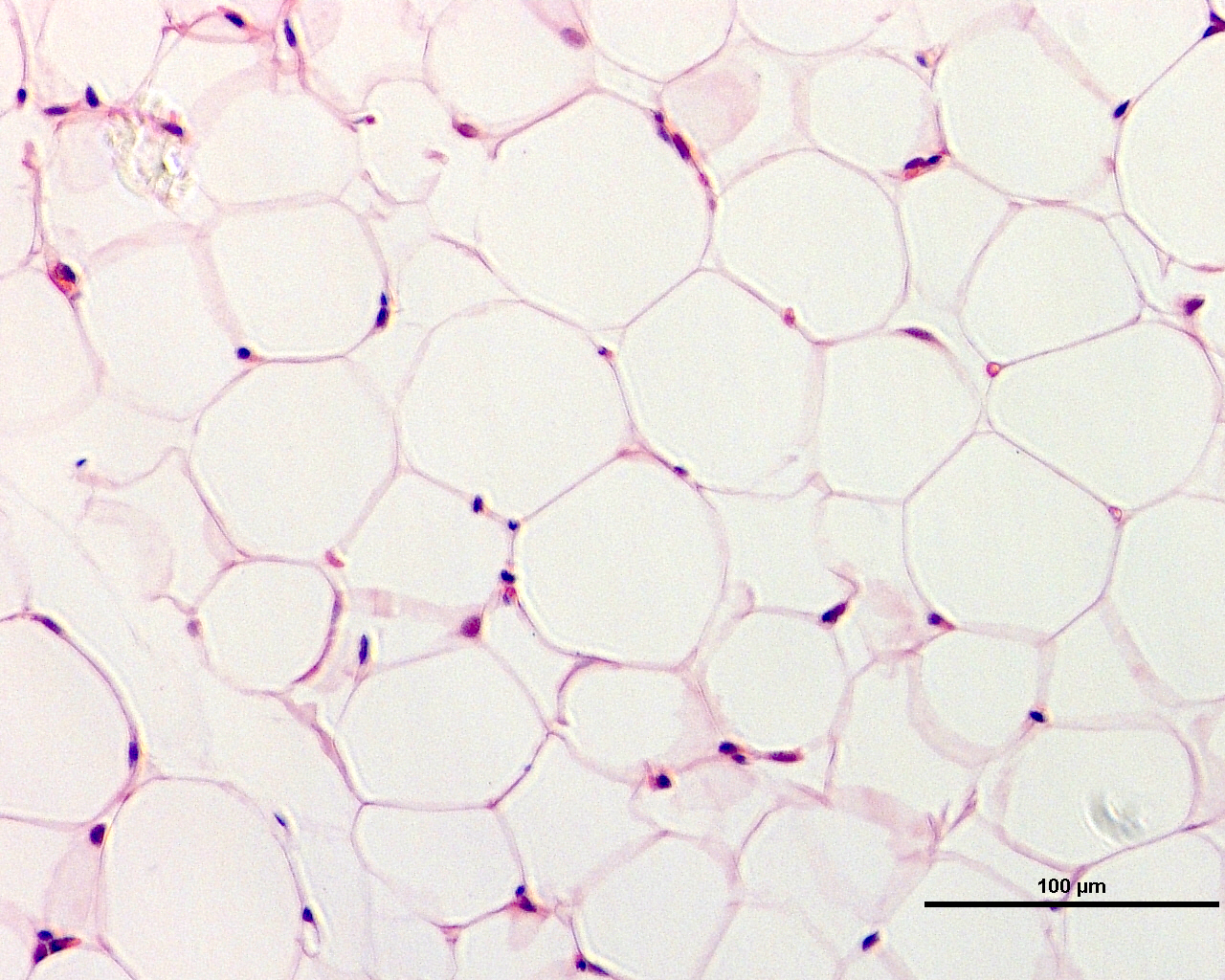

Supplement: Supplementary file 4 [file DataSheet6.ZIP › Original images-H&E/NCD-eWAT.jpg]

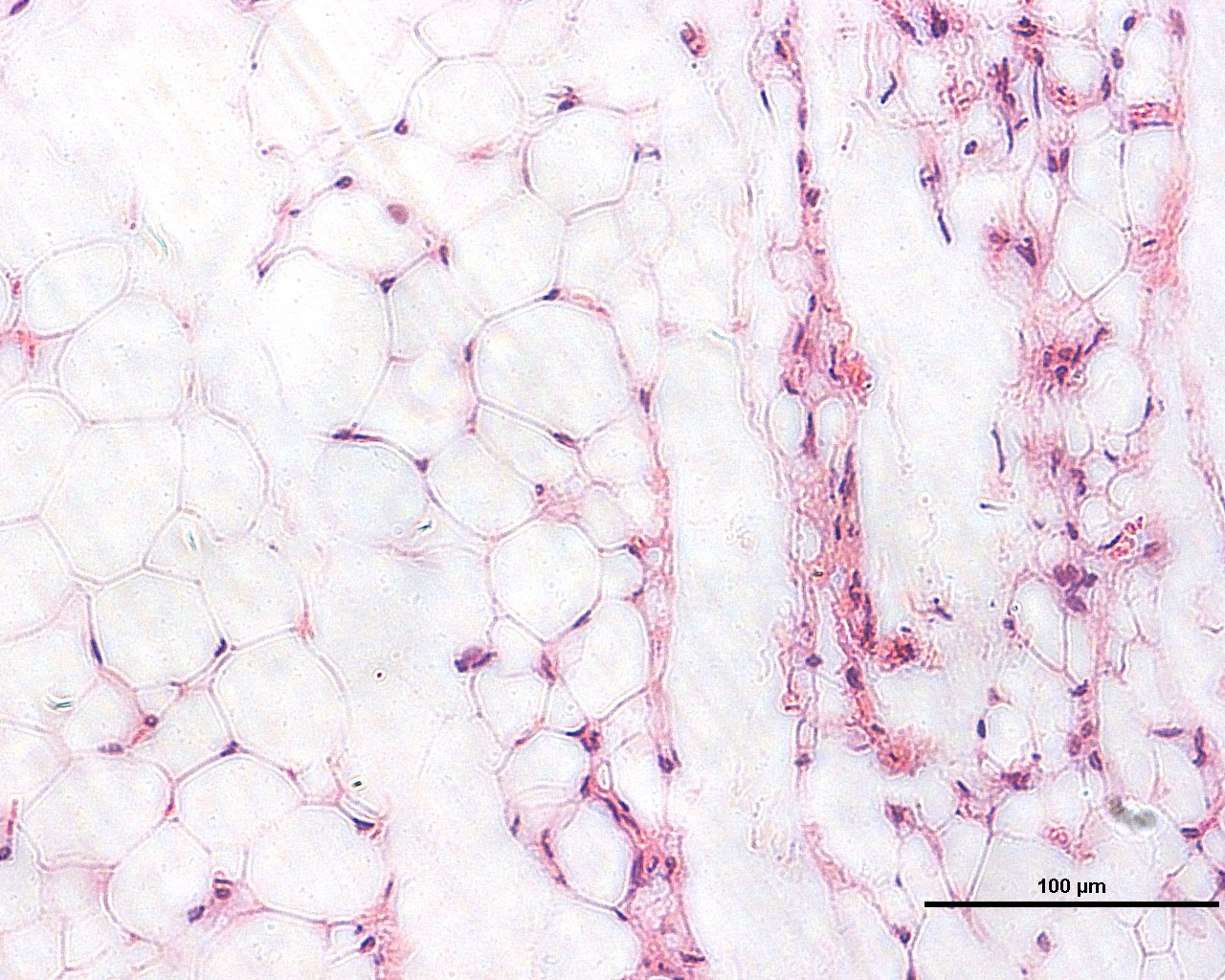

Supplement: Supplementary file 4 [file DataSheet6.ZIP › Original images-H&E/HFD+DZF-H-iWAT.jpg]

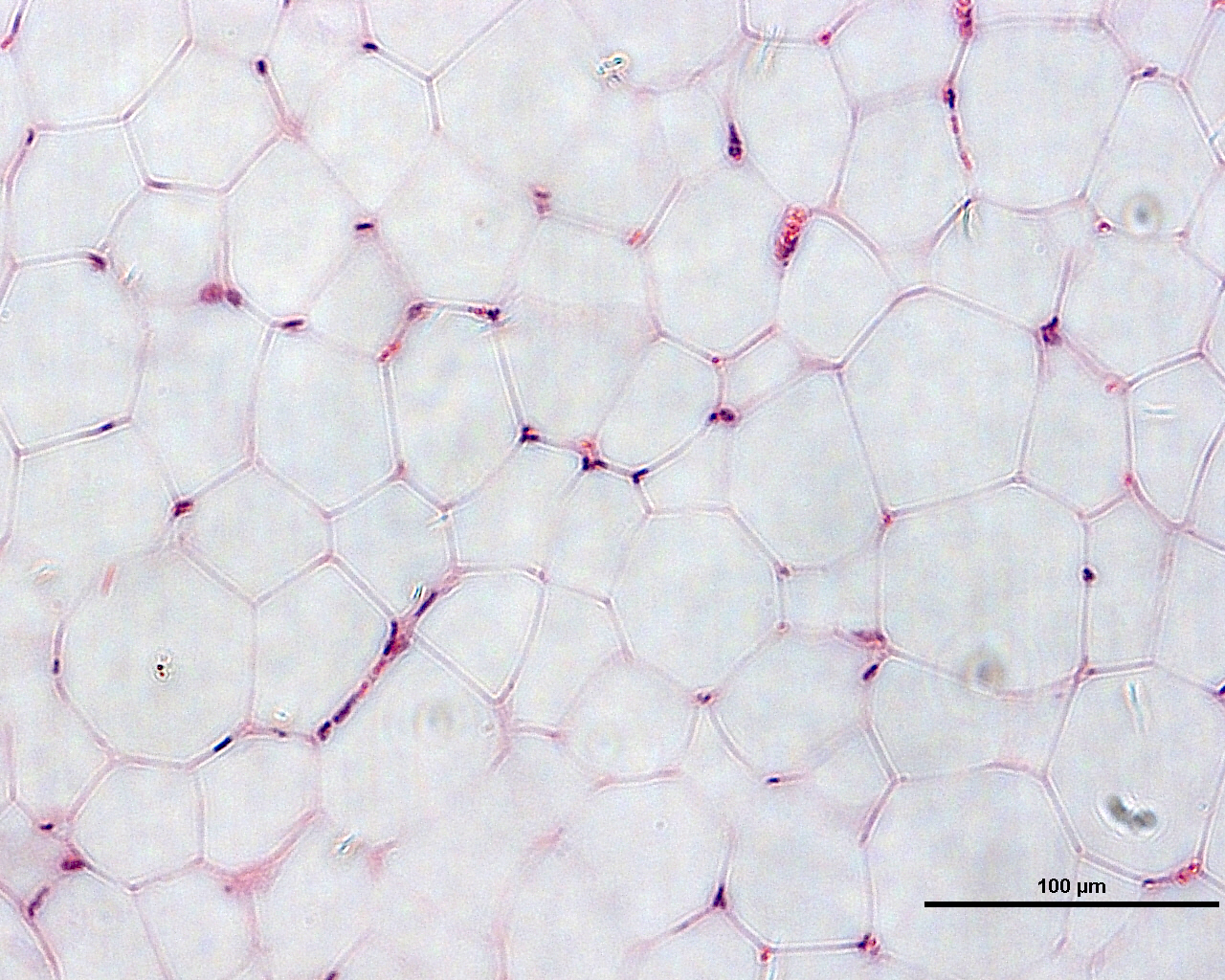

Supplement: Supplementary file 4 [file DataSheet6.ZIP › Original images-H&E/NCD-pWAT.jpg]

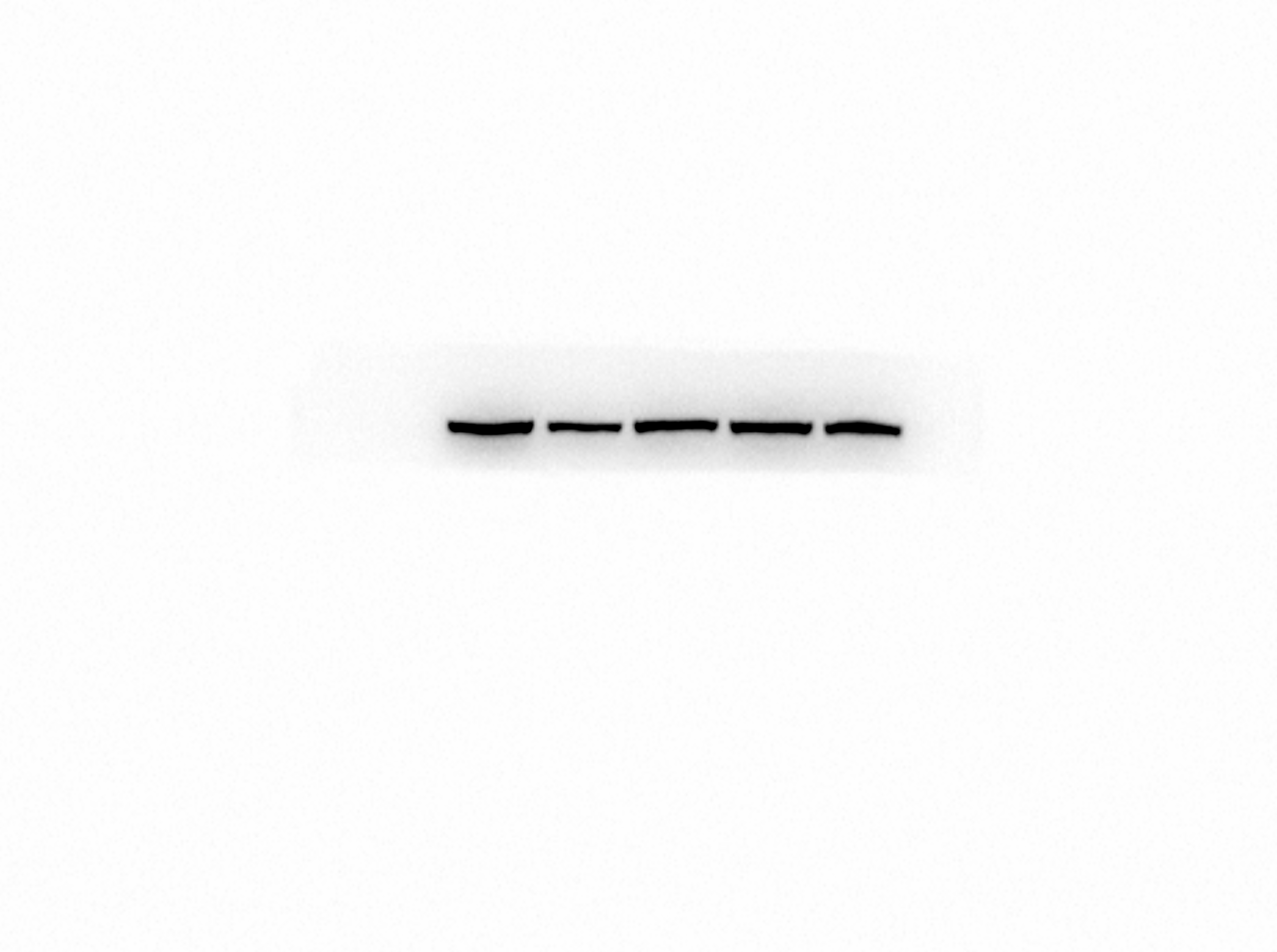

Supplement: Supplementary file 5 [file DataSheet2.ZIP › Original images--WB /H89-B-actin.tif]

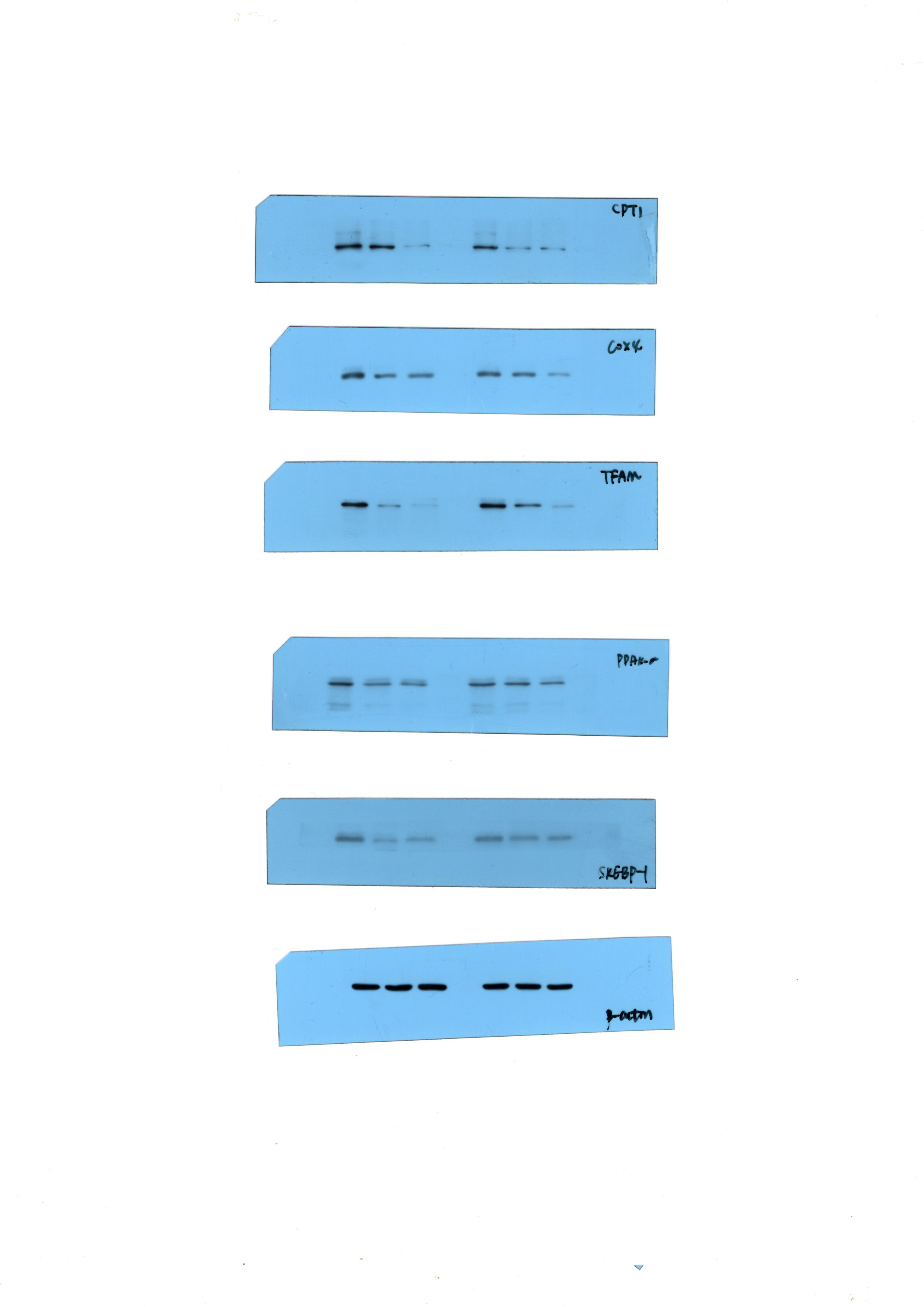

Supplement: Supplementary file 5 [file DataSheet2.ZIP › Original images--WB /SREBP1-blue.jpg]

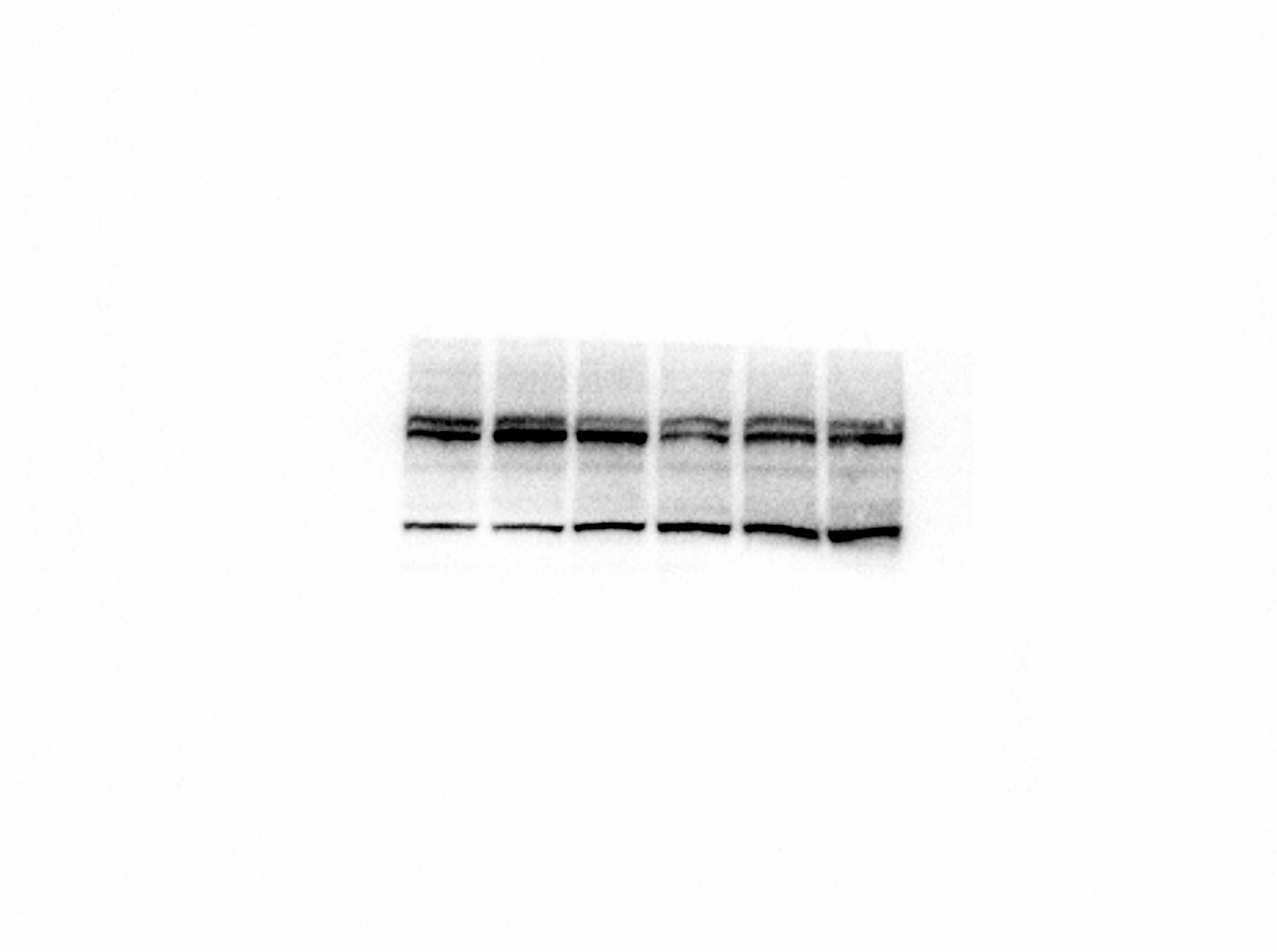

Supplement: Supplementary file 5 [file DataSheet2.ZIP › Original images--WB /PGC1a.tif]

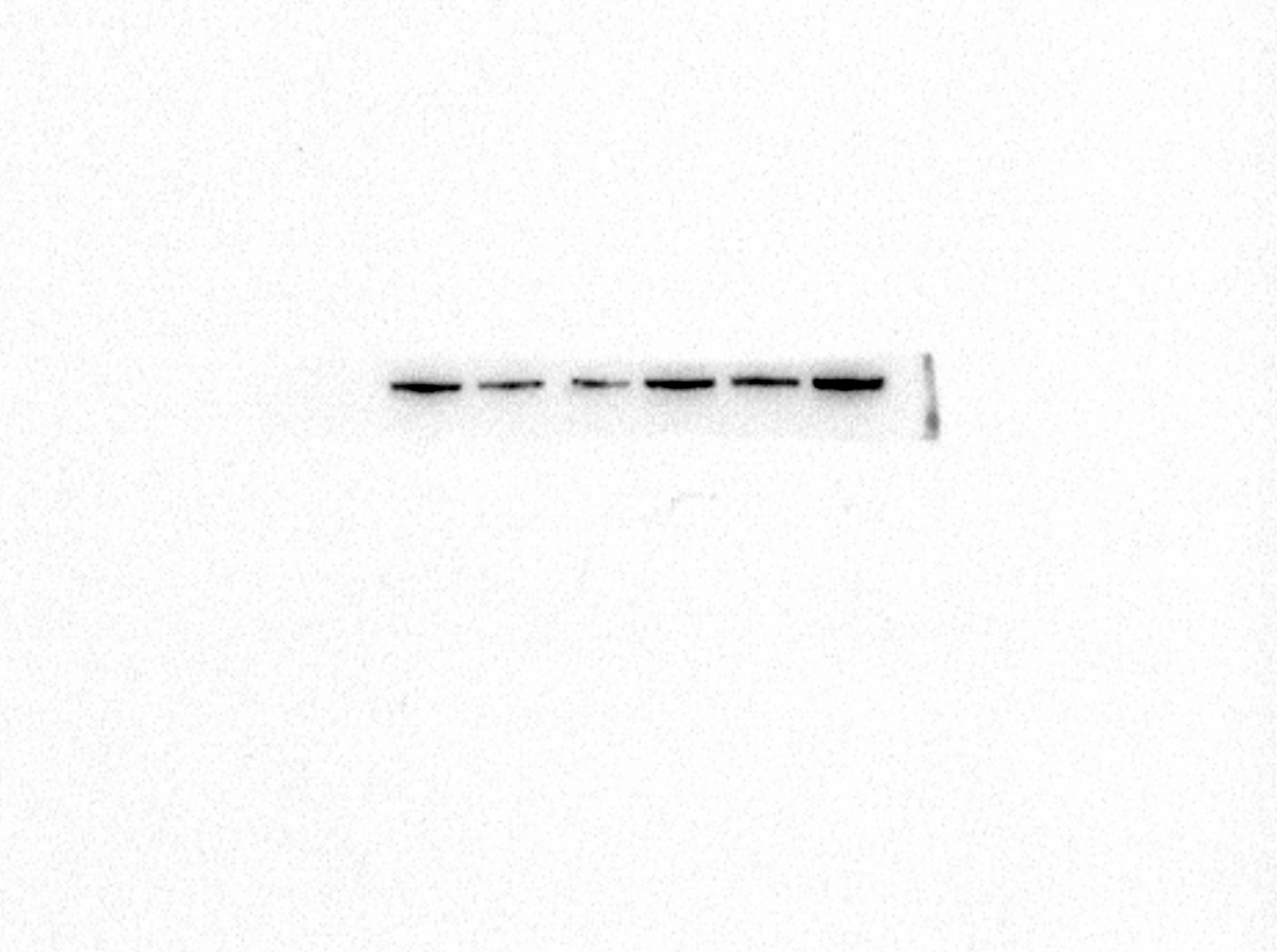

Supplement: Supplementary file 5 [file DataSheet2.ZIP › Original images--WB /CREB.tif]

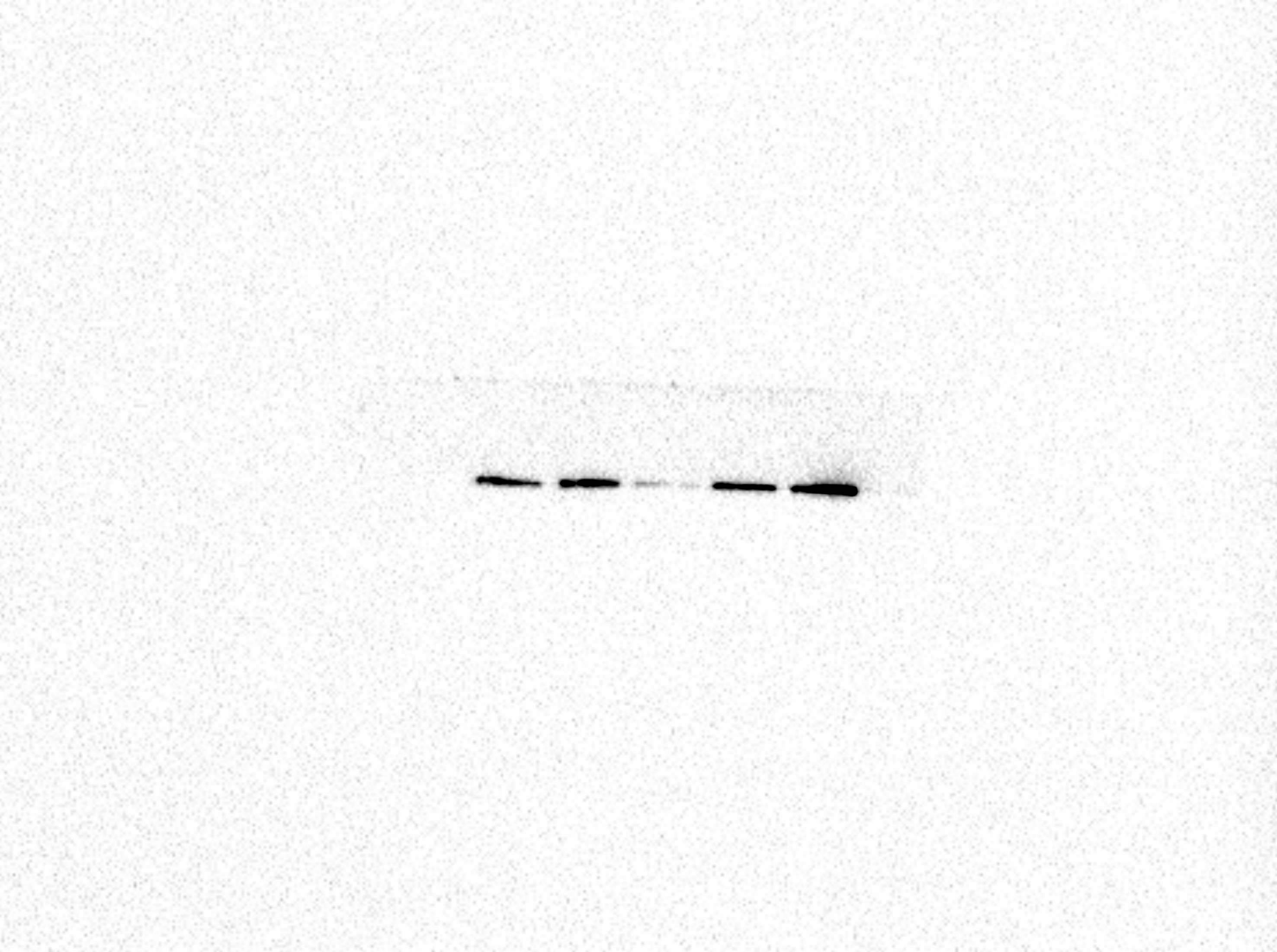

Supplement: Supplementary file 5 [file DataSheet2.ZIP › Original images--WB /H89-P-creab.tif]

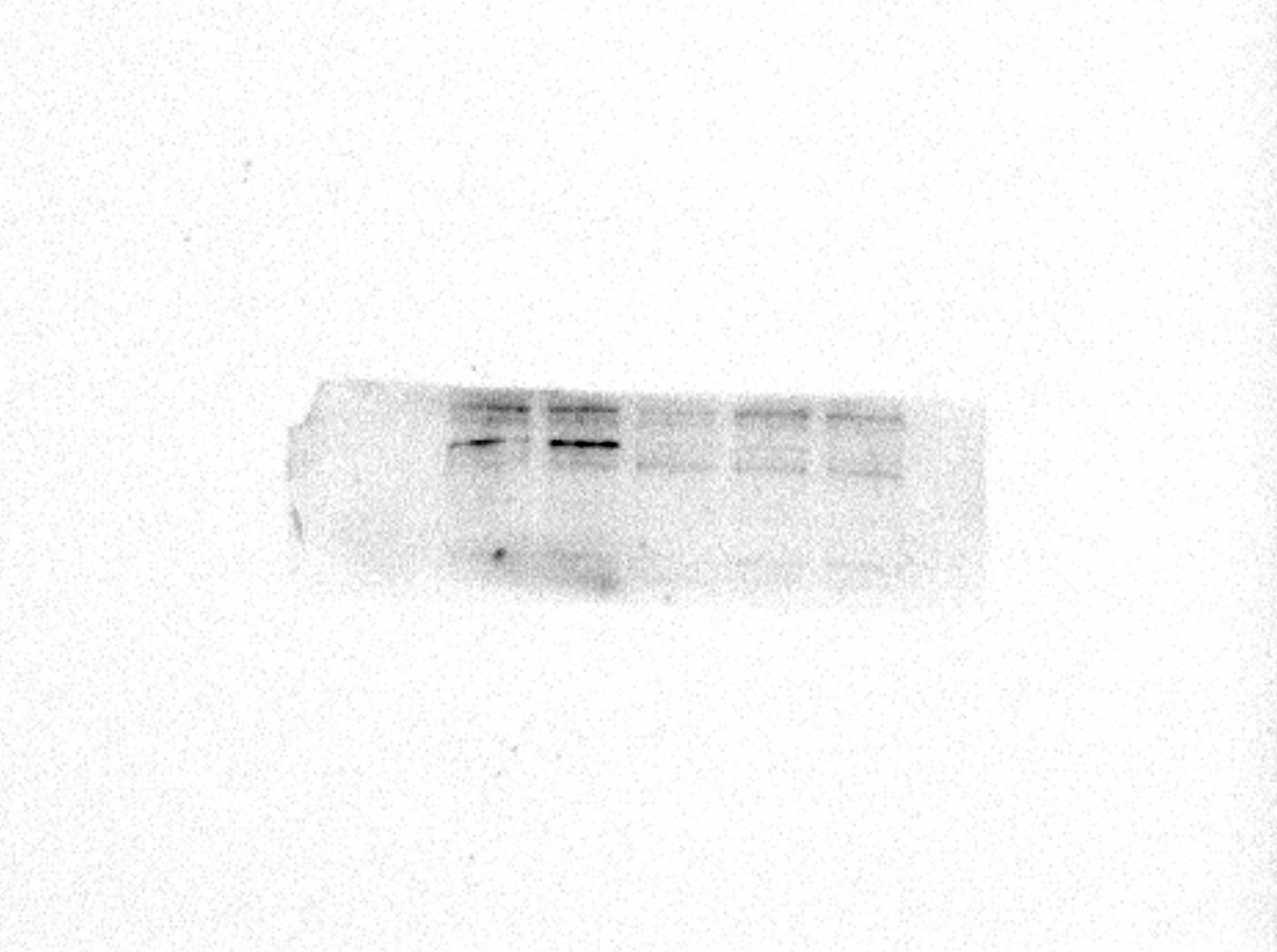

Supplement: Supplementary file 5 [file DataSheet2.ZIP › Original images--WB /H89-UCP1.tif]

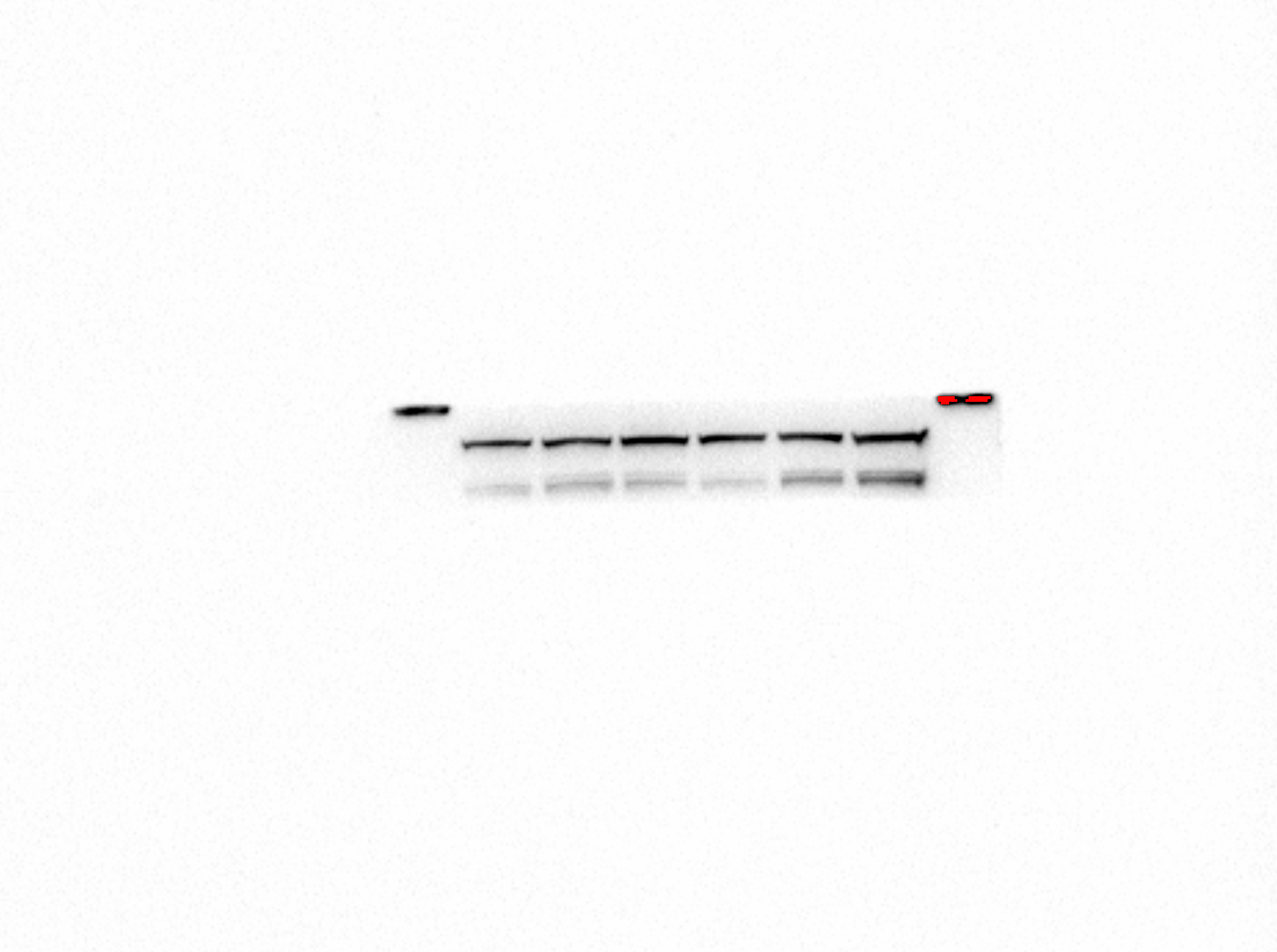

Supplement: Supplementary file 5 [file DataSheet2.ZIP › Original images--WB /PKA.tif]

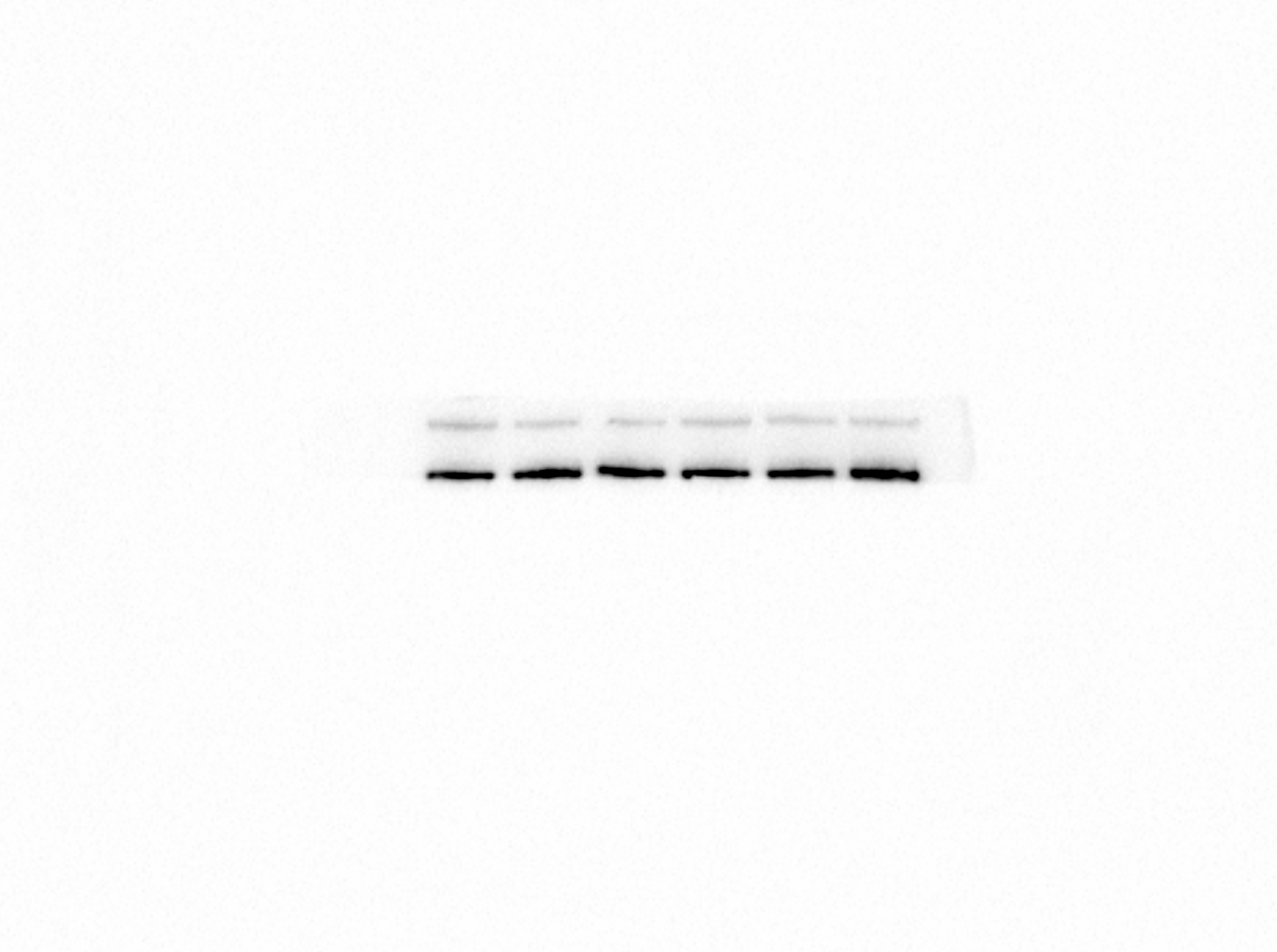

Supplement: Supplementary file 5 [file DataSheet2.ZIP › Original images--WB /pCREB.tif]

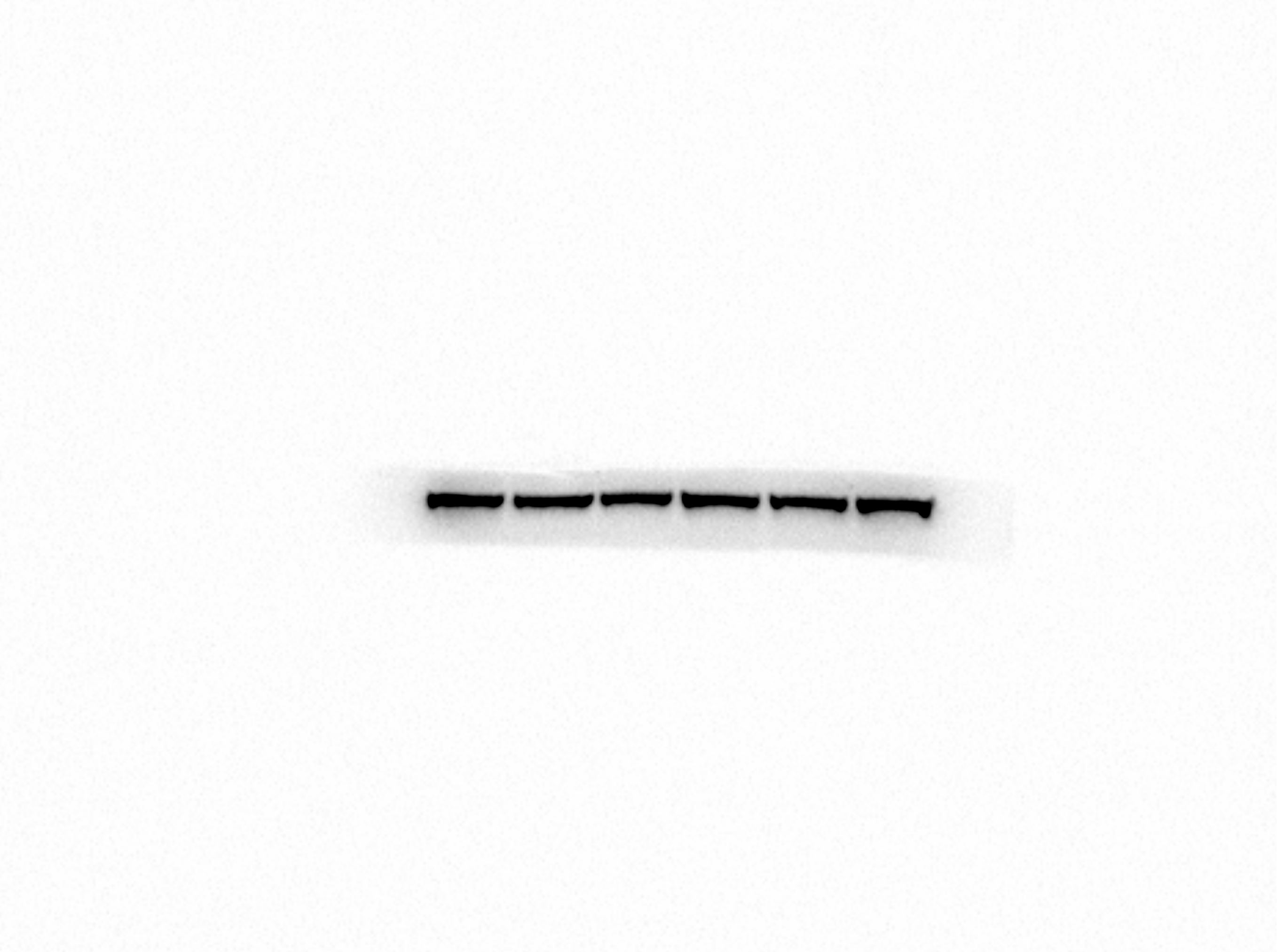

Supplement: Supplementary file 5 [file DataSheet2.ZIP › Original images--WB /3 B-actin.tif]

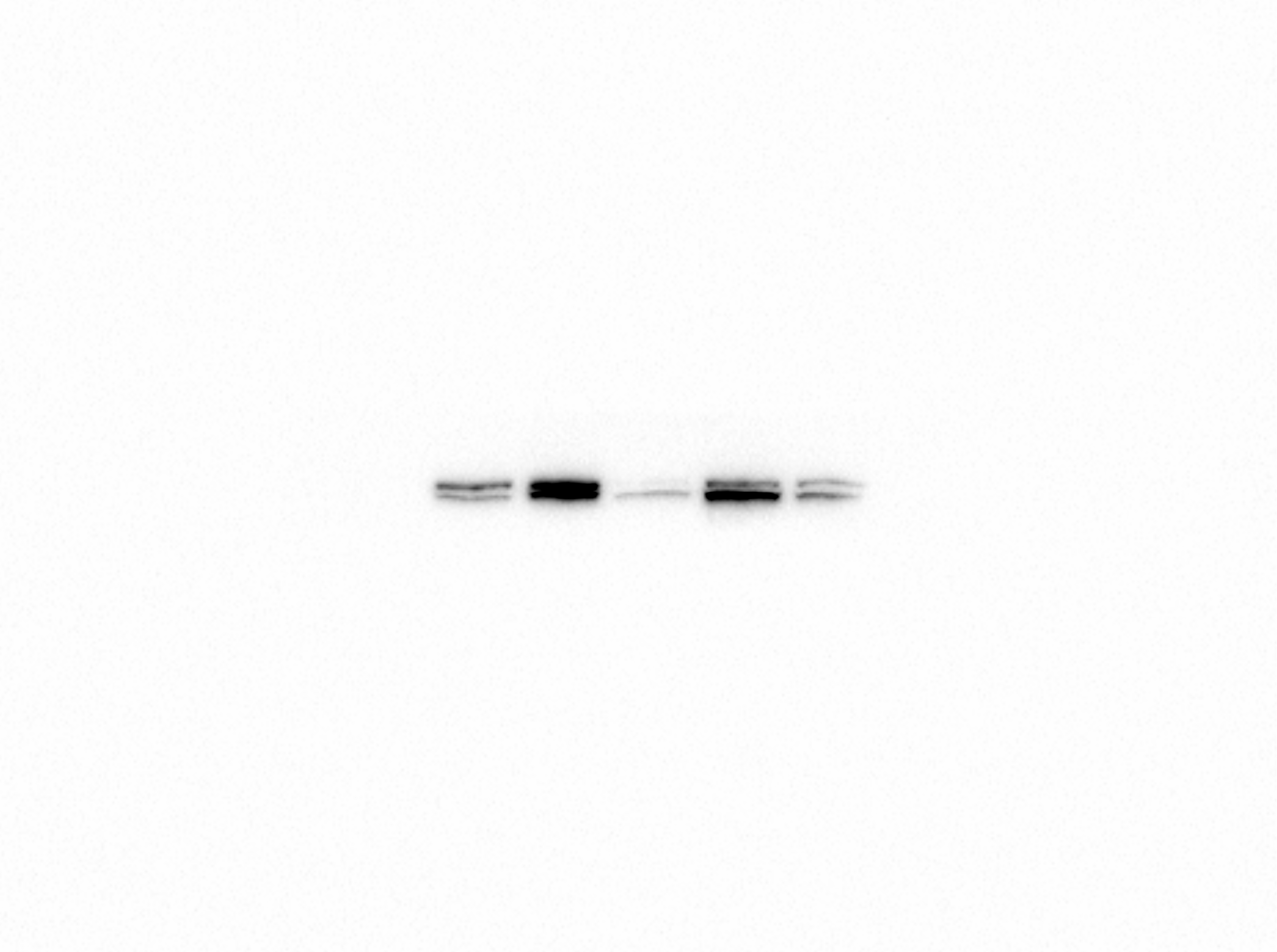

Supplement: Supplementary file 5 [file DataSheet2.ZIP › Original images--WB /H89-CREB-1.tif]

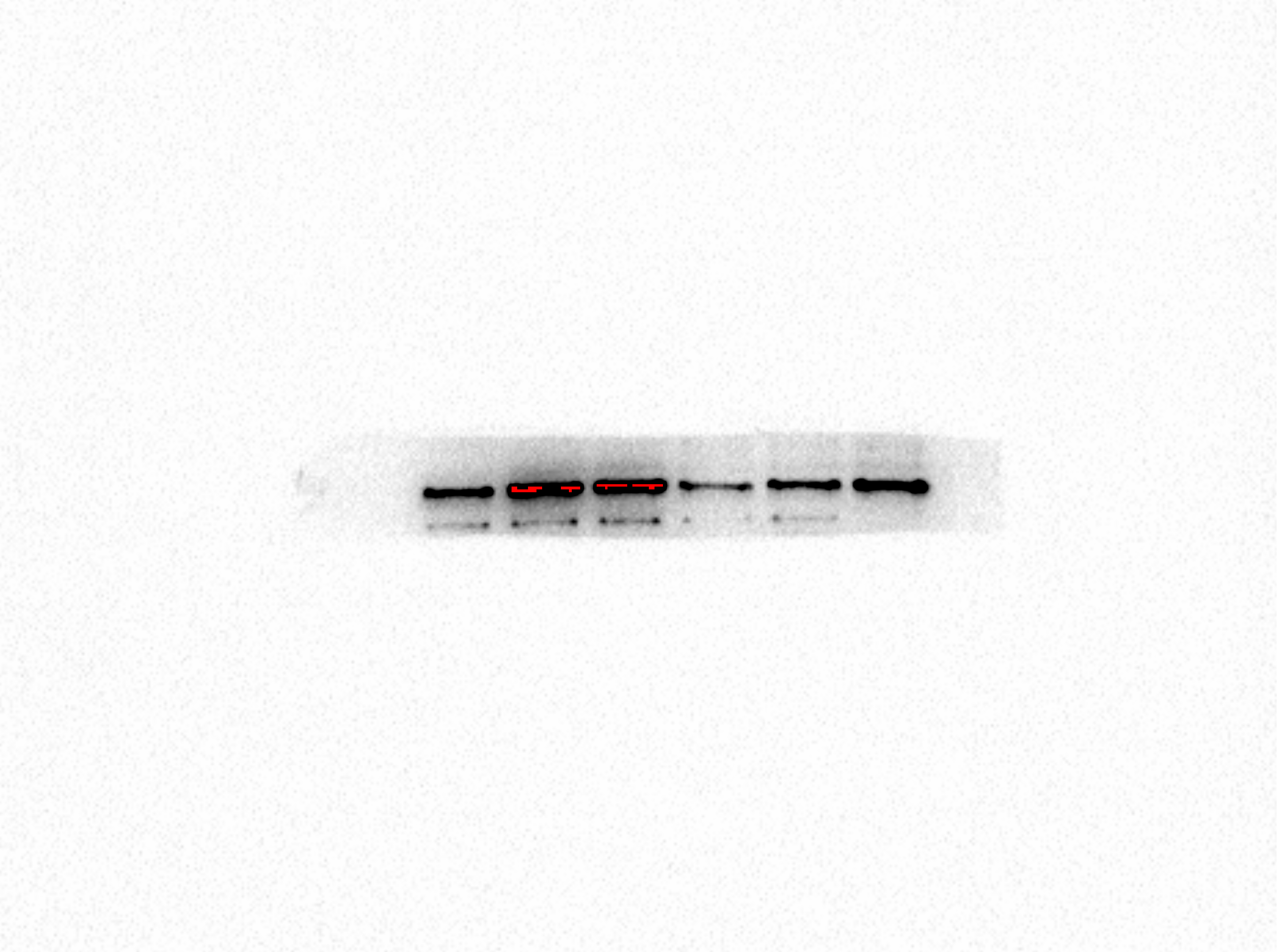

Supplement: Supplementary file 5 [file DataSheet2.ZIP › Original images--WB /UCP1.tif]

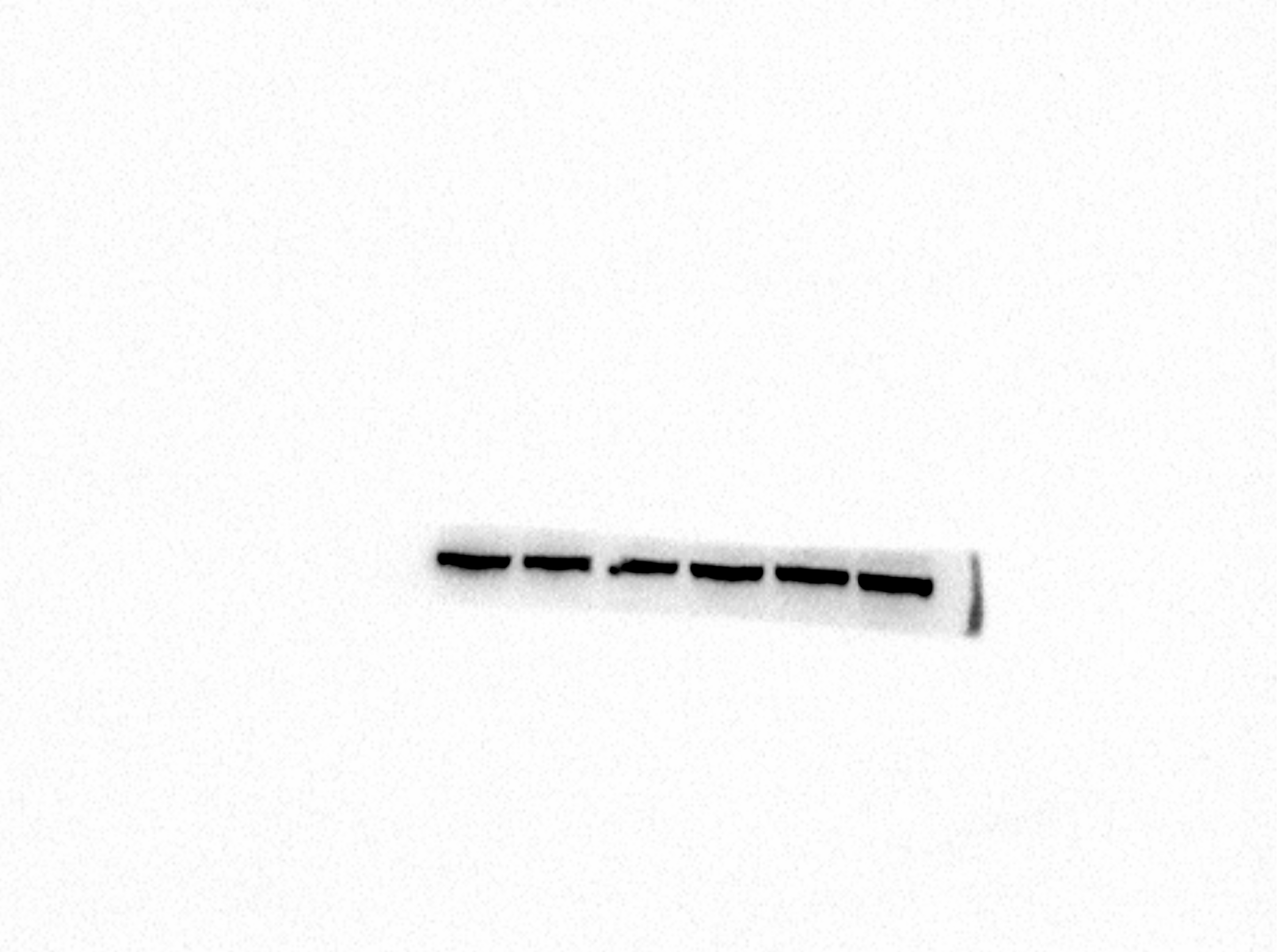

Supplement: Supplementary file 5 [file DataSheet2.ZIP › Original images--WB /1 B-actin.tif]

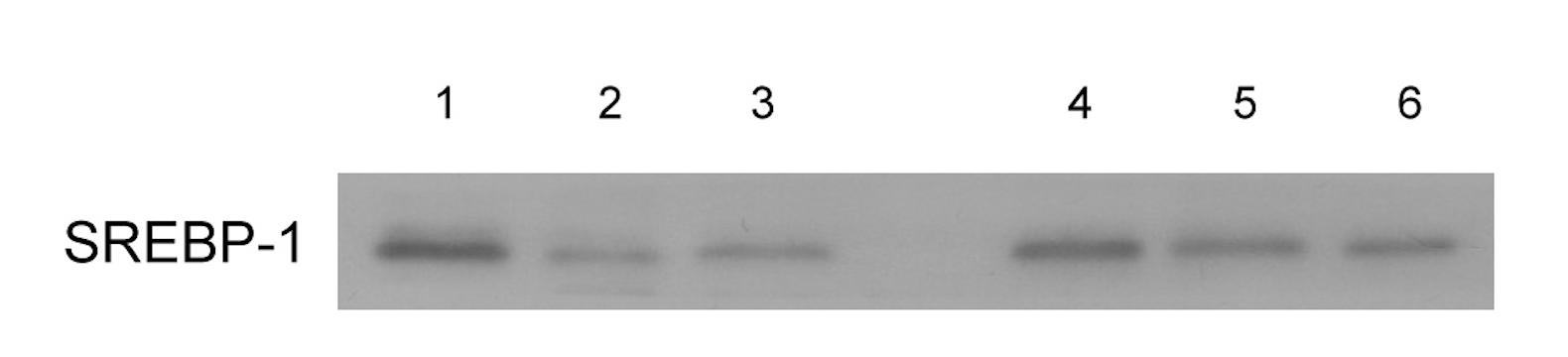

Supplement: Supplementary file 5 [file DataSheet2.ZIP › Original images--WB /SREBP-1-WB.tiff]

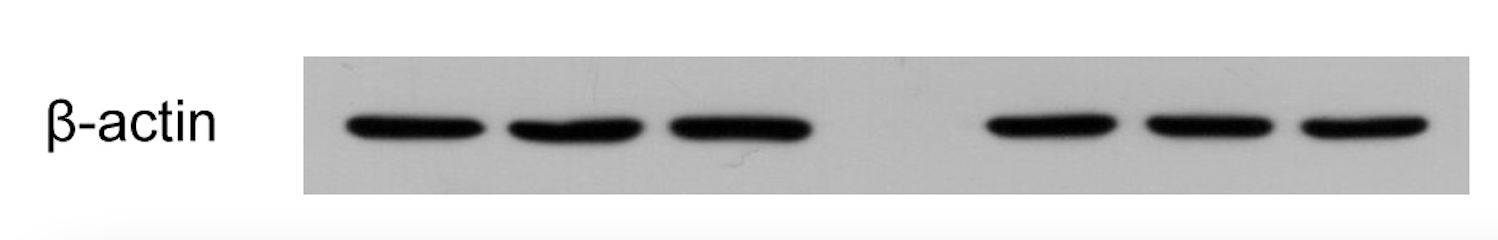

Supplement: Supplementary file 5 [file DataSheet2.ZIP › Original images--WB /b-actin(srebp-1).png]

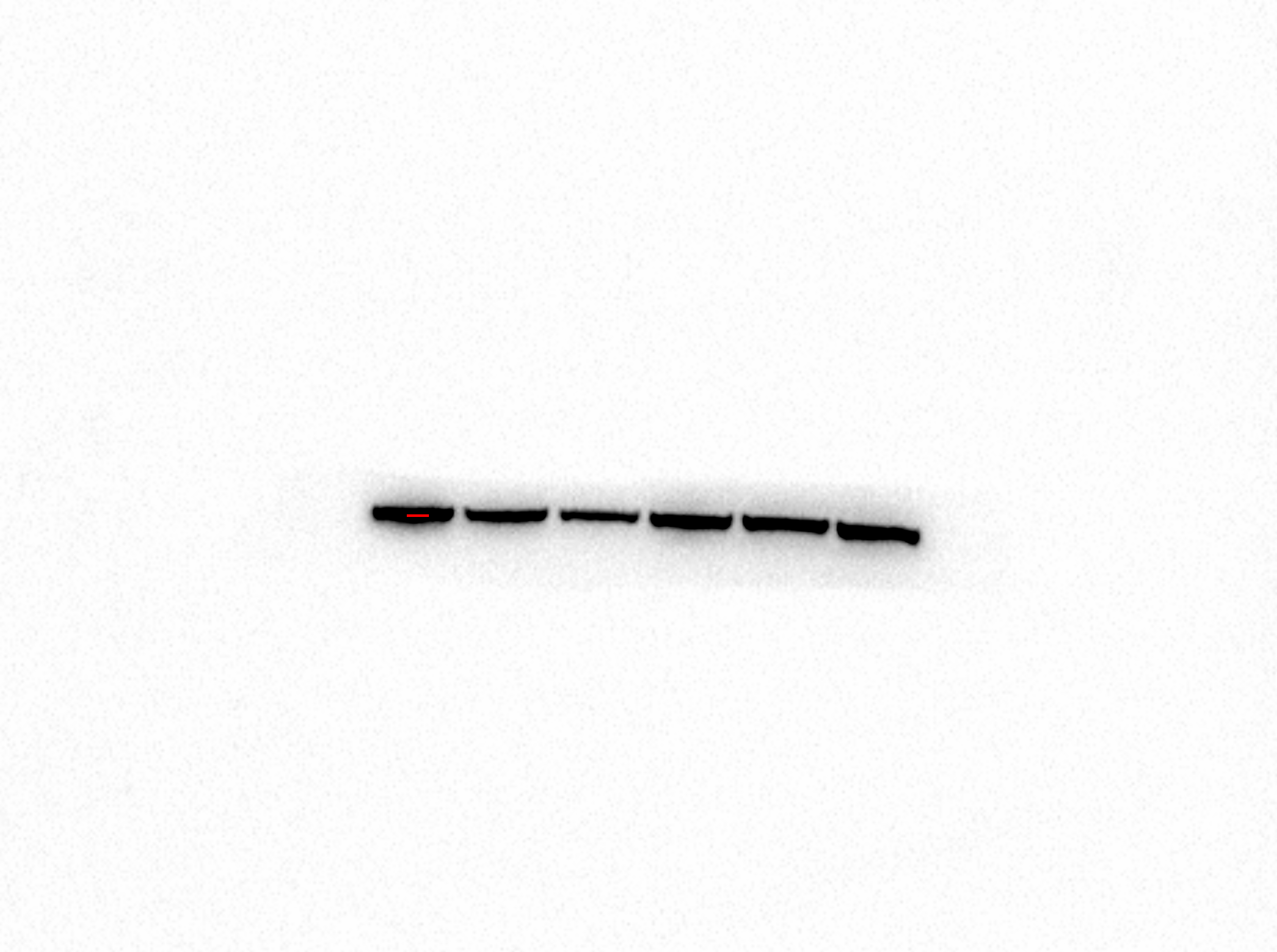

Supplement: Supplementary file 5 [file DataSheet2.ZIP › Original images--WB /2 B-actin.tif]

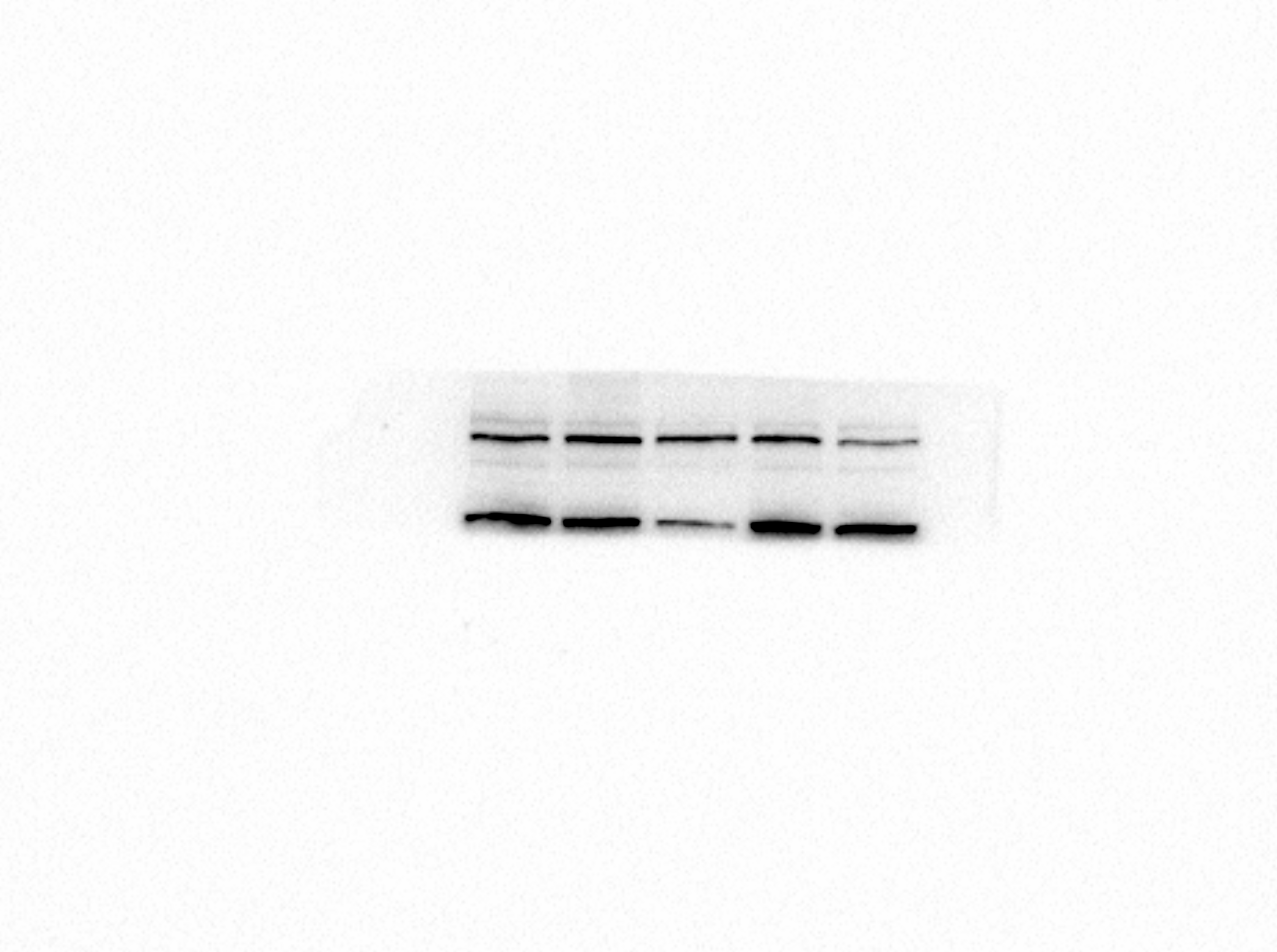

Supplement: Supplementary file 5 [file DataSheet2.ZIP › Original images--WB /h89-PGC1A.tif]

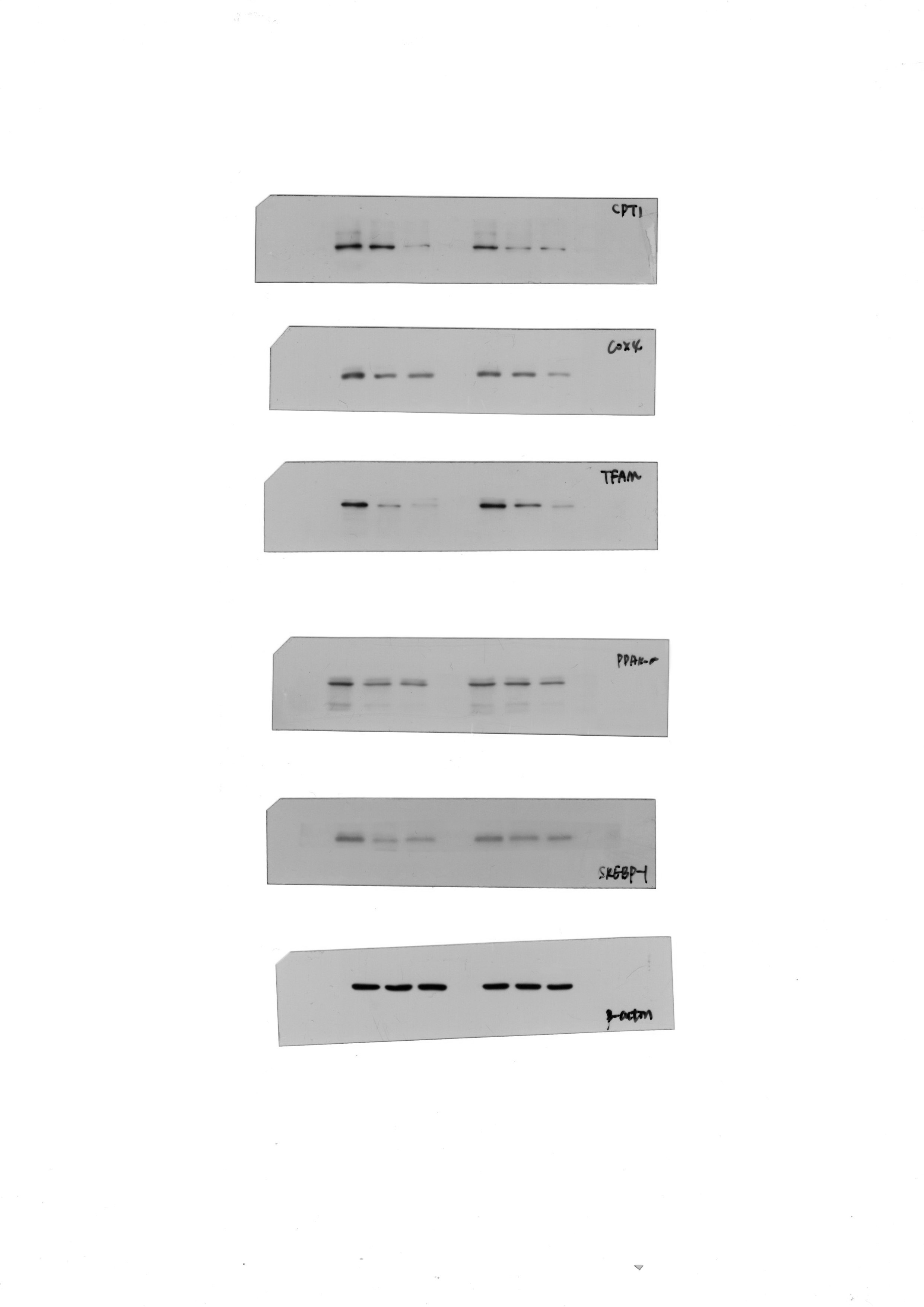

Supplement: Supplementary file 5 [file DataSheet2.ZIP › Original images--WB /SREBP1-grey.jpg]

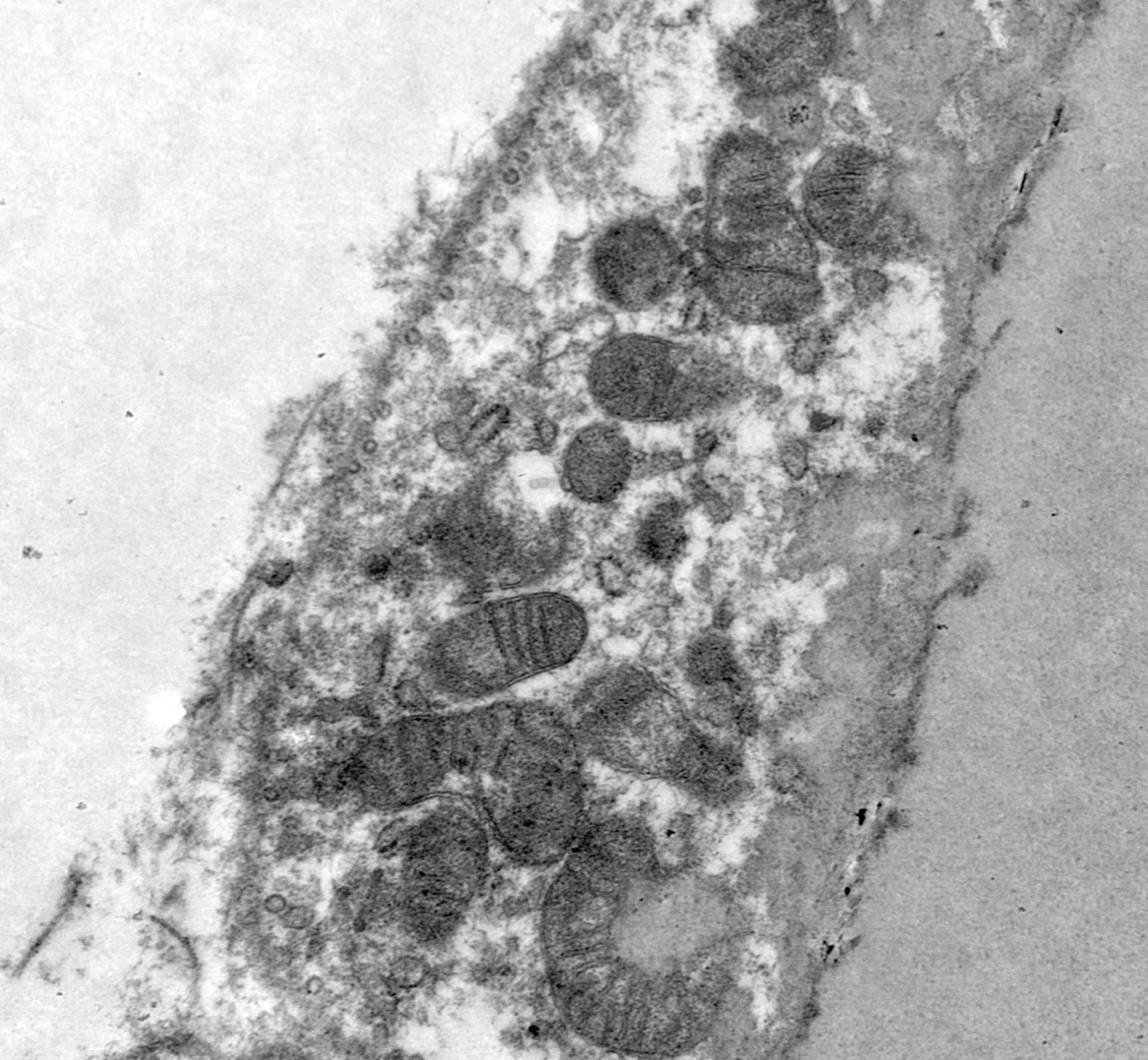

Supplement: Supplementary file 6 [file DataSheet5.ZIP › Original images-electron microscopy-iWAT/NCD.jpeg]

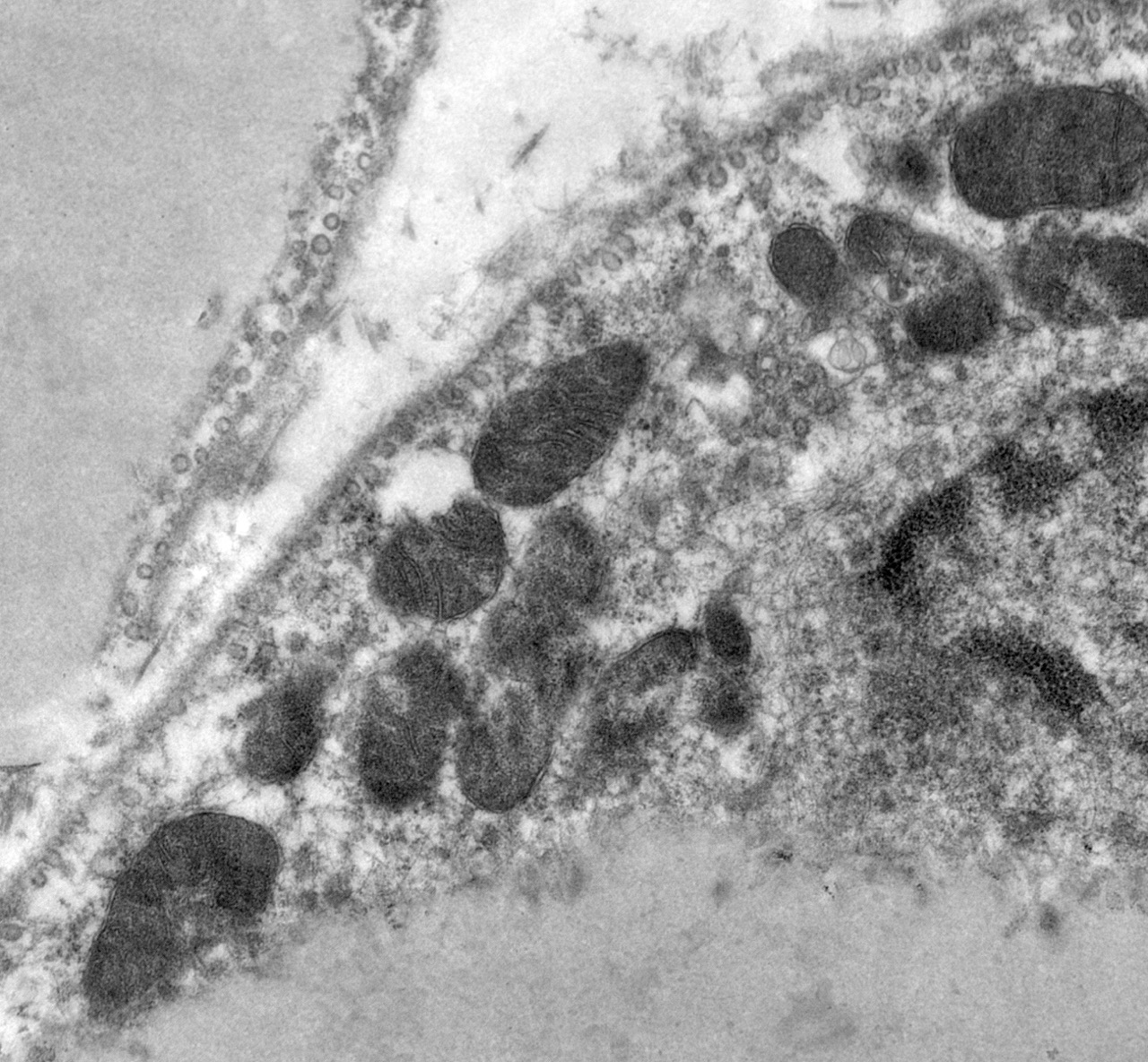

Supplement: Supplementary file 6 [file DataSheet5.ZIP › Original images-electron microscopy-iWAT/HFD+DZF-H.jpeg]

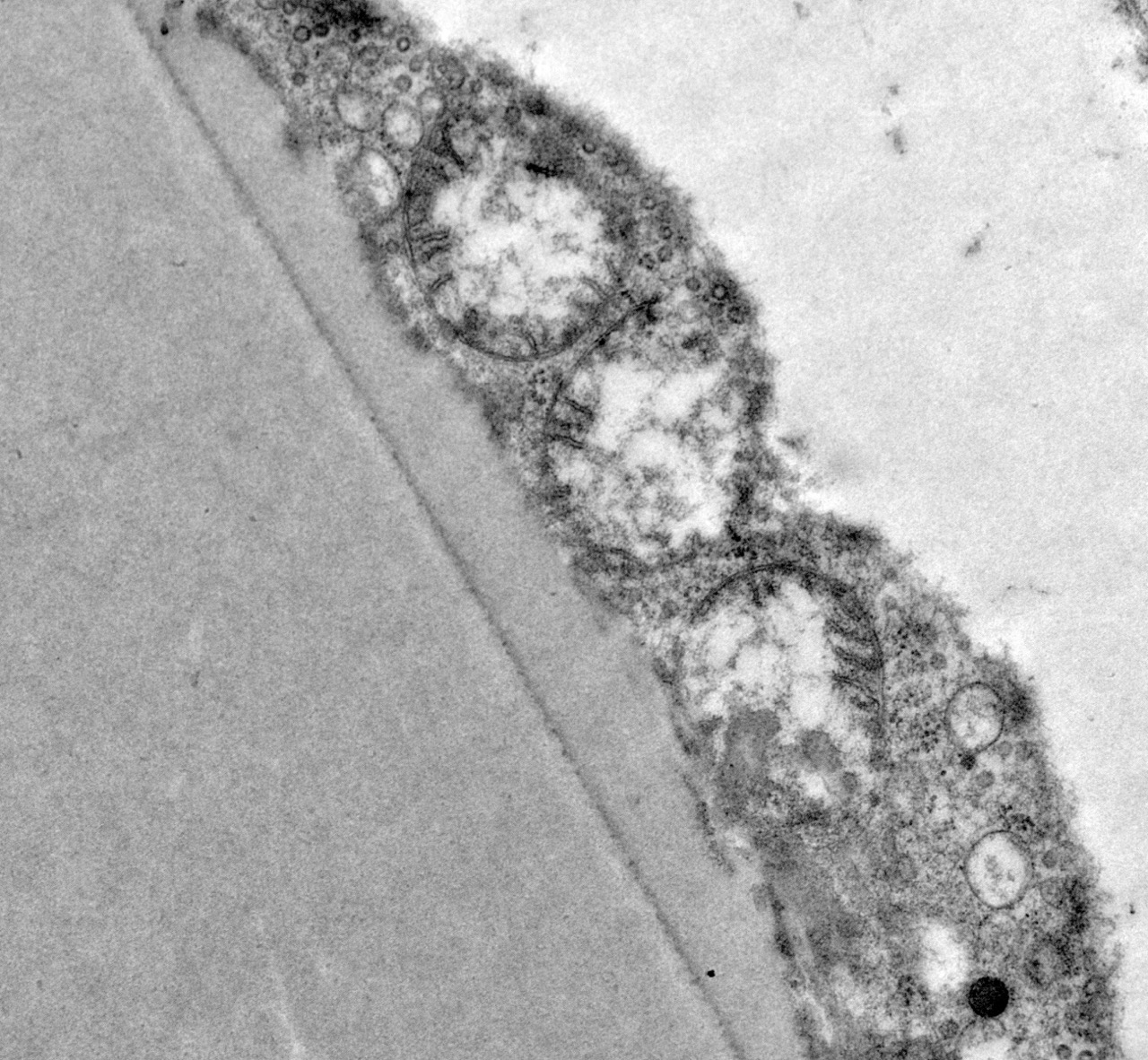

Supplement: Supplementary file 6 [file DataSheet5.ZIP › Original images-electron microscopy-iWAT/HFD+Veh.jpeg]
